# Cu–Ag interactions in bimetallic Cu–Ag catalysts enhance C_2+_ product formation during electrochemical CO reduction†

**DOI:** 10.1039/d4ta04263h

**Published:** 2024-12-05

**Authors:** Floriane A. Rollier, Valery Muravev, Nikolay Kosinov, Tim Wissink, Dimitra Anastasiadou, Bianca Ligt, Laurent Barthe, Marta Costa Figueiredo, Emiel J. M. Hensen

**Affiliations:** a Laboratory of Inorganic Materials and Catalysis, Department of Chemical Engineering and Chemistry, Eindhoven University of Technology P.O. Box 513 5600 MB Eindhoven The Netherlands e.j.m.hensen@tue.nl; b Synchrotron SOLEIL L'Orme des Merisiers, Départementale 128 91190 Saint-Aubin France

## Abstract

The electroreduction of CO (CORR) is a promising alternative to the direct CO_2_ electroreduction reaction (CO2RR) to produce C_2+_ products. Cu-based electrocatalysts enable the formation of C–C bonds, leading to various C_2+_ hydrocarbon and oxygenate products. Herein, we investigated how the composition of bimetallic Cu–Ag catalysts impacted the nature of the Cu–Ag interactions and the product distribution of the CORR, aiming to improve the selectivity to C_2+_ products. Cu–Ag catalysts containing 1–50 mol% Ag were prepared by sol–gel synthesis. A Ag content of 10 mol% of Ag (Cu_0.9_Ag_0.1_) was optimum with respect to increasing the C_2+_ product selectivity and suppressing H_2_ evolution. *Operando* X-ray absorption spectroscopy and quasi-*in situ* X-ray photoelectron spectroscopy demonstrated the complete reduction of CuO to Cu during CORR. Electron microscopy (EM) and *in situ* wide-angle X-ray scattering (WAXS) revealed substantial restructuring during reduction. EM imaging showed the formation of Ag–Cu core–shell structures in Cu_0.9_Ag_0.1_, while separate Cu and Ag particles were predominant at higher Ag content. *In situ* WAXS revealed the formation of a Cu–Ag nanoalloy phase in the bimetallic Cu–Ag samples. The optimum Cu_0.9_Ag_0.1_ sample contained more Cu–Ag nanoalloys than samples with a higher Ag content. The Cu–Ag interfaces between the Ag-core and the Cu-shell in the bimetallic particles are thought to host the nanoalloys. The optimum CORR performance for Cu_0.9_Ag_0.1_ is likely due to the enhanced Cu–Ag interactions, as confirmed by a sample prepared with the same surface composition by galvanic exchange.

## Abstract 2

The interactions between Cu and Ag, such as in Cu–Ag nanoalloys, are crucial to enhance the selectivity to C_2+_ products in the electroreduction of CO (CORR).

## Graphical abstract

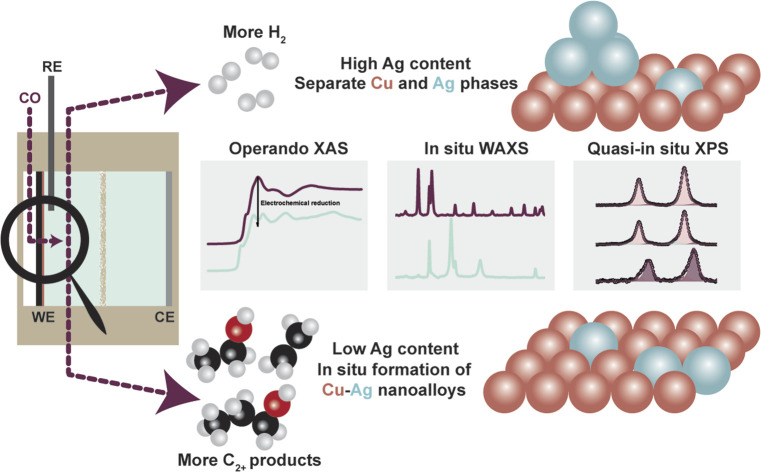

## 1 Introduction

The electrochemical reduction reaction of carbon monoxide (CORR) to products containing C–C bonds (C_2+_ products) has seen increasing scientific interest in recent years.^1–6^ Mechanistic investigations of the related CO_2_ electroreduction reaction (CO2RR) showed the pivotal role of CO as the critical surface intermediate in the formation of C–C bonds in C_2+_ products.^7–10^ Consequently, replacing CO_2_ with CO as the carbon source may enhance the selectivity to C_2+_ compounds. Previous reports confirmed this by showing a higher selectivity for products like ethylene, ethanol, and acetate using CO instead of CO_2_.^3,11,12^ In a two-step strategy, the reduction of CO_2_ to CO, involving a two-electron transfer, would first be carried out, followed by the conversion of CO to C_2+_ products. While for CO2RR, a C=O dissociation step is necessary to proceed to C–C coupling, this is not the case for CORR. Moreover, forming a mole of product from CO requires fewer electrons than CO2RR. These two factors may favor the formation of C_2+_ products in CORR, as reported in some recent studies.^6,13^

Catalyst design approaches are increasingly employed to improve the selectivity of thermal and electrochemical catalytic processes.^14–19^ In his early work, Hori screened many metals for CO2RR and CORR in search of a suitable metal-selectivity descriptor.^20^ Among the investigated metals, only Cu-based catalysts could convert CO_2_ and CO to C_2+_ products.^10,20,21^ The typical product distribution on Cu includes, among other products, ethylene, propylene, ethanol, propanol, and acetic acid. Yet, the similar mechanistic pathways shared among these products result in poor selectivity of the CORR^10,22,23^ and impede the further development of the technology towards practical applications.^24^

Control over the morphology and composition of electrocatalysts is often employed to tune their performance towards CO2RR and CORR.^14,25–27^ Adding another metal to Cu alters the product distribution by modifying the surface sites and the binding properties of reaction intermediates. Metals selective to CO formation, such as Au, Ag, and Zn, have been used as co-catalysts in CO2RR to enable sequential electroreduction of CO_2_ to CO, followed by the conversion of CO to target products.^27–31^ In a tandem fashion, CO is primarily produced on the co-catalyst, and the spillover of CO to Cu results in high reactant coverage. The abundance of CO intermediate in the double layer facilitates C–C coupling reactions, enhancing the selectivity to C_2+_ products.^27–31^ Moreover, interactions between Cu and CO-selective metals were beneficial, as interfacial sites and the formation of alloys improve the selectivity to C_2+_ products in CO2RR.^30,32–36^ For instance, Huang *et al.* developed Cu–Ag nanodimers possessing tandem and interfacial catalysis sites. At the interfacial sites, the formation of a Cu–Ag nanoalloy, hosting electronic effects between Cu and Ag, was beneficial for the formation of C_2+_ products.^37^

Earlier works on CORR focused exclusively on bimetallic catalysts prepared by galvanic exchange.^19,38,39^ This method implies the spontaneous replacement of surface Cu by Ag upon immersion of Cu metal in a solution containing Ag^+^ ions.^38,39^ Some reports mentioned that the presence of Ag in a surface alloy maintains part of the Cu atoms in a Cu^*δ*+^ state.^33,40^ CO molecules bind more strongly on these sites, which increases the CO residence time on the surface and, therefore, the probability of C–C coupling reactions. Conversely, other reports emphasized the fully reduced state of Cu–Ag catalysts, which was observed almost immediately after applying a negative potential.^19,38,39^ Some of these works correlated the better performance to strain and ligand effects, which reduce the activation energy of C_1_ to C_1_ and C_1_ to C_2_ coupling, widen the d-band, and promote electron transfer from Cu to Ag.^19,38,39^ As the nature of Cu–Ag interfacial sites under CORR conditions remains elusive, *in situ* characterization is required to confirm their presence. Moreover, the role of bimetallic Cu–Ag electrocatalysts for CORR has only been scarcely investigated.^19,38,39^ As a result, the nature of the active phase, its oxidation state, and possible restructuring under reaction conditions are valuable topics of investigation.

In this study, we synthesized bimetallic Cu–Ag catalysts using a one-pot sol–gel method to increase the interactions between the two metals. The Cu–Ag composition substantially impacted the selectivity to C_2+_ products during CORR. In particular, the C_2+_ product selectivity was the highest (faradaic efficiency 63%) for the sample containing 10 mol% Ag. While *ex situ* characterization by XPS clearly demonstrated differences in Cu–Ag interactions in the as-prepared samples of different composition, *operando* XAS and *in situ* WAXS highlighted the structural differences between the reduced samples during the CORR. The addition of 10 mol% Ag in Cu_0.9_Ag_0.1_ caused the expansion of the Cu lattice due to the formation of nanoalloys. STEM-EDX imaging of used samples showed the formation of Ag–Cu core–shell structures at low Ag content, while mostly separated Cu and Ag particles were observed at high Ag content (50 mol% Ag). The extent of Cu–Ag interactions explained the catalytic performance differences as a function of the Ag content.

## 2 Results and discussion

### 2.1. CORR performance

**Fig. 1:**
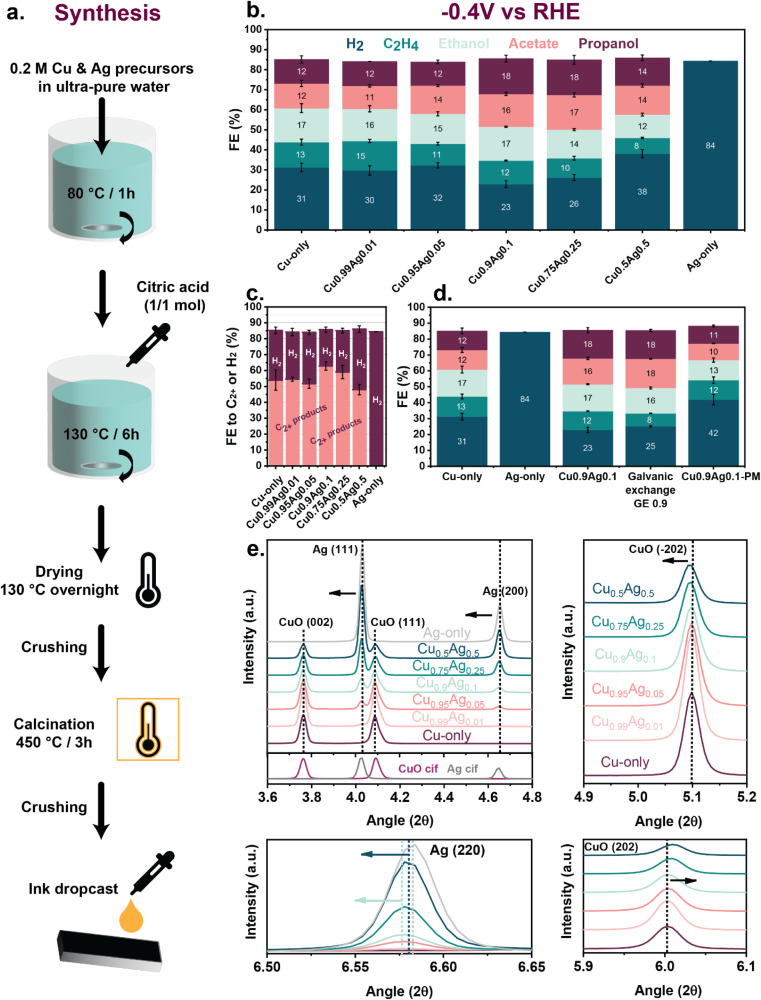
(a) Sol–gel synthesis method employed to prepare bimetallic Cu–Ag, Cu-only and Ag-only samples, (b) faradaic efficiencies of the sol–gel samples measured at −0.4 V *vs.* RHE, CORR, 1 h, 3 M KOH, (c) faradaic efficiencies to C_2+_ products and H_2_ of the sol–gel samples measured at −0.4 V *vs.* RHE, CORR, 1 h, 3 M KOH, (d) Comparison of the faradaic efficiencies of the sol–gel and reference samples prepared by galvanic exchange and physical mixing measured at −0.4 V *vs.* RHE, CORR, 1 h, 3 M KOH, (e) X-ray diffractograms of the as-prepared samples (WAXS, *λ* = 0.0165312 nm).

Several Cu–Ag bimetallic catalysts of different compositions (Ag content 1–50 mol%) and monometallic references (Cu-only and Ag-only) were prepared using a one-pot sol–gel synthesis method (Fig. 1a). The composition of the obtained samples was confirmed by elemental analysis (inductively coupled plasma-optical emission spectrometry (ICP-OES)) (Table S2†). The performance of these samples was tested in CORR under strongly alkaline conditions (3 M KOH). At a mild potential of −0.4 V *vs.* RHE (Fig. 1b, S7 and Note S1†), the Cu-only sample displayed a faradaic efficiency (FE) of 31% towards H_2_, while the FE to C_2+_ products was 54%. Among the C_2+_ products, ethylene, ethanol, and acetate were formed with FEs of 13%, 12%, and 17%, respectively. Such a product distribution is typical for Cu electrocatalysts measured under alkaline conditions.^38,39^ Increasing the Ag content up to 5 mol% in the Cu_0.95_Ag_0.05_ sample did not significantly influence the FEs, as a similar product distribution was observed compared to the Cu-only catalyst. In contrast, adding 10 mol% of Ag suppressed H_2_ and enhanced the C_2+_ product formation, leading to FEs of 23% and 63%, respectively, for the Cu_0.9_Ag_0.1_ sample. The FE to propanol, in particular, reached a high value of 18% for this composition. Among recent CORR works, only the groups of Sargent and Jiao reported high propanol FE on Cu–Ag bimetallic catalysts.^12,38^ Most of the literature, however, reported a propanol FE below 5%,^11,19,39^ revealing the challenging nature of chain growth during CORR. The C_2+_ product FE decreased with increasing further the Ag content, with FEs of 59% and 48% for Cu_0.75_Ag_0.25_ and Cu_0.5_Ag_0.5_, respectively (Fig. 1c). The Ag-only catalyst produced only H_2_, showing the negative impact of high Ag content on C_2+_ formation. For completeness, the minor side-products formed on all samples are shown in Fig. S6.†

The Cu-only and Cu_0.9_Ag_0.1_ samples were also tested at two other potentials, namely −0.5 V and −0.6 V *vs.* RHE (Fig. S8†). The comparison of these two samples at these more negative potentials emphasized the selectivity trends discussed before. At potentials of −0.5 V and −0.6 V *vs.* RHE, Cu_0.9_Ag_0.1_ demonstrated a higher selectivity to C_2+_ products than the Cu-only sample. The C_2+_ product FE reached 45% on Cu_0.9_Ag_0.1_, whereas it declined to 28% for Cu-only at −0.6 V *vs.* RHE. Our data suggest that different active sites are present in the Cu_0.9_Ag_0.1_ and Cu-only samples and that the presence of Cu–Ag interactions in the former catalyst benefits C–C coupling reactions and suppresses the competing hydrogen evolution reaction (HER).

### 2.2. Comparison of sol–gel Cu_0.9_Ag_0.1_ with reference samples

The observed CORR selectivity trends point to a synergy between Cu and Ag for the Cu_0.9_Ag_0.1_ sample. To better understand the nature of the Cu–Ag interactions formed during sol–gel synthesis, the CORR performance of the Cu_0.9_Ag_0.1_ sample was compared to two reference samples of the same composition obtained by (i) galvanic exchange of Cu with Ag^38^ and (ii) physical mixing of the Cu-only and Ag-only samples (Fig. 1d and S9†). Galvanic exchange can be used to synthesize bimetallic Cu–Ag catalysts with strong surface interactions, while physical mixing results in catalysts where the phases are physically isolated.^28,32,39,41^ As the Ag atoms replace only the surface Cu atoms during galvanic exchange, it is not likely that Ag atoms would be present in the bulk of the Cu phase. The surface composition of the galvanically exchanged reference (referred to as GE 0.9) was chosen to match the surface composition of Cu_0.9_Ag_0.1_ after reduction based on quasi-*in situ* XPS data, and the synthesis parameters were adapted accordingly (Table S7†). The physically mixed sample (referred to as Cu_0.9_Ag_0.1_-PM) was prepared by mixing the Cu-only and Ag-only samples, both obtained by sol–gel synthesis, in the appropriate ratio. The product distribution of Cu_0.9_Ag_0.1_-PM, at −0.4 V *vs.* RHE, resembled the one of the Cu-only sample (Fig. 1d). The increase in H_2_ production can be explained by the presence of separate Ag particles and the absence of synergetic interactions between the Cu and Ag phases. More importantly, the Cu_0.9_Ag_0.1_ and GE 0.9 displayed a similar FE for oxygenates, *i.e.*, 49% and 52%, respectively. Based on this observation, we speculate that the Cu–Ag interactions in Cu_0.9_Ag_0.1_ resemble those in GE 0.9, suggesting the formation of a surface Cu–Ag nanoalloy in Cu_0.9_Ag_0.1_.^32,42^

### 2.3. Cu–Ag interactions in the as-prepared samples

*Ex situ* wide-angle X-ray scattering (WAXS) was employed to determine the crystalline phase composition and crystallite size of the as-prepared samples. The as-synthesized samples comprised monoclinic CuO and face-centered cubic (fcc) metallic Ag phases (Fig. 1e and S13a†).^36,43–45^ The shift of all Ag reflections to lower diffraction angles in the Cu–Ag bimetallic samples indicated an expansion of the Ag lattice compared to the Ag-only reference (Table S4 and Fig. S14†). The largest shift of the Ag (220) and (200) reflections was observed for Ag contents of 5 and 10 mol%. Furthermore, some CuO reflections in the bimetallic samples shifted to higher angles (*e.g.*, CuO (110), CuO (002), CuO (202)) and lower angles (*e.g.*, CuO (−202)), pointing to CuO lattice distortions (Fig. S14†). These observations demonstrate a prominent effect of the bulk composition on the monoclinic CuO and fcc Ag lattice parameters.

**Fig. 2:**
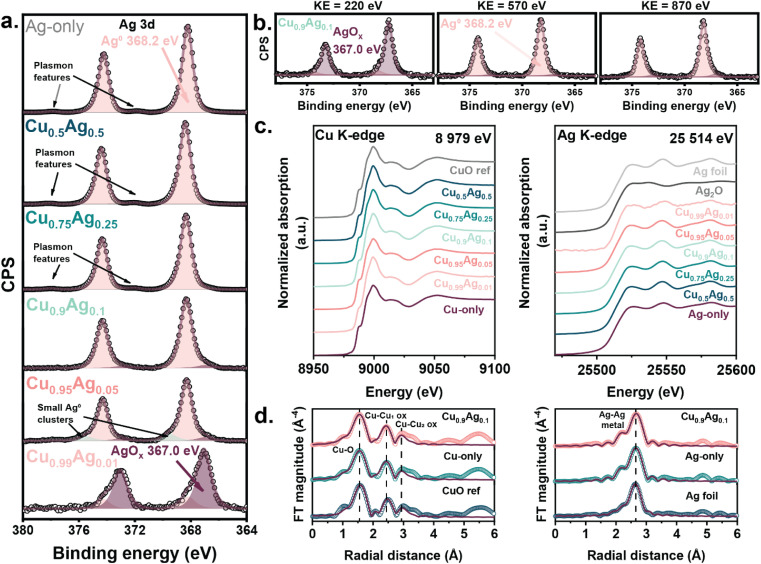
(a) XPS spectra of the as-prepared samples (monochromatic Al Kα = 1486.6 eV), (b) depth profile of Cu_0.9_Ag_0.1_ measured by synchrotron-based XPS at various kinetic energies, (c) Cu K-edge XANES and Ag K-edge XANES spectra of the as-prepared samples, and (d) Cu and Ag *k*^3^-weighted FT-EXAFS of the as-prepared samples.

Similarly, the surface speciation of the samples investigated by XPS depended strongly on the composition (Fig. 2a and S17a†). The XPS spectrum of the Ag-only sample was characteristic of metallic Ag (Ag 3d_5/2_ binding energy (BE) at 368.2 eV,^46,47^ spin–orbit splitting 6 eV (ref. 46)), and the presence of plasmon features indicated the presence of large Ag particles.^48^ The Ag 3d spectra of Cu_0.5_Ag_0.5_ and Cu_0.75_Ag_0.25_ resembled the spectrum of the Ag-only sample and plasmon features were present, confirming the large size of metallic Ag domains in these samples. At lower Ag content (Cu_0.9_Ag_0.1_), Ag species were present as a mixture of metallic Ag and AgO_*x*_ (Ag 3d_5/2_ BE at 367.0 eV).^49^ The absence of plasmon features implies that the Ag particles in Cu_0.9_Ag_0.1_ are relatively small. Decreasing the Ag content further led to the formation of small Ag clusters in Cu_0.95_Ag_0.05_ (Ag 3d_5/2_ BE at 369.5 eV (ref. 50)) and a nearly fully oxidized surface in Cu_0.99_Ag_0.01_. In samples containing less than 10 mol% Ag, the formation of AgO_*x*_ next to metallic Ag shows the abundance of small Ag domains, which are readily oxidized. A high Ag dispersion implies an increasing number of CuO–Ag interfaces. Cu–Ag interfaces have been reported to enhance the C_2+_ product selectivity.^37,39^ The Cu 2p_3/2_ spectra of all samples displayed the characteristic features of CuO (Cu 2p_3/2_ BE at 933.5 eV, satellite features from 940–945 eV) (Fig. S17a†). The area ratio of the main line and the satellite was equal to 2, which confirms the sole presence of Cu^2+^.^51^

The speciation of Ag at the surface was further investigated using synchrotron-based XPS (Fig. 2b). As the inelastic mean free path of the emitted photoelectrons depends on their kinetic energy, the possibility of tuning the energy of the incoming X-rays allows measuring at various depths of the surface layer. At low excitation energy, only photoelectrons originating from atoms at the very surface have sufficient kinetic energy to escape the sample (kinetic energy, KE = 220 eV). Increasing the excitation energy leads to probing atoms located deeper in the layer, for which 570 and 870 eV kinetic energies were used. By selecting different excitation energies, we could probe Cu and Ag at the same kinetic energy, meaning the atoms were located at the same depth (Fig. S17c†). At KE = 220 eV, the surface region of the Cu_0.9_Ag_0.1_ sample was mainly composed of AgO_*x*_ (Ag 3d_5/2_ BE at 367.0 eV) and only a small amount of metallic Ag (Ag 3d_5/2_ BE at 368.2 eV (ref. 49)). Conversely, the Cu_0.75_Ag_0.25_ and Cu_0.5_Ag_0.5_ samples did not display any oxidized Ag species at KE = 220 eV, with the spectra being dominated by metallic Ag species (Fig. S17b†). In line with the lab-based XPS, the plasmon features in the spectra of Cu_0.75_Ag_0.25_ and Cu_0.5_Ag_0.5_ confirmed the presence of large metallic Ag particles.^48^ These data suggest that the Ag domains in Cu_0.9_Ag_0.1_ are smaller and better dispersed than in samples containing more Ag. At greater depths, KE of 570 and 870 eV, the Cu_0.9_Ag_0.1_ sample displayed a prominent metallic Ag signal and a small amount of AgO_*x*_ (Fig. 2b). The absence of plasmon features and the presence of oxidized species at all tested KEs confirmed that small and dispersed Ag domains are present at the surface of Cu_0.9_Ag_0.1_. In contrast, large Ag particles are present in samples containing a higher Ag content.^48^ Moreover, the atomic percentage of Ag in Cu_0.9_Ag_0.1_ at shallow depths (KE 220 eV) was higher than in deeper locations, which correlates with the high dispersion of the Ag phase (Fig. S17c†).

The lattice parameters of the monoclinic CuO and fcc Ag phases were determined by Rietveld refinement of the WAXS data. As-prepared Cu_0.9_Ag_0.1_ showed larger CuO lattice parameters than the other samples (Fig. S15, 16 and Table S9†). In agreement with the peak shift discussed earlier, the Ag lattice was the most expanded in Cu–Ag samples containing less than 10 mol% Ag. The expansion of the Ag lattice seen by WAXS cannot be explained by the substitution of Ag (atomic radius 144 pm) with metallic Cu (atomic radius 128 pm).^52^ Instead, we attribute this to the formation of an interfacial phase between the metallic Ag domains and the oxidized CuO domains. The formation of Cu–Ag mixed oxides (*e.g.* AgCuO_2_ or Ag_2_Cu_2_O_3_) at the interface of oxidized CuO and reduced Ag phases has been discussed before.^53,54^ Due to the similar atomic arrangement of the CuO (−111) and Cu_4_O_3_ (202) planes,^55^ it has been suggested that CuO is modified into a Cu_4_O_3_-like structure along the (−111) plane, which is able to form such mixed oxides with Ag.^19,47,56^ These modifications also impact the CuO lattice parameters as evidenced by Rietveld refinement (Fig. S15, 16 and Table S9†). Moreover, the oxidation of Ag domains at the CuO–Ag interface (Cu–Ag mixed oxides), also evidenced by XPS, is likely responsible for the increase in fcc Ag lattice parameter. In support of this, diffractograms of samples with low Ag content (*e.g.*, Cu_0.95_Ag_0.05_, Fig. S13b†) were found to contain traces of another Cu-containing phase, corresponding to either cubic CuO or a Cu–Ag mixed oxide phase (*e.g.* AgCuO_2_ or Ag_2_Cu_2_O_3_).^47,56,57^ The occurrence of cubic CuO is unlikely as this phase does not exist naturally and has rarely been successfully synthesized.^57^ Thus, based on WAXS and XPS data, we believe that the formation of Cu–Ag mixed oxides at the interface between the Ag and CuO phases caused the changes in peak positions and lattice parameters in the Cu–Ag bimetallic samples, especially in samples with low Ag content (≤10 mol%).

X-ray absorption near-edge spectroscopy (XANES) at the Cu and Ag K-edges was employed to probe the bulk oxidation state of the as-prepared samples. Cu^2+^ from CuO and metallic Ag were observed in line with the other characterization methods (Fig. 2c).^40,58,59^ The local environment of Cu and Ag was investigated by analyzing the extended X-ray absorption fine structure (EXAFS) region of the XAS spectra (Fig. 2d), which agreed with the presence of CuO^60^ and Ag.^61^ The coordination number of Ag increased from 8.8 ± 0.6 for the Cu_0.99_Cu_0.01_ sample to 11.9 ± 0.4 for the Ag-only sample, in agreement with the different Ag dispersion noted above (Table S5†).

**Fig. 3:**
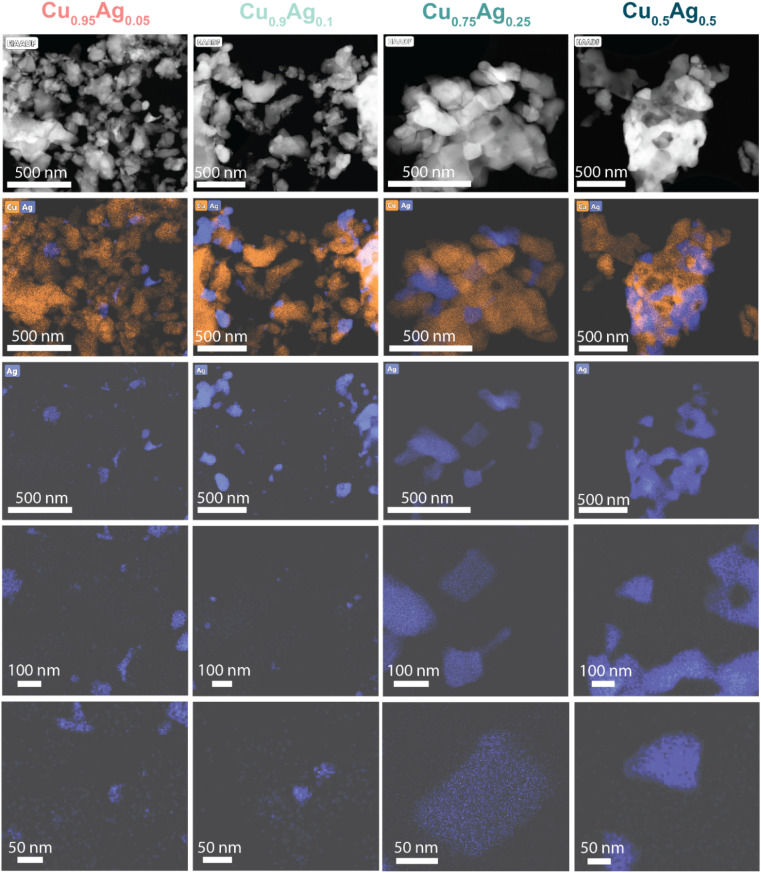
HAADF-STEM images and corresponding EDX maps of the as-prepared Cu_0.95_Ag_0.05_, Cu_0.9_Ag_0.1_, Cu_0.75_Ag_0.25_ and Cu_0.5_Ag_0.5_ samples.

HAADF-STEM images and EDX mapping (Fig. 3 and S18†) showed that all as-prepared samples contained CuO particles, ranging from a few hundred nanometers to sub-micrometer size regardless of the Ag content. On the contrary, the Ag dispersion strongly depended on the Ag content. In the Cu_0.5_Ag_0.5_ sample, Ag was predominantly present as 100–500 nm particles. In contrast, the Cu_0.95_Ag_0.05_ and Cu_0.9_Ag_0.1_ samples also contained much smaller Ag particles measuring only a few nanometers (Fig. 3). It is reasonable to speculate that these smaller Ag particles are present due to CuO–Ag interactions. To support this, certain regions showed decoration of Ag particles by a shell of CuO, confirming the formation of interactions at the CuO–Ag interfaces during the synthesis of samples with low Ag content (Fig. S19†). These observations align with the XPS results, which showed the higher dispersion of Ag species at low Ag content. As expected, the surface replacement of Cu in the galvanically exchanged GE 0.9 sample led to strong Cu–Ag interaction and dispersed Ag atoms (Fig. S20†). As the catalytic performances of Cu_0.9_Ag_0.1_ and GE 0.9 are similar, we speculate that the Cu–Ag interfacial sites in Cu_0.9_Ag_0.1_ under CORR are similar to GE 0.9 and are crucial for forming C_2+_ products.

### 2.4. Cu–Ag interactions during CORR

**Fig. 4:**
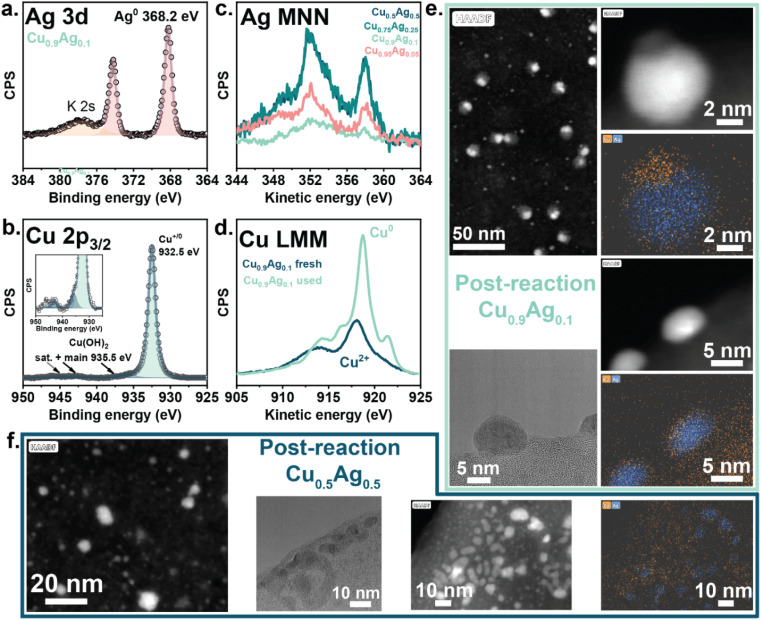
Quasi-*in situ* XPS spectra of the (a) Ag 3d and (b) Cu 2p_3/2_ lines of Cu_0.9_Ag_0.1_ after chronoamperometry at −0.4 V *vs.* RHE for 10 min, (c) Quasi-*in situ* XPS spectra of the Ag MNN lines of several Cu–Ag samples after chronoamperometry at −0.4 V *vs.* RHE for 10 min, (d) Quasi-*in situ* XPS spectra of the Cu LMM lines of Cu_0.9_Ag_0.1_ before and after chronoamperometry at −0.4 V *vs.* RHE for 10 min, (e) and (f) HAADF-STEM images and EDX maps of used (e) Cu_0.9_Ag_0.1_ and (f) Cu_0.5_Ag_0.5_ after CORR at −0.4 V *vs.* RHE, 1 h, 3 M KOH.

Although *ex situ* characterization provided insights into the CuO–Ag interactions in the as-prepared state of the sample, electroreduction can lead to phase transformations and very different morphologies, which can be best studied using *in situ* techniques.^34,40,42^ We used quasi-*in situ* XPS to investigate the surface oxidation state and composition after the reduction of the samples at −0.4 V *vs.* RHE in 3 M KOH (Fig. 4a–d, S21a–c and Table S6†). These measurements were done in an XPS system that allows transferring the samples from the electrochemical cell to the XPS analysis chamber under an inert atmosphere (He), preventing post-reaction oxidation by air exposure. The surface of all samples was fully reduced after 10 min of reduction at −0.4 V *vs.* RHE (Fig. 4a–d and S21†), as shown by the Cu 2p_3/2_ line at BE 932.5 eV, the Cu LMM Auger line shape, the Ag 3d_5/2_ line at BE 368.2 eV and the Ag MNN Auger line shape.^51,56,62^ The minor contribution of Cu(OH)_2_ (Cu 2p_3/2_ line at BE 935.5 eV, satellite at BE 945 eV (ref. 51)) likely originates from the reaction of the sample with KOH upon drying or exposure to the open circuit potential (OCP).^63,64^ Cu_0.5_Ag_0.5_ and Cu_0.75_Ag_0.25_ were composed of large Ag domains, as follows from the plasmon features.^48^ The absence of such features in the spectra of Cu_0.9_Ag_0.1_ and Cu_0.95_Ag_0.05_ showed that these samples contained much smaller Ag domains than samples with a high Ag content. The surface Ag/Cu ratio in Cu_0.9_Ag_0.1_ decreased during the reduction (Table S6†) and was the lowest among the investigated samples. The low surface Ag content and the high dispersion of the Ag domains in Cu_0.9_Ag_0.1_ points to the abundance of Cu–Ag interfacial sites. These interfaces are thought to induce electronic effects between Cu and Ag, modifying the binding strength of intermediates relevant to C_2+_ product formation and, hence, the selectivity to such products.^37–39^ This can explain the more significant formation of C_2+_ products on the Cu_0.9_Ag_0.1_ sample.

HAADF-STEM and EDX mapping revealed a composition-dependent reorganization of Cu and Ag phases during CORR (Fig. 4e–f and S22†). Contrasting with the dimensions of the as-prepared samples, the used Cu_0.9_Ag_0.1_ and Cu_0.5_Ag_0.5_ samples contained particles measuring less than 20 nm. After the reaction, Cu_0.9_Ag_0.1_ comprised 2–15 nm Cu–Ag bimetallic particles with an off-centered core–shell structure.^65,66^ Such morphologies give rise to extended Cu–Ag interfaces, which correlates with the XPS results. The Z-contrast HAADF-STEM and EDX-mapping revealed that Ag resides in the core of polycrystalline particles, while Cu was predominantly present in the shell. We should note that in Cu_0.9_Ag_0.1_, the Ag–Cu core–shell structures coexisted with ∼3 nm Cu particles, seemingly not interacting with Ag. Conversely, the used Cu_0.5_Ag_0.5_ was predominantly composed of separate metallic Cu and Ag particles and only a small fraction of core–shell structures. The presence of extended Cu–Ag interfaces in Cu_0.9_Ag_0.1_ can explain the enhanced selectivity to C_2+_ products, while H_2_ predominantly evolves on separated phases in the Cu_0.5_Ag_0.5_ sample.^38,39^ The microscopy data of the used GE 0.9 sample revealed that many Cu–Ag interfaces were present after CORR (Fig. S23†). We believe that the nature of Cu–Ag interactions at the interface between the Ag-core and the Cu-shell in Cu_0.9_Ag_0.1_ is similar to the interactions found in the GE 0.9 sample, explaining their similar catalytic performance.

Under CORR conditions, the reduction of the parent CuO and dissolution–redeposition processes can result in the restructuring of the Cu phase.^67–69^ The restructuring of metallic Ag was less expected as the phase remained identical. The size of Ag particles decreased drastically during CORR compared to the as-prepared domains. Yun *et al.* studied the morphological changes of Ag nanoparticles by *in situ* TEM under CO2RR conditions.^70^ The authors demonstrated that the metallic Ag particles decreased in size during the first instances of reduction and subsequently redispersed following a dissolution–redeposition process. As the final structure of the sol–gel-derived samples varied with their composition, it is important to also characterize the reduced samples.

**Fig. 5:**
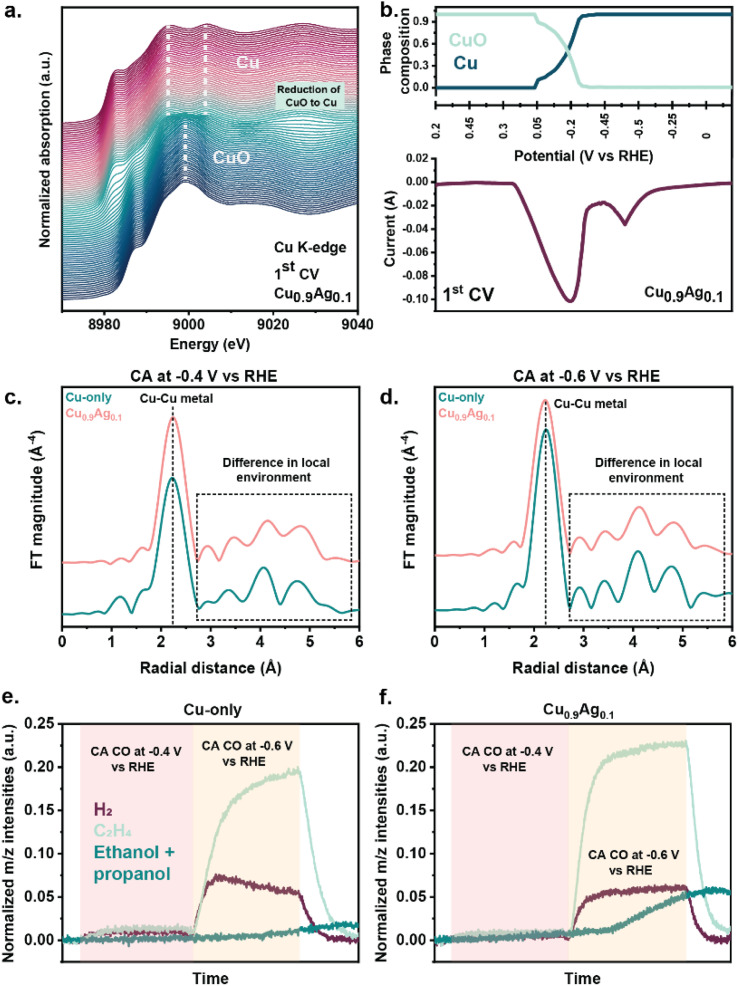
(a) Cu K-edge XANES spectra of Cu_0.9_Ag_0.1_ during the first CV measurement, 3 M KOH, start +0.2 V *vs.* RHE, cycle from +0. 5 V to −0.6 V *vs.* RHE, 5 mV s^−1^, (b) linear combination fitting of spectra recorded during the first CV of Cu_0.9_Ag_0.1_ and the CV signal, 3 M KOH, start +0.2 V *vs.* RHE, cycle from +0.5 V to −0.6 V *vs.* RHE, 5 mV s^−1^, (c) Cu *k*^3^-weighted FT-EXAFS of Cu-only and Cu_0.9_Ag_0.1_ recorded during the CA at −0.4 V *vs.* RHE, (d) Cu *k*^3^-weighted FT-EXAFS of Cu-only and Cu_0.9_Ag_0.1_ recorded during the CA at −0.6 V *vs.* RHE, (e) and (f) normalized *m*/*z* intensities of H_2_, C_2_H_4_, and ethanol + propanol MS signals measured during CA at −0.4 and −0.6 V *vs.* RHE of (e) Cu-only and (f) Cu_0.9_Ag_0.1_ (normalization on the CO *m*/*z* intensity).

X-ray absorption spectroscopy (XAS) was utilized to probe structural and redox transformations during CORR while simultaneously monitoring gaseous products by mass spectrometry. To mimic the experimental protocol used before catalytic testing, cyclic-voltammetry cycles (+0.5 V to −0.6 V *vs.* RHE, 5 mV s^−1^) were recorded while the cell was flushed with N_2_ in a flow-by mode. During the first CV cycle, all samples underwent partial or total reduction from CuO to metallic Cu (Fig. 5a, b, S25–26, S28 and S29†).^58,71^ The presence of isosbestic points indicated a direct reduction from CuO to Cu without the formation of Cu_2_O (Fig. S24†). Therefore, linear combination fitting was applied using only CuO and Cu references to quantify the reduction of each sample. After the first CV, the Cu_0.9_Ag_0.1_ sample was completely reduced, unlike the Cu-only sample (71% Cu^0^). The CV recorded in N_2_ and CO demonstrated that the onset of CuO reduction occurred at lower potentials on Cu_0.9_Ag_0.1_ compared to Cu-only (Fig. S1 and 2†), indicative of synergistic interactions between Cu and Ag in the bimetallic sample. These interactions facilitate the reduction of CuO to Cu. The dependence of the reducibility of CuO on the Ag content has earlier been associated with Cu–Ag interfacial sites.^72^ In the subsequent CV cycles (Fig. S25–28 and S30†), the oxidation state of the Cu species was stable in all samples. Similarly, the Ag species remained metallic upon cycling (Fig. S29c†).

Next, CORR was carried out on each sample at −0.4 V *vs.* RHE and −0.6 V *vs.* RHE (Fig. 5c–f, S31–34 and Table S8†). During these measurements, the Cu_0.9_Ag_0.1_ sample only contained metallic Cu and Ag species, while the reduction of the Cu-only sample was only complete at the end of the chronoamperometric experiment at −0.6 V *vs.* RHE (Fig. S31 and S33†). The Cu *k*^3^-weighted FT-EXAFS showed that, despite the similar metallic Cu–Cu first coordination shell, the Cu-only and Cu_0.9_Ag_0.1_ samples exhibited significantly different coordination environments at longer radial distances (Fig. 5c and d). The differences observed in the Cu *k*^3^-weighted FT-EXAFS of Cu_0.9_Ag_0.1_ likely arise from Cu–Ag interactions. Mass signals (*m*/*z*) of products were followed during the *operando* XAS measurements. Cu_0.9_Ag_0.1_ showed the largest normalized *m*/*z* intensities for ethylene, ethanol, and propanol (Fig. 5e and f), correlating with the highest C_2+_ product FE measured during CORR. As the *operando* XAS data confirmed that Cu species in Cu_0.9_Ag_0.1_ were exclusively present in the metallic state during CORR, the enhanced C_2+_ product selectivity measured on this sample did not originate from traces of Cu^*δ*+^, as suggested for Cu-based electrocatalysts in earlier works.^33,40^ Instead, the analysis of the local environment of Cu atoms in Cu_0.9_Ag_0.1_ revealed Cu–Ag interactions during CORR, which correlate with higher normalized *m*/*z* intensities of the C_2+_ products. This strongly suggests that the higher C_2+_ product selectivity can be explained by CORR reactions at Cu–Ag interfacial sites.

**Fig. 6:**
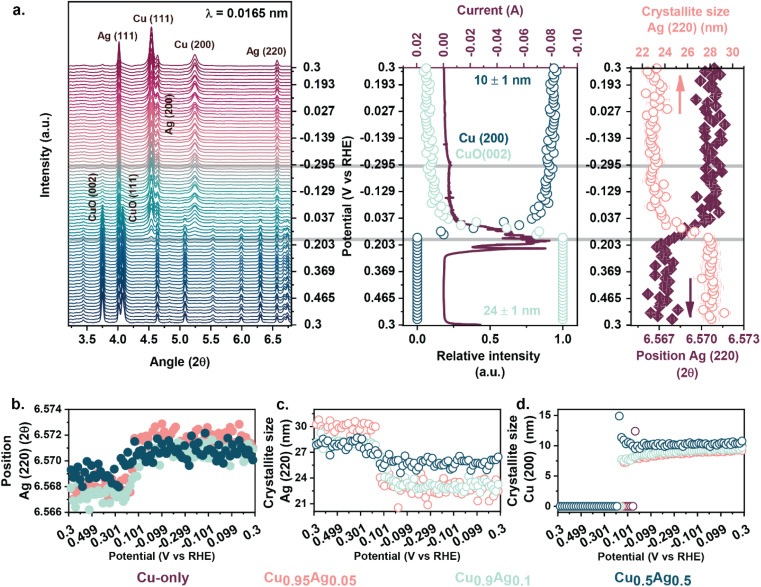
(a) *In situ* WAXS of Cu_0.9_Ag_0.1_ during the first CV cycle (3 M KOH, start +0.3 V *vs.* RHE, cycle from +0.5 V to −0.3 V *vs.* RHE, 2 mV s^−1^) (left panel), corresponding CV and relative intensity of the CuO (002) and Cu (200) reflections recorded during the CV (middle panel), evolution of the Ag crystallite size and peak position of Ag (220) during the CV (right panel), (b) evolution of the Ag crystallite size derived from the Ag (220) reflection, (c) evolution of the peak position of Ag (220) and (d) evolution of the Cu crystallite size derived from the Cu (200) reflection for several Cu–Ag bimetallic and Cu catalysts recorded during the first CV (3 M KOH, start +0.3 V *vs.* RHE, cycle from +0.5 V to −0.3 V *vs.* RHE, 2 mV s^−1^).

*In situ* WAXS was employed to understand the transformations of the crystalline phases upon reduction and CORR. During a first cyclic-voltammogram (CV from +0.3 V to −0.3 V *vs.* RHE, 2 mV s^−1^, N_2_ atmosphere), the Ag-containing samples displayed the complete reduction of crystalline CuO to metallic Cu (Fig. 6a and S35†). At peak cathodic currents, the Cu (111) and (200) reflections were visible in the diffractograms and their integral intensities kept increasing upon subsequent cycling.^73^ In contrast, the Cu reduction was incomplete for the Cu-only sample (Fig. S35†). Similarly to the XAS results, *in situ* WAXS showed that the presence of Ag promoted the reduction of CuO to Cu. As expected, the metallic Ag crystallites in the precursor remained metallic upon cycling. However, the crystallite sizes and the positions of the reflections changed when a negative potential was applied (Fig. 6a–c and S35†). For instance, the crystallite size of Ag (derived from the (220) reflection) in the Cu_0.9_Ag_0.1_ sample decreased from 28 to 23 nm (5 nm) when CuO was reduced to metallic Cu. The Ag domain size stabilized once the reduction of CuO was completed, serving as another indication for CuO–Ag interactions (Cu–Ag mixed oxides) in the as-prepared samples. The largest Ag crystallite size difference was observed for Cu_0.95_Ag_0.05_ (∼7 nm) and the smallest for Cu_0.5_Ag_0.5_ (∼2 nm) (Fig. 6c).

Additionally, the position of the Ag (220) reflection, initially displaced at lower angles compared to the Ag-only sample, shifted back to higher angles upon reduction (Fig. 6b). This shift occurred simultaneously with the loss of CuO reflections, further supporting the strong interactions between the two phases and the presence of mixed oxides in the as-prepared catalysts. The difference between the initial and final peak positions (Δ2*θ*) was smaller at a high Ag content (Cu_0.5_Ag_0.5_; Δ2*θ* = 0.00124°) than at a low Ag content (Cu_0.9_Ag_0.1_; Δ2*θ* = 0.0045°), indicating more numerous CuO–Ag interfaces in the latter case. The catalysts were further analyzed over several cyclic voltammograms (+0.3 V to −0.4 V *vs.* RHE, 2 mV s^−1^, N_2_ atmosphere). After 4 CV cycles, all catalysts were fully reduced (Fig. S36†). Despite the observation of similar metallic Cu and Ag phases, differences in crystallite sizes and peak positions were observed between the samples. While the crystallite sizes of Cu (111), Cu (200), Ag (111), and Ag (220) increased with increasing Ag content, the positions of these reflections shifted to lower angles (Fig. S36 and S38†). The position shift of the Cu reflections can be explained by the expansion of the lattice parameter due to alloying with Ag. As bulk Cu and Ag are immiscible, only Cu–Ag nanoalloys can be formed.^34,74–77^

The CO electrochemical reduction, carried out at −0.4 V *vs.* RHE in a CO-purged electrolyte, only caused minor structural changes (Fig. S37 and 38†). During 20 min of CORR, the crystallite size and position of the reflections of the Cu and Ag phases did not change. Thus, we conclude that CO had a limited impact on the restructuring of the bulk structure under the applied conditions. Rietveld refinement was performed on the diffractograms recorded at half-time of the total CA duration. The fcc Cu and Ag lattice parameters were influenced by the composition of the *in situ* analyzed samples (Fig. S39, 40, Table S9 and Note S3†). The Cu lattice parameter increased by 0.0021 Å for Cu_0.9_Ag_0.1_ and by 0.0012 Å Cu_0.5_Ag_0.5_ compared to the Cu-only sample (3.6117 Å). Strehle *et al.* reported that the Cu lattice parameter of Cu–Ag nanoalloy systematically increased with the addition of Ag.^78^ As the Cu_0.9_Ag_0.1_ sample demonstrated the largest Cu lattice expansion, we speculate that the Cu phase hosted more Ag than the other samples. In line with the XPS and XAS results, this hypothesis also aligns with the finding that Cu_0.9_Ag_0.1_ contains many Cu–Ag interfacial sites, as observed in the Ag–Cu core–shell structure by STEM-EDX.

Rietveld refinement of the Ag phase showed a difference in unit cell parameters between as-prepared and reduced samples (Fig. S39 and Table S9†). Upon reduction, the decrease of the Ag unit cell size was more significant at low Ag content, indicating that more Ag interacted with CuO in the as-prepared samples. During reduction, the Ag lattice parameter in the Cu_0.9_Ag_0.1_ (4.0824 Å) and Cu_0.5_Ag_0.5_ (4.0822 Å) samples approached the one of Ag-only (4.0823 Å), while the lattice parameter of Cu_0.95_Ag_0.05_ was instead lower (4.0803 Å). This contraction could be due to the inclusion of Cu atoms in Ag domains near the Cu–Ag interface.^78,79^ These effects are more evident at low Ag content due to the low abundance of isolated Ag domains, in which Cu does not influence the Ag crystals. Previously, Jian *et al.* investigated how composition impacts the crystallographic and electronic properties of Cu–Ag solid solutions (nanoalloys).^79^ Replacing the Ag atoms with Cu resulted in lattice contraction. An Ag-rich solid solution displayed a lattice parameter close to pure Ag, while a Cu-rich sample exhibited a lattice parameter close to pure Cu. The Ag lattice contraction observed in Cu_0.95_Ag_0.05_ demonstrates that a significant amount of Cu is included in the Ag domains. Yet, the relatively small amount of such sites at 5 mol% Ag content did not improve the FE to C_2+_ products compared to the Cu-only sample. The lattice parameters of Cu and Ag in Cu_0.5_Ag_0.5_ resembled those of the corresponding pure metals, which indicates the abundance of separate Cu and Ag domains. While only minor differences in Ag unit cell parameters were found in Cu_0.9_Ag_0.1_, the Cu lattice expanded the most in this sample, hinting at high Ag substitution levels due to the formation of abundant Cu–Ag nanoalloys in agreement with the XPS and XAS results. Cu–Ag nanoalloys at the interface between the Cu-shell and the Ag-core led to enhanced C_2+_ product selectivity during CORR for the Cu_0.9_Ag_0.1_ sample.

### 2.5. The role of Cu–Ag interactions in CORR

Two main hypotheses concerning the nature of the active sites in bimetallic Cu–Ag catalysts for CORR to C_2+_ products have been discussed. The first postulates that partial oxidation of Cu surfaces enhances the C_2+_ product selectivity.^40,77^ The presence of Ag can stabilize Cu^*δ*+^ species at the Cu surface upon reduction. The higher binding strength of CO on Cu^+^ than on Cu is thought to increase the residence time of the reactant in the double layer, enhancing C_2+_ formation through CO–CO coupling. Surface analysis by quasi-*in situ* XPS of our samples revealed the prevalence of metallic Cu at the surface under reducing conditions. Moreover, structural and compositional analysis by *in situ* WAXS and *operando* XAS showed that oxidized Cu was absent during CORR. We also observed that Ag promoted the complete reduction of CuO to Cu. Therefore, we conclude that stabilizing oxidized Cu species by Ag cannot explain the improved product selectivity of the Cu_0.9_Ag_0.1_ sample. Alternatively, the promotional effect of Ag addition on C_2+_ formation during CORR has been linked to a higher density of interfacial Cu–Ag sites.^19,32,39,80^ The Cu–Ag interface forms a specific environment promoting the formation of Cu–Ag nanoalloys where electronic effects are concentrated. These affect the binding properties of reactants and intermediates, resulting in enhanced CO dimerization rates.^32,37–39,80^ Our EDX mapping revealed the abundance of core–shell structures in bimetallic samples with a low Ag content (Cu_0.9_Ag_0.1_), while Cu and Ag particles were separated at high Ag concentration (Cu_0.5_Ag_0.5_). Moreover, the different peak positions observed by *in situ* WAXS indicated the formation of Cu–Ag nanoalloys, which were more abundant at low Ag content and likely located at the interface of the Cu and Ag domains in the core–shell structures. Rietveld refinement confirmed that the presence of Ag influenced the Cu lattice parameter measured during reduction and CORR. The largest Cu lattice expansion was measured for Cu_0.9_Ag_0.1_ and caused by the abundance of the Cu–Ag nanoalloys. The amount of such Cu–Ag interfacial sites depended on the Ag content. At very low Ag content (≤5 mol%), the abundance of such nanoalloys is too low to improve the C_2+_ product selectivity. The larger amount of such sites at an Ag content of 10 mol% promoted the formation of C_2+_ products and suppressed HER. The high propanol faradaic efficiency (18% at −0.4 V *vs.* RHE) observed for the Cu_0.9_Ag_0.1_ sample likely originates from the abundant Cu–Ag nanoalloy sites, which facilitate not only C_1_–C_1_ but also C_2_–C_1_ coupling reactions. At high Ag content (50 mol%), the Cu and Ag phases are mostly separated, likely due to the predominant presence of separate CuO and Ag phases in the as-prepared sample and the low miscibility of Cu and Ag. We conclude that the physical separation of Cu and Ag phases is the primary cause of the selectivity shift from C_2+_ products to H_2_. Optimum Cu–Ag interactions were, therefore, obtained for the Cu_0.9_Ag_0.1_ sample.

Based on our WAXS data, it is reasonable to assume that the Cu–Ag interactions in the reduced catalysts strongly relate to the interactions between the CuO and Ag phases in the as-prepared samples formed during sol–gel synthesis. During sol–gel synthesis, the Cu and Ag atoms are homogeneously mixed at the atomic level in the gel.^81^ We expect that the calcination of the gel results in the segregation of Ag from CuO due to their different affinities with oxygen.^82^ Supported by XPS analysis, we contend that, at low Ag content, the segregated Ag atoms do not sinter into large particles, as these Ag species are highly dispersed in the solution. Instead, mixed oxides are formed at the CuO and Ag domains interface, likely stabilizing such small Ag particles. The presence of mixed oxides at the CuO–Ag interface indicates that the two phases are in close proximity. These mixed oxides are most likely the precursor for the Cu–Ag nanoalloys formed during CORR.^19^ Conversely, the sintering of Ag into large particles is likely at high Ag content^83^ which prevents the formation of mixed oxides at the interface with CuO. Moreover, EDX mapping before and after reduction pointed out some mobility of the Ag atoms despite remaining in the same oxidation state. Thus, although we believe that most of the Cu–Ag nanoalloy stems from the reduction of the mixed oxide in the interface region, the Cu–Ag nanoalloy may also originate from atom mobility during the reduction of the oxide precursors.^70^ Such Cu–Ag nanoalloys were, however, not formed during the reaction in Cu_0.5_Ag_0.5_, which contained primarily separate CuO and Ag particles after sol–gel synthesis. The low miscibility of Cu and Ag may explain why nanoalloys are not formed from isolated phases during CORR.^34,74,75^ Thus, we postulate that Cu–Ag nanoalloys, which benefit the C_2+_ product selectivity, evolved predominantly from the reduction of initially interacting CuO and Ag phases (mixed oxides) synthesized by the sol–gel method.

## 3 Conclusion

Bimetallic Cu–Ag catalysts of different compositions were prepared by sol–gel synthesis and compared to Cu-only and Ag-only samples in CORR. The composition strongly influences the C_2+_ product selectivity. While Cu_0.95_Ag_0.05_ did not improve the CORR performance compared to Cu-only, a Ag content of 10 mol% (Cu_0.9_Ag_0.1_) was optimal for forming C_2+_ products. A higher Ag content promoted H_2_ evolution, which competes with C–C bond formation. Cu_0.9_Ag_0.1_ was compared to a reference sample GE 0.9 made by galvanic exchange containing surface Cu–Ag nanoalloys. Their CORR performances were similar, indicating similar active sites in both samples under reaction conditions. The as-prepared samples were predominantly composed of crystalline CuO and Ag phases, as shown by *ex situ* WAXS and XAS measurements. Nevertheless, the presence of mixed oxides (*e.g.*, AgCuO_2_ or Ag_2_Cu_2_O_3_) at the interface between CuO and Ag was indicated by WAXS data and further supported by HAADF-STEM images combined with EDX mapping. Mixed oxides were present at the CuO–Ag interface, especially at low Ag content (≤10 mol%). Complementary XPS measurements revealed the partial oxidation of Ag species in these samples and a higher Ag dispersion than in samples containing more Ag, which confirms the abundance of CuO–Ag interfaces at low Ag content. Under electrochemically reducing conditions, these precursors underwent significant restructuring. Complete surface and bulk reduction were supported by quasi-*in situ* XPS, *in situ* WAXS, and *operando* XAS characterization. The presence of Ag promoted the complete reduction of CuO to Cu, as it was more challenging to reduce the Cu-only sample entirely. WAXS characterization showed the formation of Cu–Ag nanoalloys, next to crystalline Cu and Ag phases. The abundance of Cu–Ag nanoalloys was higher in Cu_0.9_Ag_0.1_ than in Cu_0.5_Ag_0.5_. Electron microscopy of used samples revealed that the Ag–Cu core–shell structures were abundant at low Ag content (Cu_0.9_Ag_0.1_) and scarce at high Ag content (Cu_0.5_Ag_0.5_), where mostly separated Cu and Ag particles were found. Therefore, the Cu–Ag nanoalloy phase observed in WAXS was likely located at the interface between the Ag-core and the Cu-shell of Cu_0.9_Ag_0.1_. The electronic effects present in the nanoalloys benefit C–C coupling. The presence of more Cu–Ag interfaces and the absence of separated Ag particles in Cu_0.9_Ag_0.1_ can explain the enhanced formation of C_2+_ products. Conversely, the presence of separate Cu and Ag particles with few interfaces in Cu_0.5_Ag_0.5_ is detrimental to the selectivity to C–C coupling reactions during CORR, resulting in a higher selectivity to H_2_. This study emphasizes the pivotal role of Cu–Ag nanoalloys in forming C–C bonds during CORR.

## Abbreviations

CORR: CO electrochemical reduction reaction
CO2RR: CO_2_ electrochemical reduction reaction
XPS: X-ray photoelectron spectroscopy
XAS: X-ray absorption spectroscopy
XANES: X-ray absorption near-edge spectroscopy
EXAFS: Extended X-ray absorption fine structure
STEM-EDX: Scanning transmission electron microscopy-Energy dispersive X-ray spectroscopy
HAADF-STEM: High-angle annular dark-field scanning transmission electron microscopy
ICP-OES: Inductively coupled plasma-optical emission spectroscopy
WAXS: Wide-angle X-ray scattering
OCP: Open circuit potential

## Data availability

The authors confirm that the data supporting the findings of this study are available within the article and its ESI.†

## Author contributions

Floriane A. Rollier (conceptualization, data curation, formal analysis, investigation, methodology, validation, visualization, writing the original draft), Valery Muravev (conceptualization, XAS and quasi *in situ* XPS, methodology, review), Nikolay Kosinov (XAS, methodology), Tim Wissink (XAS), Dimitra Anastasiadou (XAS), Bianca Ligt (WAXS), Laurent Barthe (methodology), Marta Costa Figueiredo (review & editing), Emiel J. M. Hensen (conceptualization, funding acquisition, review & editing).

## Conflicts of interest

There are no conflicts to declare.

## Supplementary Material

TA-013-D4TA04263H-s001
