# Supplementary material for: Cu–Ag interactions in bimetallic Cu–Ag catalysts enhance C2+ product formation during electrochemical CO reduction
Source: J Mater Chem A Mater. 2024 Dec 5;13(3):2285–300. doi: 10.1039/d4ta04263h (PMC11639664; doi:10.1039/d4ta04263h)
Supplement: TA-013-D4TA04263H-s001 [file TA-013-D4TA04263H-s001.pdf]

Supplementary Information (SI) for Journal of Materials Chemistry A.
This journal is © The Royal Society of Chemistry 2024

Supplementary Information

Cu-Ag interactions in bimetallic Cu-Ag catalysts enhance C_{2+} product formation during electrochemical CO reduction

Floriane A. Rollier [a], Valery Muravev [a], Nikolay Kosinov [a], Tim Wissink [a], Dimitra Anastasiadou [a], Bianca Ligt [a], Laurent Barthe [b], Marta Costa Figueiredo [a], Emiel J.M. Hensen [a]

[a] Laboratory of Inorganic Materials and Catalysis, Department of Chemical Engineering and Chemistry, Eindhoven University of Technology P.O. Box 513, 5600 MB Eindhoven, The Netherlands
[b] Synchrotron SOLEIL, L'Orme des Merisiers, Départementale 128, 91190 Saint-Aubin, France

Corresponding author: Emiel J.M. Hensen, e.j.m.hensen@tue.nl

Experimental details

Chemicals

$Cu(NO_3)_2.3H_2O$ 99% ACROS organics, $Ag(NO_3)$ ACS reagent >99.8%, and citric acid 99.5% were purchased from Merck Life Science. Commercial Cu nanoparticles 25 nm (TEM), purchased from Merck Life Science, were used to prepare galvanically exchanged samples. Nafion 5 wt% solution (Merck Life Science) and absolute ethanol (Biosolve) were used in the ink preparation. Carbon paper SIGRACET 22BB (Fuel Cell store) was used as the gas diffusion layer (GDL). Ultra-pure water was supplied in the laboratory by an ELGA device. Potassium hydroxide solution 47 wt% for analysis EMSURE was bought from Merck Life Science. Phenol 99% (Merck Life Science), DMSO 99% (Biolsove), and D_2O 99.9% (Merck Life Science) were used for preparing the internal standard solution for ^{1}H-NMR quantification.

Catalyst synthesis

A citrate-based sol-gel synthesis was employed for the preparation of the pure and bimetallic catalysts. A 0.2 M solution (100ml) containing the metal salts (Cu + Ag) was poured into a beaker. The solution was stirred and heated up to 80°C for an hour. Afterward, a 50 ml solution containing citric acid was added to the initial mixture. The ratio between citric acid and metal moles was kept at 1. The resulting light-blue solution is called "sol". The temperature of the solution was increased to 130°C and maintained for 6 h under stirring. After 6 h aging, the solution, called "gel", has still a light-blue color but has now a gel-like appearance. It is important to note the absence of precipitate in the solution. If precipitates are present, the synthesis was unsuccessful. The obtained gel was then dried overnight in an oven at the same temperature. After crushing the dried gel, the powder was calcined at 450°C for 3 h (ramp: 5°C.min^{-1}) to decompose the organics and nitrate residues. A black powder was obtained for all samples containing Cu. The pure Ag sample was shiny white grey.

The GE 0.9 sample was prepared by galvanic exchange. 200 mg of commercial Cu nanoparticles were immersed in a 300 µM solution of $AgNO_3$ (100 ml) for 1 h while stirring. The obtained catalyst was then recovered by centrifugation and rinsed three times with ultra-pure water. The catalyst was then dried at 30°C

overnight under vacuum. As galvanic exchange occurs at the surface, the Ag/Cu ratio of the GE 0.9 material was similar to the surface element ratio of the used $Cu_{0.9}Ag_{0.1}$ measured by quasi-in situ XPS (**Table S6**).

Inductively coupled plasma-optical emission spectrometry (ICP-OES)

Catalyst compositions were analyzed by ICP-OES using an AMETEK Spectroblue spectrometer. The instrument is equipped with an axial plasma viewing and a free running 27.12 MHz generator (1400 W). The as-synthetized catalysts (25 mg) were dissolved in 5 ml of concentrated nitric acid (65%). The solution was stirred and heated to 60°C for 1 h until the catalyst was completely dissolved. After cooling down, the solution was diluted to a total volume of 50 ml. The obtained solution was diluted again 50 times. Each sample was duplicated to verify the accuracy of the measurements. Calibration solutions were prepared using Cu and Ag standards of known concentrations.

Ex situ characterization

Wide-angle X-ray scattering (WAXS) was employed to characterize the phases of the as-prepared samples, their sizes, and crystallinity. The data was recorded at the beamline ID 31 of the European Synchrotron Radiation Facility (ESRF) in Grenoble, France. An excitation energy of 75 keV (λ = 0.0165312 nm) and a Pilatus CdTe 2M detector were used to probe the material in a Debye-Scherrer configuration. Powders were placed in Kapton capillaries and measured at a fixed sample-detector distance.
The transition from reciprocal space (q) and real space (2θ) was carried out using the following formula: $|q| = \left(4 * \frac{\pi}{\lambda}\right) * \sin\left(\frac{2\theta}{2}\right)$. Bragg's law, nλ = 2d*sinθ, was employed to assign the reflections based on existing literature. Reflections were fitted using a Voigt function to obtain useful parameters such as position, FWHM, and area. Rietveld refinement was carried out using the software G-SAS II. The refinement was limited to histogram scale factor, sample displacement, phase fraction, crystallite size, and unit cell parameters.

X-ray photoelectron spectroscopy (XPS) was used to determine the surface chemical state and the surface composition of the as-prepared samples. The characterization of all the samples was carried out using a lab-based SPECS NAP-XPS spectrometer (analyzer SPECS Phoibos 150 NAP). A monochromatic Al Kα source (1486.6 eV) was used during the experiments. Spectra were analyzed using the CasaXPS software. The C-C component of the C 1s spectral line was used for energy correction (284.5 eV).

Synchrotron-based X-ray photoelectron spectroscopy was employed to establish the surface speciation of the as-prepared materials at various depths. The data was recorded at the HIPPIE beamline of the MAX IV synchrotron radiation facility in Lund, Sweden. The acquisition of spectra was done using a Scienta Hipp-3 equipped with a SWIFT acceleration mode. By tuning the excitation energy (hv) of the incident beam, several depths were probed. Cu $2p_{3/2}$ and Ag 3d spectral lines were measured at the same kinetic energy, which implies that the same depth was analyzed. **Table S1** displays the excitation and kinetic energies used in this study.

Table S1. Excitation energies used to probe several kinetic energies / depths.

Kinetic energy (eV)	hv for Cu (eV)	hv for Ag (eV)
**220**	1150	590
**570**	1500	940
**870**	1800	1240

As CuO and AgO_x species were sensitive to beam damage, the Cu $2p_{3/2}$ and Ag 3d XPS lines were recorded on a new spot every time. Spectral calibration was done using internal standard lines. The center of the Cu^{2+}

satellite in the Cu $2p_{3/2}$ lines (942.2 eV) was used to correct the Cu $2p_{3/2}$ spectra. The Ag correction was made on Ag° contribution of the Ag 3d line (368.2 eV).

X-ray absorption spectroscopy (XAS) was used to probe the bulk oxidation state (X-ray absorption near edge spectroscopy, XANES) and the local environment (Extended X-ray absorption fine structure, EXAFS) of the as-prepared samples. The experiments were carried out at the ROCK beamline of the SOLEIL synchrotron radiation facility, Paris, France. Catalyst powders were deposited on carbon paper, as explained in the "Electrode preparation" section, and measured in transmission and fluorescence mode. The fluorescence detector was placed at a 45° angle with respect to the sample. Data was recorded at the Cu K-edge (8 979 eV) and the Ag K-edge (25 514 eV). These energies were used for the energy calibration of the first derivative. The spectra were background-subtracted and normalized before fitting using the software FASTOSH (Developed on the SAMBA beamline, SOLEIL). Linear combination fitting was carried out using the software LARCH. Reference materials were used as input parameters: CuO (synthesized by sol-gel method), Cu foil, Cu (resulting from the full reduction of CuO), commercial Cu_2O, and $Cu(OH)_2$ (synthesized by precipitation). Similarly to the samples, the powder references were deposited on carbon paper at a loading of 5 $mg.cm^{-2}$ and were measured in transmission and fluorescence mode. Spectra were acquired for 10 min on each sample and were averaged to increase the signal-to-noise ratio. A k^3-weighed Cu K-edge and Ag K-edge parameter were used for EXAFS analysis. A k range of 3 to 12 Å was used for Cu and Ag K-edges. EXAFS data treatment was done with the Artemis software. Reference crystal structures of CuO, Cu, and Ag were used to calculate the scattering paths for fitting the data of the references and Cu-Ag bimetallic samples. An amplitude reduction factor S_0^2 of 0.88 was determined by fitting the Cu-Cu single scattering path of the first shell of Cu foil, using a coordination number of 12. An amplitude reduction factor S_0^2 of 0.79 was calculated by fitting the Ag-Ag single scattering path of the first shell of Ag foil, using a coordination number of 12. These S_0^2 values were used to fit the FT-EXAFS of the Cu-Ag bimetallic catalysts and obtain the coordination numbers of the Cu and Ag atoms and the atomic distances.

Bright-field transmission electron microscopy (TEM) *and* ***high-angle annular dark-field scanning transmission electron microscopy (HAADF-STEM)*** were employed to image the as-prepared and used samples. The as-prepared samples were dispersed in absolute ethanol before deposition on Mo-grids. After 1 h CORR at -0.4 V vs RHE, the used catalysts were recovered from the GDE by lightly scratching its surface with a spatula and dispersing the collected powder in absolute ethanol before deposition on Mo-grids. A FEI cubed titan Cs-corrected 80-300kV transmission electron microscopy operating at 300 kV was used. ***Energy-dispersive X-ray spectroscopy (EDX)*** elemental analysis was acquired through HAADF-STEM mode.

Electrode preparation

After synthesis, the prepared materials were crushed to obtain a fine powder. An ink containing 25 mg of catalyst, 300 µl of Nafion solution 5 wt%, and 500 µl of absolute ethanol was mixed and sonicated for 20 min. The sonication step allows the dispersion of the nanopowder in the ethanol solvent. The ink was then drop casted on a 5 cm^2 commercial carbon paper (Sigracet 22BB) and dried in air overnight. The catalyst loading was 5 $mg.cm^{-2}$. A mixture of water (20%v) and isopropanol (80%v) was used to prepare the pure Ag ink as no optimum dispersion could be obtained with ethanol.

Electrochemical measurements

A custom-made H-cell was used for all electrochemical characterization and catalytic tests. To decrease the mass transport limitations, a gas diffusion electrode (GDE) configuration was used. The catalyst deposited on a GDL, constituting the GDE, was used as cathode. A platinum foil and a mini-RHE (Gaskatel) were used as counter and reference electrodes, respectively. Cathode and anode compartments were separated by an anion exchange membrane (Fumasep FAA-3-PK-130, Fumatech BWT). Each compartment was filled with 23 ml of a 3M KOH electrolyte.

Cyclic voltammetry (CV) was used to probe the reduction and oxidation events of the materials. The electrolyte was flushed with He (15 ml.min^{-1}) for 15 min prior to CV recording. To characterize an electrode before CORR, a short potential range from +0.5 V vs RHE to -1.0 V vs RHE (start at +0.2 V vs RHE) and a scan rate of 50 mV.s^{-1} were used. On a pristine sample, two cycles were first recorded to ensure the correct reading of the reference electrode. The *OH/*O species adsorption from +0.35 V vs RHE to +0.5 V vs RHE indicated a well-functioning reference. After recording the electrochemical impedance spectroscopy, 10 other CV cycles were recorded, and iR correction was applied.

Electrochemical Impedance Spectroscopy (EIS) was employed to determine the iR drop correction to be applied to the potential. A potential of -0.2 V vs RHE, located in the double layer, was chosen to measure EIS. Frequencies ranging from 50 kHz to 0.5 Hz were used, and 80% of the measured iR drop was applied to correct the potential of the CV, ECSA, and CA measurements.

Electrochemically active surface area measurements by the double-layer capacitance method (ECSA) were used to measure the surface area of the materials. To ensure the absence of oxygen, the electrolyte was flushed with He (15 ml.min^{-1}) for 15 min prior to starting the measurement. A potential range from -0.1 V vs RHE to -0.2 V vs RHE, located in the double layer, was chosen. Several CVs using 10, 20, 50, 100, and 150 mV.s^{-1} scan rates were recorded. At -0.15 V vs RHE, the average of the current from the cathodic and anodic sweeps was calculated and plotted against the scan rates. The capacitance of the layer (Cdl) or roughness factor (RF) was given by the slope of this plot. The following formula describes the ECSA calculation:

$$ECSA = \frac{Cdl}{Cref}$$

A value of 40 µF.cm^{-2} for C*ref* was obtained from the literature[1,2].

Catalytic performance – CORR

The catalytic CORR performance of the samples was evaluated in a leak-tight H-cell. The working electrode was composed of carbon paper covered with the catalyst (GDE). A Pt foil and a mini-RHE were used as counter and reference electrodes, respectively. The working and reference electrodes were separated by a fixed distance of 2 mm. The geometrical surface areas of the working and counter electrodes were equal to 1 cm^2. The CORR catalytic performance tests are run after characterization of the electrode by CV (start: +0.2 V vs RHE, from +0.5 V to -1.0 V vs RHE, 50mV.s^{-1}, 10 cycles), EIS measurements as described previously and ECSA measurements as described above.

The 3 M KOH electrolyte was flushed continuously by 15 ml.min^{-1} of CO through the GDE. The unreacted CO and the gaseous products were analyzed online by a mass spectrometer (Balzers Thermostar GSD 300 T2, Pfeiffer vacuum) and a gas chromatography apparatus (GC TRACE 1300, Thermo-Fischer Scientific). During operation, a flow meter (Bronkhorst) monitored the outlet flow of the cell, and a manometer measured

the pressure in the cell. The 23 ml of electrolyte placed in each compartment were static. Liquid products were analyzed by ^{1}H-NMR after the reaction.

A FID and a TCD detector were used for the detection of hydrocarbons and permanent gases (H_2 and CO), respectively. The separation of permanent gases was carried on a Hayesep Q pre-column and a Shin-Carbon ST column. Hydrocarbon separation was carried out on an Al_2O_3/KCl column. After starting the chronoamperometry measurements (1h at -0.4 V, -0.5 V, or -0.6 V vs RHE), the gas outlet was injected in the GC every 8 min. The constant production of gases was monitored by the mass spectrometer while the elution took place in the GC. The ^{1}H-NMR spectra were recorded on a (Bruker) 400 MHz spectrometer. A measurement sequence including 400 scans and a delay time (d1) of 15 s was used. The delay time of 15 s was chosen to ensure the full spin relaxation required by the analytes. The NMR sample was prepared by mixing 630 µl of electrolyte and 70 µl of internal standard solution. The internal standard solution contained 10 mM of DMSO and 50 mM of Phenol in D_2O. The following equation was used to calculate the concentration of the analytes:

$$C_a = \frac{I_a}{I_{IS}} * \frac{N_{IS}}{N_a} * C_{IS}$$

With C_a the concentration of analyte (M), I_a the integral area of the -CH3 (for acetate, ethanol, propanol) or -CH (for formate) signal, N_a the number of protons involved in the signal (3H for $-CH_3$, 1H for -CH), C_{IS} the known concentration of internal standard, I_{IS} the integral area of the internal standard signal, and N_{IS} the number of protons involved in the internal standard signal (6H for DMSO and 2H for the protons in meta position of the phenol).

A fresh electrode was used for every single measurement. The Faradaic efficiencies were calculated using the following formula:

$$\text{FE (\%)} = \frac{(ne- * n\,products * F)}{Q}$$

With n_{e-} the number of exchanged electrons to produce one mol of product, $n_{product}$ the number of mol of the product calculated from GC or NMR data, *F* the Faraday constant (96485 $C.mol^{-1}$), *Q* the charge spent over the considered unit of time (per minute or hour).

After normalization of the total geometrical current densities by the electrochemically active surface area (giving the ECSA-normalized total current density, $J_{ECSA\ total}$), the ECSA-normalized partial current densities ($J_{ECSA\ partial}$) were calculated with the formula:

$$J_{ECSA\ partial} = FE_{product} * J_{ECSA\ total}$$

The average and the standard errors of three measurements were calculated.

In situ and operando characterization

Quasi-in situ XPS was employed to evaluate the surface speciation of the samples after reduction. A SPECS electrochemical cell was attached to a lab-based SPECS NAP-XPS spectrometer (analyzer SPECS Phoibos 150 NAP) allowing the transfer of reduced samples from the reaction to the UHV environment without air exposure. A GDE covered with a catalyst, a Pt foil, and a mini-RHE were used as working, counter, and reference electrodes, respectively. The one-compartment cell was flushed with He from the installation of the electrode until its transfer to the UHV chamber. A 3 M KOH electrolyte was used and flushed continuously with He during the experiment. Before any electrochemical measurement, the electrolyte was flushed for at least 20 min. The electrochemical measurement sequence consisted of 10 CVs from +0.5 V to -1.0 V vs RHE followed by a chronoamperometry at -0.4 V vs RHE for 10 min. Under He flow, the electrolyte

was then removed from the cell. The electrode and cell were washed 6 times with Ar-purged ultra-pure water to ensure the removal of KOH at the surface of the electrode and the absence of air. After 20 min of drying under He flow, the electrode was transferred to the UHV chamber. Next, the sample was transferred into the analysis chamber with a base pressure below 10^{-9} mbar. A monochromatic Al kα source (1486.6 eV) was used for the XPS measurements. Spectra were treated using the CasaXPS software. The C-C component of the C 1s spectral line was used for energy correction (284.5 eV). Cu 2p, Cu LMM, Ag 3d, Ag MNN, O 1s, C 1s, and survey spectra were recorded for each sample. The fitting parameters related to Cu and Ag species were taken from the literature[3–6]. First, a Shirley background was subtracted. The Cu $2p_{3/2}$ was fitted using a GL (30) function for CuO and $Cu(OH)_2$ components while GL (80) was used for $Cu^{+/0}$. The Ag 3d components were fitted using a GL (80) function. The Cu LMM and Ag MNN were analyzed based on the maximum kinetic energy.

In situ WAXS was utilized to characterize the crystalline phase transformation upon electrochemical reduction and CORR. The measurements were carried out at the beamline ID31 of the ESRF synchrotron radiation facility. An incident photon energy of 75 keV (0.0165312 nm) was used. A homemade electrochemical cell was placed between the beam entry and a Pilatus CdTe 2M detector. The measurements were carried out using a Debye-Scherrer configuration. The catalyst was deposited on a carbon paper electrode as previously described (loading 5 $mg.cm^{-2}$) and served as working electrode. A Pt wire and a mini-RHE were used as counter and reference electrodes, respectively. PEEK windows of 250 µm thickness were used to minimize X-ray absorption. A 3 M KOH electrolyte was flown through the cell during the whole duration of the experiment. First, the electrolyte was flushed with N_2 for 20 min before any electrochemical measurement. Under continuous N_2 flow, a first CV cycle was recorded between +0.5 V and -0.3 V vs RHE using a scan rate of 2 $mV.s^{-1,}$ and 3 subsequent CV cycles were recorded between +0.5 V and -0.4 V vs RHE using a scan rate of 2 $mV.s^{-1}$. The electrolyte was then flushed with CO for 10 min before CORR experiments were started. Under continuous CO flow, a chronoamperometry at -0.4 V vs RHE was recorded for 20 min. The WAXS signal was recorded during the CORR measurement. A diffractogram was measured in 0.5 s. During data treatment, the average of 10 diffractograms covered a potential range of 20 mV for the CV cycles. The average of 60 diffractograms covered a duration of 1 min during the CA at -0.4 V vs RHE in CO. A background subtraction was then applied to the diffractograms accounting for carbon and electrolyte contributions. The measurements recorded in reciprocal space q were converted to real space 2θ data using $|q| = \left(4 * \frac{\pi}{\lambda}\right) * \sin\left(\frac{2\theta}{2}\right)$. For peak assignment, the reflection positions were converted using conventional Cu Kα wavelength and Bragg's law (nλ = 2d*sinθ). The reflections were fitted using a Voigt function. Rietveld refinement was carried out using the software G-SAS II. The refinement was limited to histogram scale factor, sample displacement, phase fraction, size, and unit cell parameters.

Operando XAS was employed to measure the oxidation state evolution and the in situ formed local environment during CORR. The measurements were carried out at the beamline ROCK of the SOLEIL synchrotron radiation facility[7,8]. The grazing incidence of the mirrors was 2.5 mrad for harmonic rejection. At Cu K-edge, we utilized a quick-monochromator Si (111) combined with the B_4C-coated mirrors[8]. At Ag K-edge, we utilized a quick-monochromator Si (220) combined with the Pt-coated mirrors[8]. Additionally, CVD-250 µm filters were used to limit heat-bumps of the monochromators, which improves the energy resolution and increases the photon flux. The spectro-electrochemical cell used in this study was designed and built by the beamline engineer. The H-cell has a flow-by configuration where the working and counter electrodes are

separated by an anion-exchange membrane (Fumasep FAA-3-PK-130). The catalyst was deposited on a carbon paper electrode as previously described (loading 5 mg.cm^{-2}) and served as working electrode. A Pt mesh and a mini-RHE were used as counter and reference electrodes, respectively. The cell was placed at a 45° angle with respect to the beam entry and the fluorescence detector. A 3 M KOH electrolyte was used in all experiments. N_2 or CO was flushed in the gas compartment located behind the working electrolyte and closed by a Kapton window. The outlet of this compartment was connected to a mass spectrometer (MKS Cirrus, LM99 analyzer) for qualitative analysis of the reaction products. Before starting the experiments, the cell was flushed with 15 ml.min^{-1} of N_2 for 20 min. During this time, the XAS signal of the fresh catalyst in the cell was recorded. Under continuous N_2 flow, 3 CV cycles from +0.5 V to -0.6 V vs RHE were recorded using a scan rate of 5 mV.s^{-1}. The Cu K-edge (8 979 eV) spectra were measured simultaneously with a rate of 2 spectra per sec. After the CV cycles, Ag K-edge (25 514 eV) spectra were measured at OCP for 15 min. The cell was then flushed with 15 ml.min^{-1} of CO for 10 min. Under continuous CO flow, a chronoamperometry at -0.4 V vs RHE was measured. Meanwhile, the XAS signal of Cu K-edge was analyzed during the first 20 min before switching to Ag K-edge for 10 additional minutes. Subsequently, a chronoamperometry at -0.6 V vs RHE was measured, following the same XAS sampling pattern. The data was recorded at the Cu K-edge (8 979 eV) and the Ag K-edge (25 514 eV). These energies were used for the energy calibration of the first derivative. The spectra were background-subtracted and normalized before fitting using the beamline data extraction program and the software FASTOSH (Developed on SAMBA beamline, SOLEIL)[9]. The spectra were averaged by sets of 10 covering 25 mV for the CV measurements and 5 sec for the chronoamperometries. Linear combination fitting of the XANES spectra was carried out using the LARCH software[10]. Reference materials were deposited on carbon paper with a loading of 5 mg.cm^{-2} to match the sample conditions: CuO (synthesized by sol-gel method), Cu (resulting from the complete reduction of CuO), commercial Cu_2O and $Cu(OH)_2$ (synthesized by precipitation). They were used as input parameters for the linear combination analysis. The difference of magnitude between the adsorption edges of samples measured in transmission and fluorescence mode was lower than 0.1, indicating that self-absorption is minor in these measurements. A k^3-weighed Cu K-edge and Ag K-edge parameter were used for FT-EXAFS analysis. A k range of 3 to 12 Å was used for Cu K-edge and Ag K-edge. The FT-EXAFS data treatment was carried out using the software Artemis as described before. During XPS, WAXS, and XAS experiments, no iR compensation was applied to the potential.

Table S2. Compositions of the as-synthesized Cu-Ag bimetallic catalysts measured by Inductively Coupled Plasma Optical Emission Spectroscopy (ICP-OES).

Sample	Cu molar fraction	Ag molar fraction	Standard deviation on two measurements
$Cu_{0.99}Ag_{0.01}$	0.98993	0.01007	±0.00001
$Cu_{0.95}Ag_{0.05}$	0.95098	0.04902	±0.00009
$Cu_{0.9}Ag_{0.1}$	0.90065	0.09935	±0.00035
$Cu_{0.75}Ag_{0.25}$	0.75642	0.24358	±0.00010
$Cu_{0.5}Ag_{0.5}$	0.51376	0.48624	±0.00010

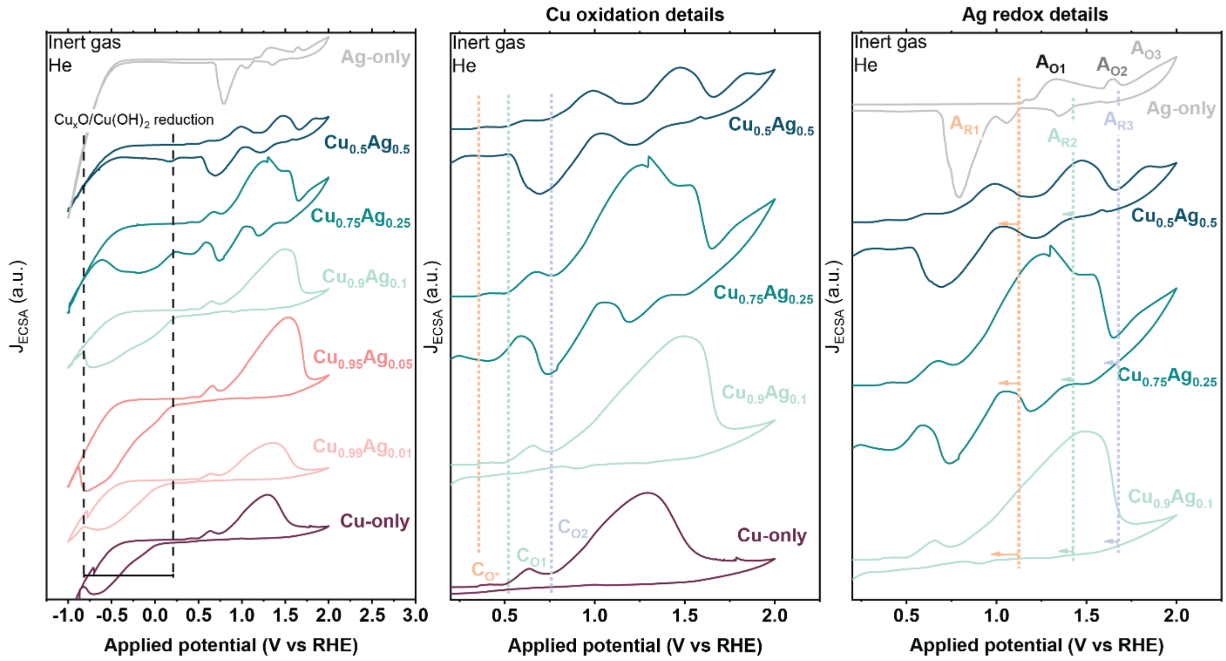

Figure S1. Cyclic voltammograms of the Cu-Ag bimetallic samples with He-purged electrolyte, 3 M KOH, from +2.0 V to -1.0 V vs RHE, 50 mV.s^{-1}. The electrolyte was purged with 15 ml.min^{-1} of He for 15 min before the CV were recorded.

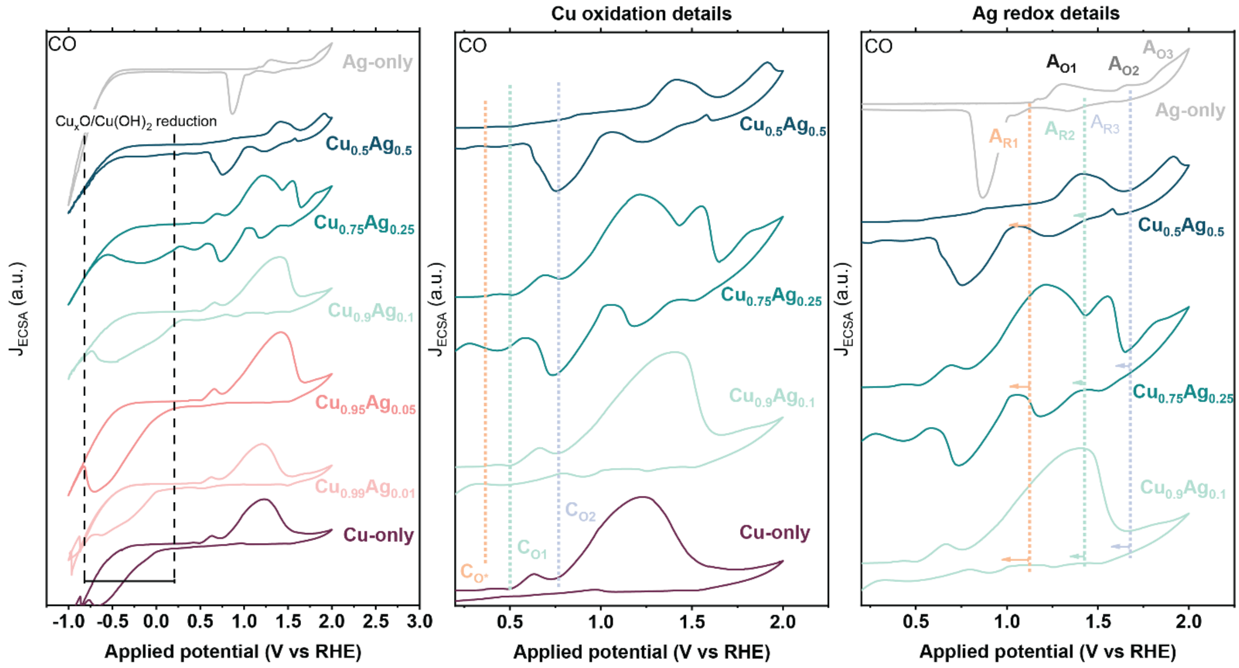

Figure S2. Cyclic voltammograms of the Cu-Ag bimetallic samples with CO-purged electrolyte, 3 M KOH, from +2.0 V to -1.0 V vs RHE, 50 mV.s^{-1}. The electrolyte was purged with 15 ml.min^{-1} of CO for 15 min before the CV were recorded.

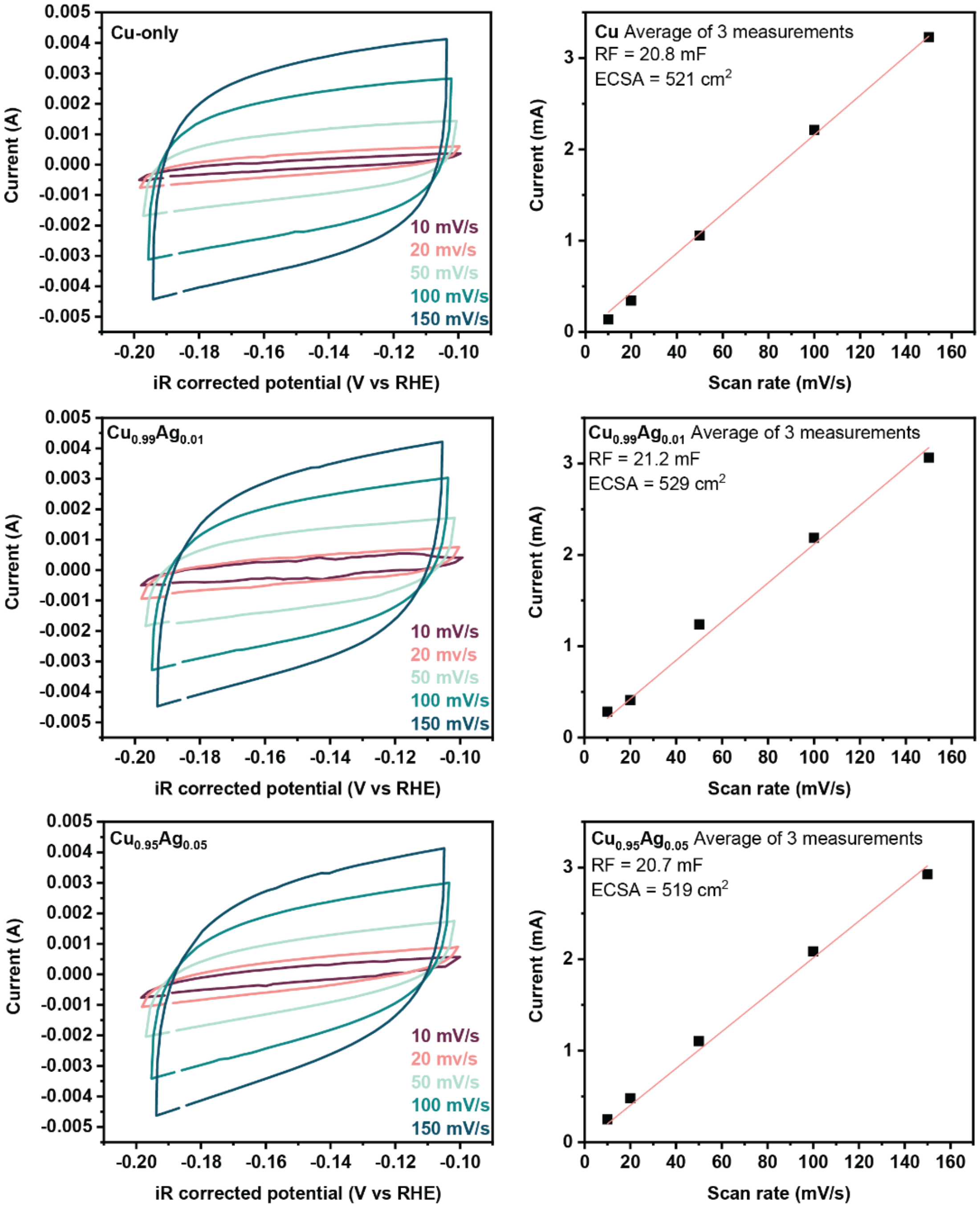

Figure S3. ECSA measurements of Cu-only, $Cu_{0.99}Ag_{0.01}$ and $Cu_{0.95}Ag_{0.05}$.

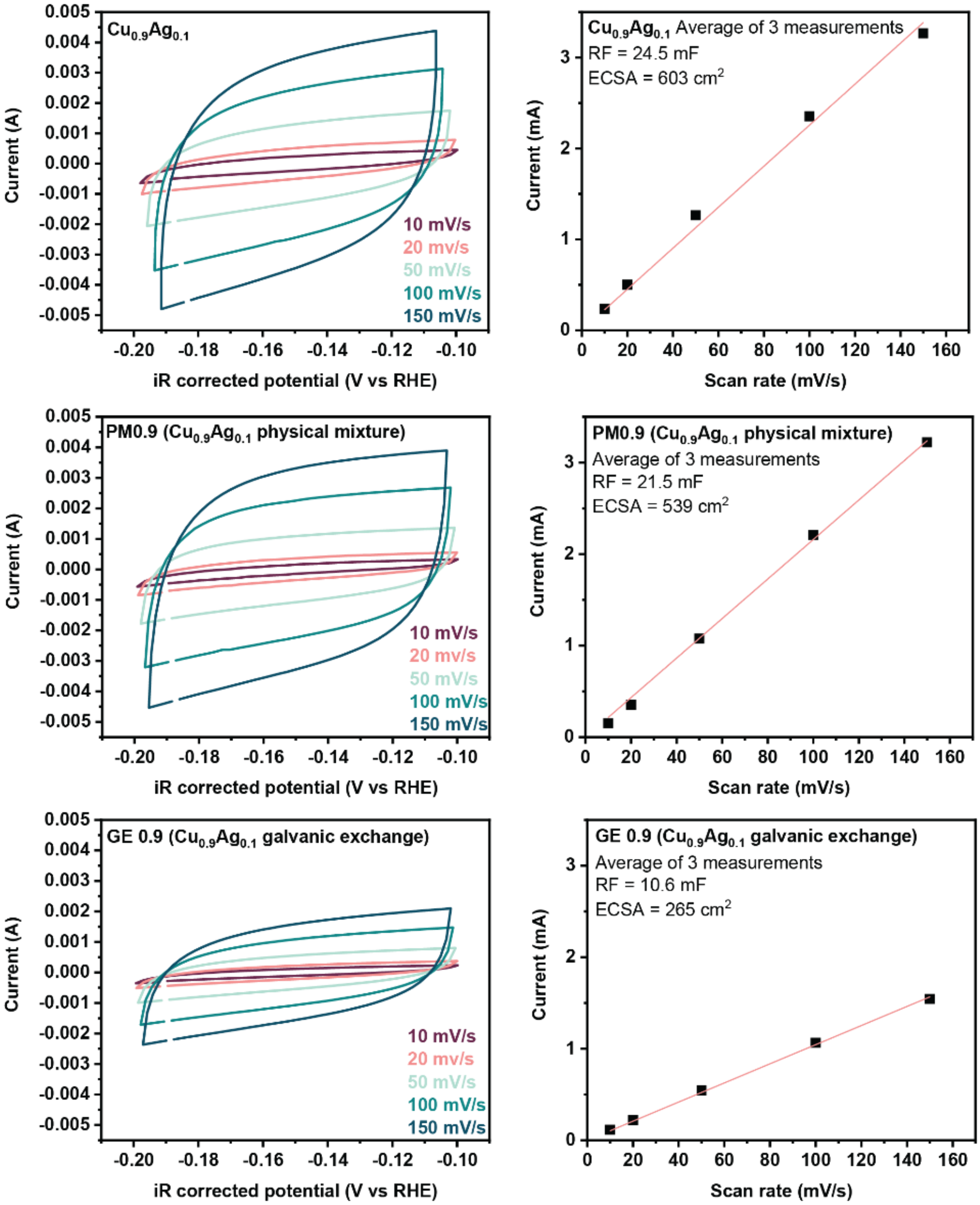

Figure S4. ECSA measurements of $Cu_{0.9}Ag_{0.1}$, physical mixture $Cu_{0.9}Ag_{0.1}$ and galvanic exchange $Cu_{0.9}Ag_{0.1}$.

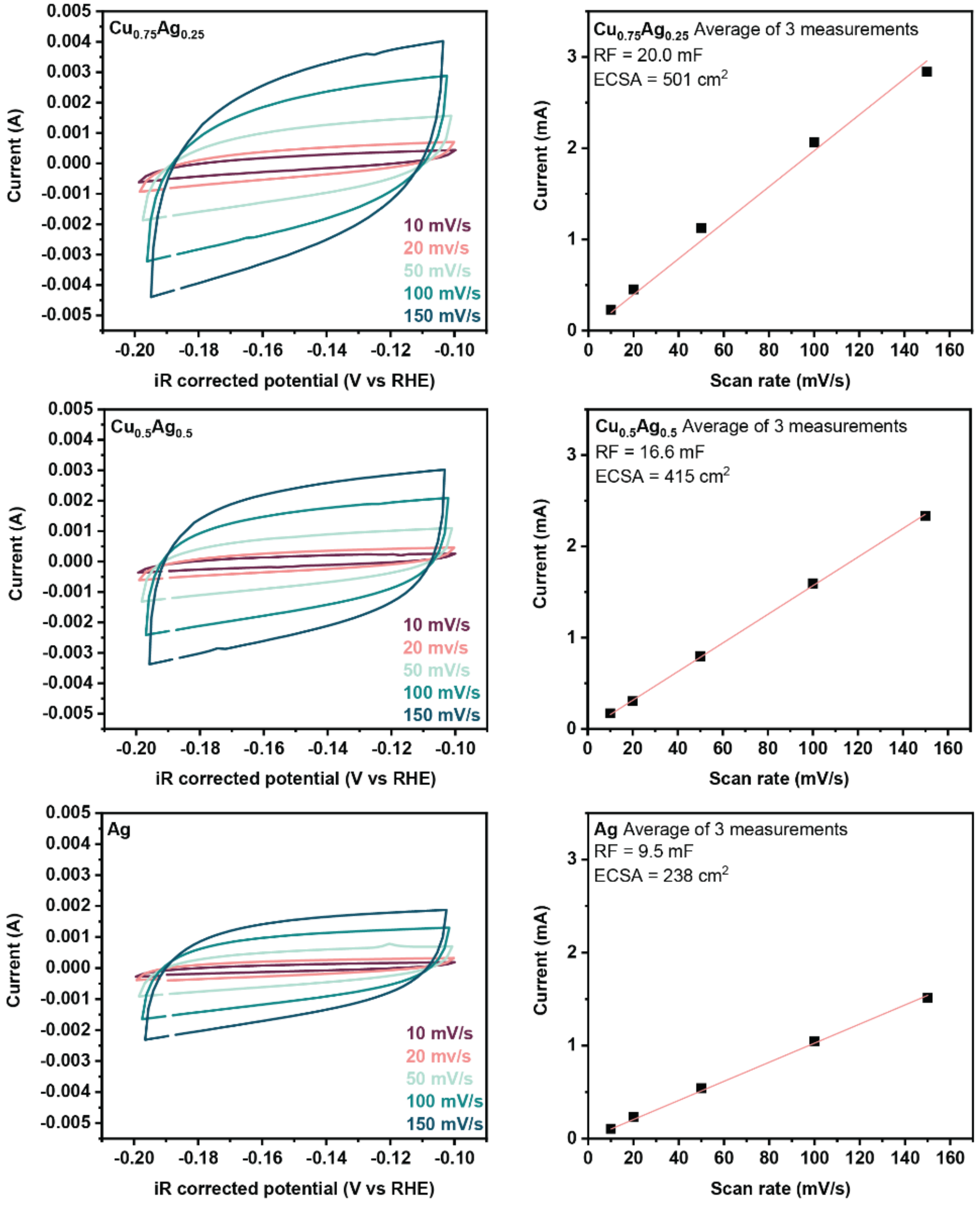

Figure S5. ECSA measurements of $Cu_{0.75}Ag_{0.25}$, $Cu_{0.5}Ag_{0.5}$ and Ag.

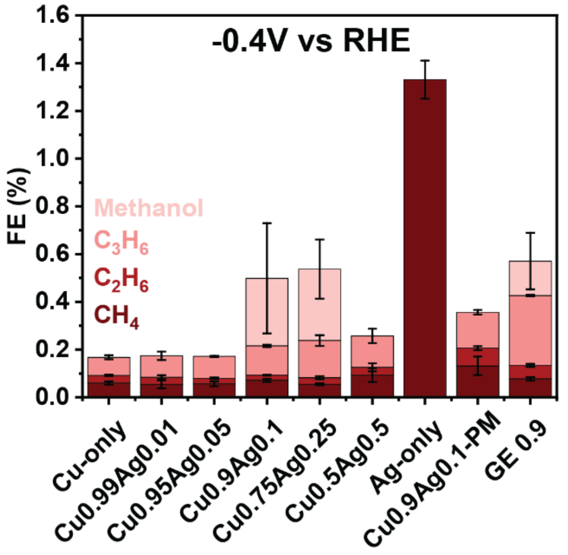

Figure S6. Faradaic efficiencies towards the minor products during CORR at -0.4 V vs RHE, 1 h, 3 M KOH.

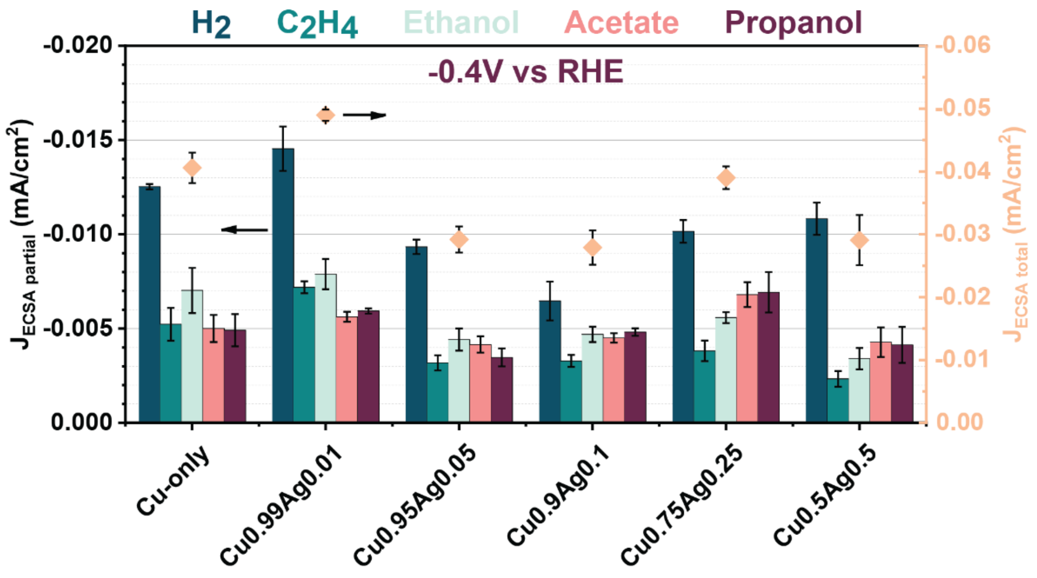

Figure S7. ECSA-normalized current densities measured during CORR at -0.4 V vs RHE, 1 h, 3 M KOH.

Note S1.

The ECSA-normalized total current densities ($J_{ECSA\ total}$), recorded at -0.4 V vs RHE, was between 27 and 50 μA.cm^{-2} (**Fig. S7**). Similar to the selectivity trend, ECSA-normalized partial current densities were composition-dependent at this potential. The largest current densities towards hydrogen ($J_{ECSA\ H2}$ > 12.5 μA.cm^{-2}) were measured on Cu and $Cu_{0.99}Ag_{0.01}$. The $J_{ECSA\ H2}$ was significantly decreased to less than 10 μA.cm^{-2} on samples containing between 5 mol% and 10 mol% of Ag. A greater Ag content had a negative impact on the suppression of HER and activities higher than 10 μA.cm^{-2} were observed. The current densities to C_{2+} products followed a similar trend. Samples showing a high $J_{ECSA\ total}$ (Cu and $Cu_{0.99}Ag_{0.01}$) displayed high ECSA-normalized partial current densities to all products, including C_{2+} products. However, this high activity led to low selectivity to C_{2+} products. Conversely, $Cu_{0.95}Ag_{0.05}$ and $Cu_{0.9}Ag_{0.1}$ showed overall low total activity, but high C_{2+} selectivity. The $J_{ECSA\ propanol}$ on Cu and $Cu_{0.9}Ag_{0.1}$ reached a similar value of 5 μA.cm^{-2}. With the lowest H_2 activity, $Cu_{0.9}Ag_{0.1}$ was the most efficient catalyst for C_{2+} formation despite the lower overall current density.

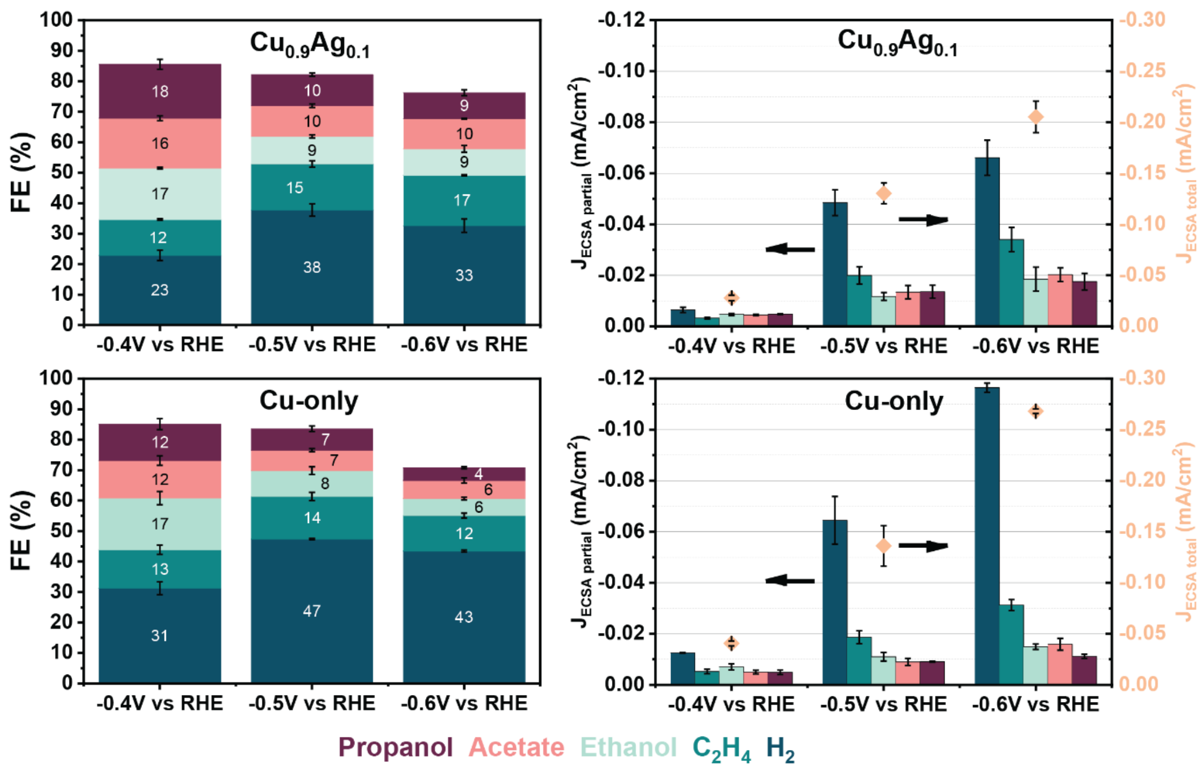

Figure S8. Faradaic efficiencies and ECSA-normalized current densities produced by $Cu_{0.9}Ag_{0.1}$ and Cu-only during CORR at -0.4 V, -0.5 V, and -0.6 V vs RHE, 1h, 3 M KOH.

Table S3. Faradaic efficiencies produced by $Cu_{0.9}Ag_{0.1}$ and Cu-only during CORR at -0.4 V and -0.6 V vs RHE, 1h, 3 M KOH.

**CORR at -0.4V vs RHE**	**Cu-only**	**$Cu_{0.9}Ag_{0.1}$**	Δ
H_2	31.2 ± 2.1 %	22.8 ± 1.7 %	- 8.4 % for $Cu_{0.9}Ag_{0.1}$
Ethylene	12.6 ± 1.5 %	11.8 ± 0.2 %	0.8 % (within error bars)
Ethanol	17.0 ± 2.2 %	16.9 ± 0.3 %	0.1 % (within error bars)
Acetate	12.3 ± 1.6 %	16.4 ± 0.8 %	+ 4.1 % for $Cu_{0.9}Ag_{0.1}$
Propanol	12.0 ± 1.8 %	17.7 ± 1.6 %	+ 5.7 % for $Cu_{0.9}Ag_{0.1}$
**CORR at -0.6V vs RHE**	**Cu-only**	**$Cu_{0.9}Ag_{0.1}$**	Δ
H_2	43.4 ± 0.3 %	32.6 ± 2.2 %	- 10.8 % for $Cu_{0.9}Ag_{0.1}$
Ethylene	11.7 ± 0.8 %	16.5 ± 0.2 %	+ 4.8 % for $Cu_{0.9}Ag_{0.1}$
Ethanol	5.6 ± 0.4 %	8.7 ± 1.1 %	+ 3.1 % for $Cu_{0.9}Ag_{0.1}$
Acetate	5.9 ± 0.9 %	9.9 ± 0.2 %	+ 4.0 % for $Cu_{0.9}Ag_{0.1}$
Propanol	4.2 ± 0.3 %	8.5 ± 1.0 %	+ 4.3 % for $Cu_{0.9}Ag_{0.1}$

Note S2.

The total faradaic efficiency did not reach 100% at all potentials. At a material level, the dissolution-redeposition processes consumed likely part of the missing charge, as the $Cu^{+/2+}$ ions in solution were reduced to Cu, using each 1 or 2 electrons[11]. At a process level, the use of an anion exchange membrane for the transfer of OH^- allowed the exchange of larger charged molecules such as formate or acetate but also neutral molecules such as ethanol and propanol. These products could be readily oxidized when reaching the anode.

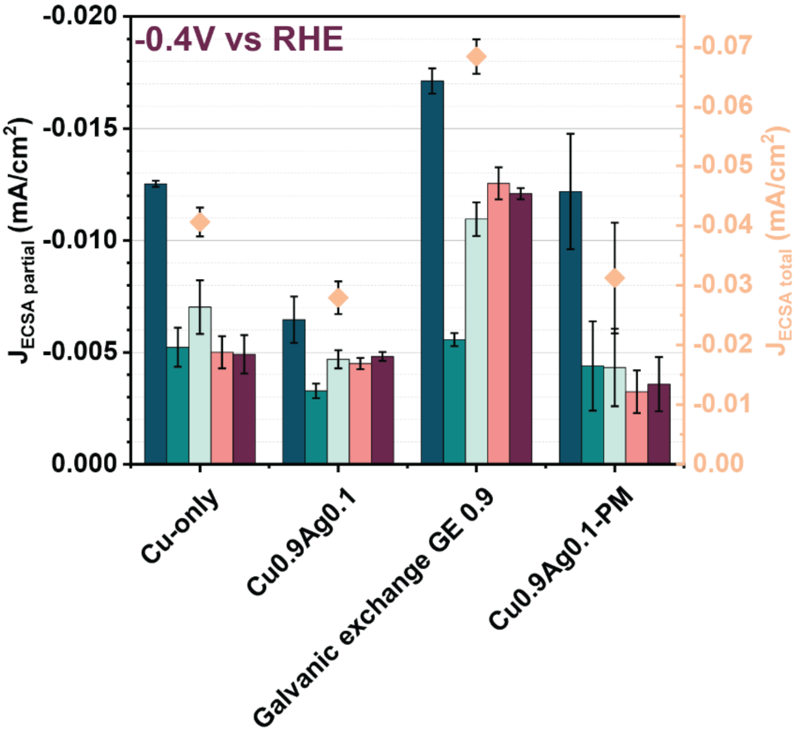

Figure S9. ECSA-normalized current densities of $Cu_{0.9}Ag_{0.1}$ compared to $Cu_{0.9}Ag_{0.1}$-PM by physical mixture and GE 0.9 galvanic exchange during CORR at -0.4 V vs RHE, 1 h, 3 M KOH.

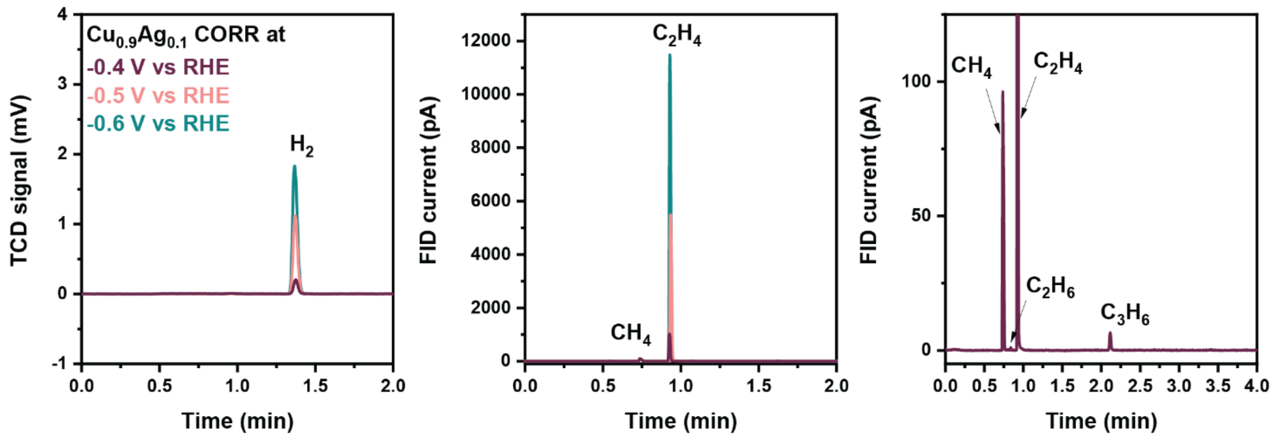

Figure S10. GC chromatograms (TCD and FID signals) measured during CORR catalyzed by $Cu_{0.9}Ag_{0.1}$ at -0.4 V, -0.5 V, and -0.6 V vs RHE – one measurement is shown for each potential.

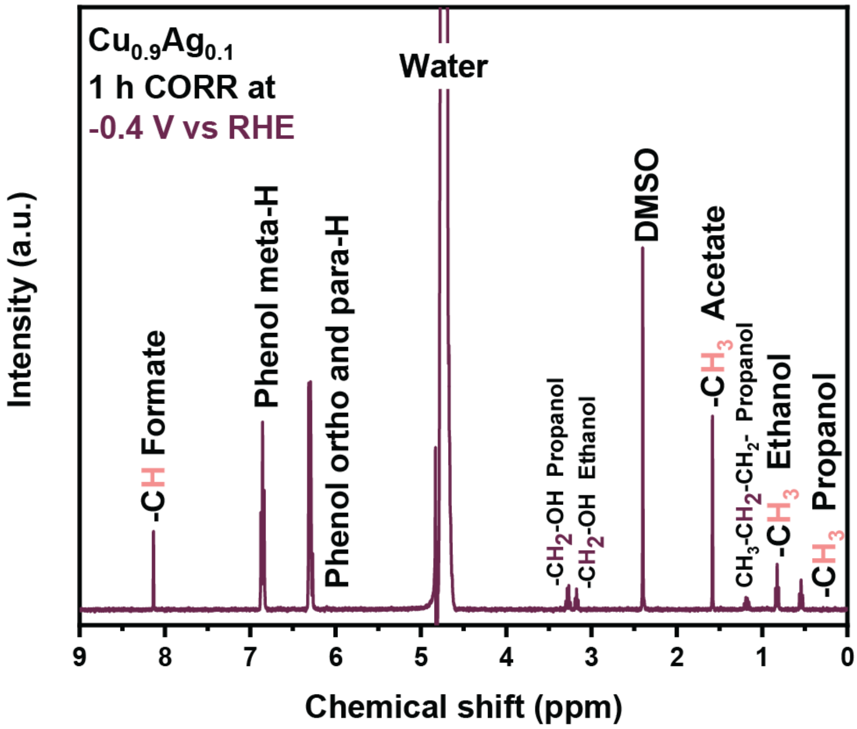

Figure S11. ^{1}H-NMR spectra of the catholyte measured after 1 h CORR at -0.4 V vs RHE using $Cu_{0.9}Ag_{0.1}$.

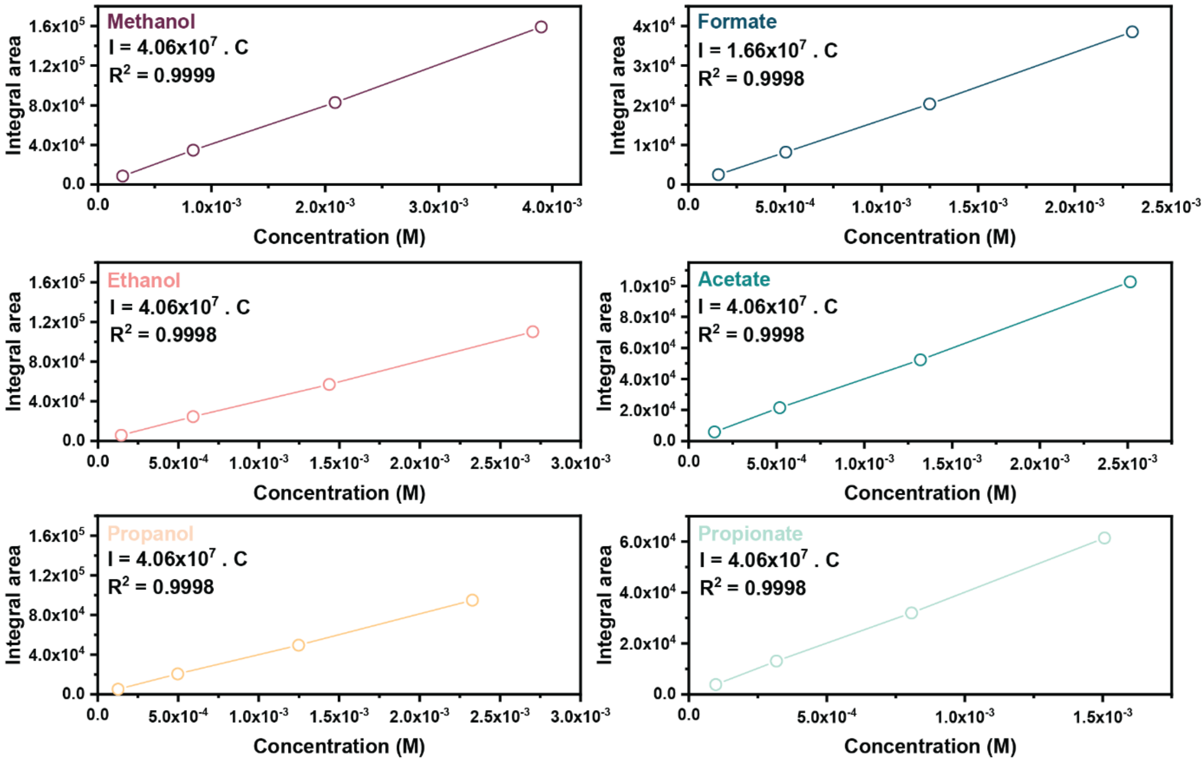

Figure S12. ^{1}H-NMR Calibration line of the different liquid products. It is important to note that while this external calibration demonstrates the linearity of the NMR response to a range of analyte concentration, we instead used internal calibration to calculate the concentration of products during our experiments. This is described in the experimental details at the beginning of this SI document.

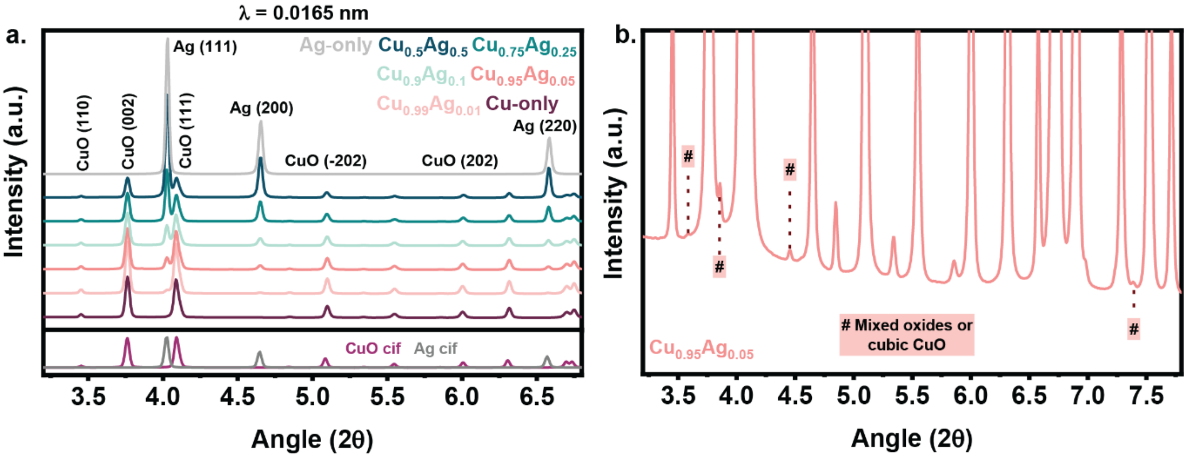

Figure S13. a) Diffractograms of the Cu-Ag bimetallic catalysts, CuO and Ag measured by WAXS and b) zoom in the diffractogram of $Cu_{0.95}Ag_{0.05}$.

Table S4. Positions of important reflections in the as-prepared samples.

Sample	Ag (200) (2θ)	Ag (220) (2θ)	CuO (110) (2θ)	CuO (002) (2θ)	CuO (202) (2θ)	CuO (-202) (2θ)
**Cu-only**	n.a.	n.a.	3.4526	3.7635	6.0033	5.0988
**$Cu_{0.99}Ag_{0.01}$**	4.6493	6.5786	3.4527	3.7635	6.0029	5.0989
**$Cu_{0.95}Ag_{0.05}$**	4.6499	6.5773	3.4526	3.7634	6.0039	5.0989
**$Cu_{0.9}Ag_{0.1}$**	4.6500	6.5773	3.4529	3.7629	6.0036	5.0984
**$Cu_{0.75}Ag_{0.25}$**	4.6511	6.5785	3.4531	3.7632	6.0072	5.0963
**$Cu_{0.5}Ag_{0.5}$**	4.6522	6.5800	3.4538	3.7636	6.0097	5.0955
**Ag-only**	4.6538	6.5824	n.a.	n.a.	n.a.	n.a.

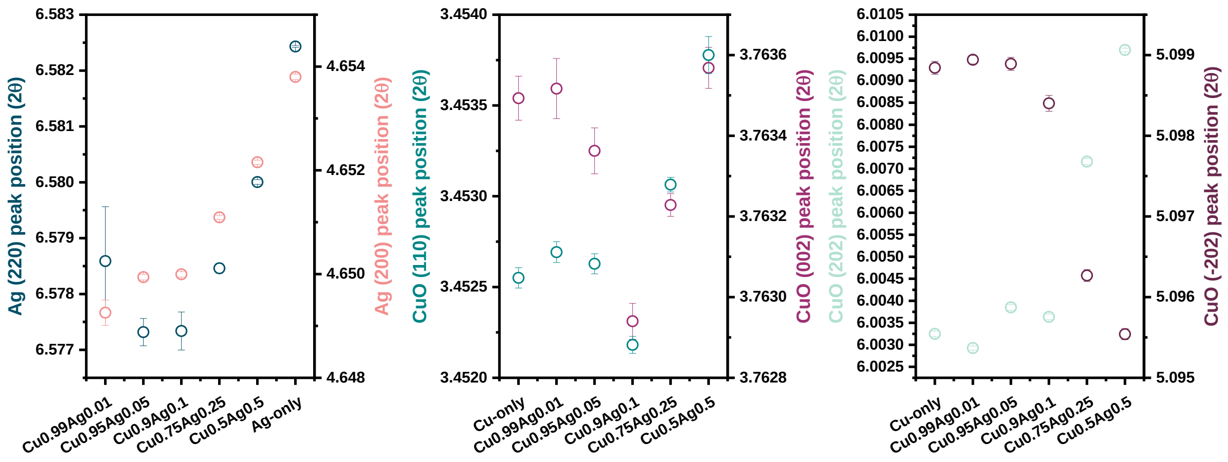

Figure S14. Influence of the sample composition on the position of important reflections.

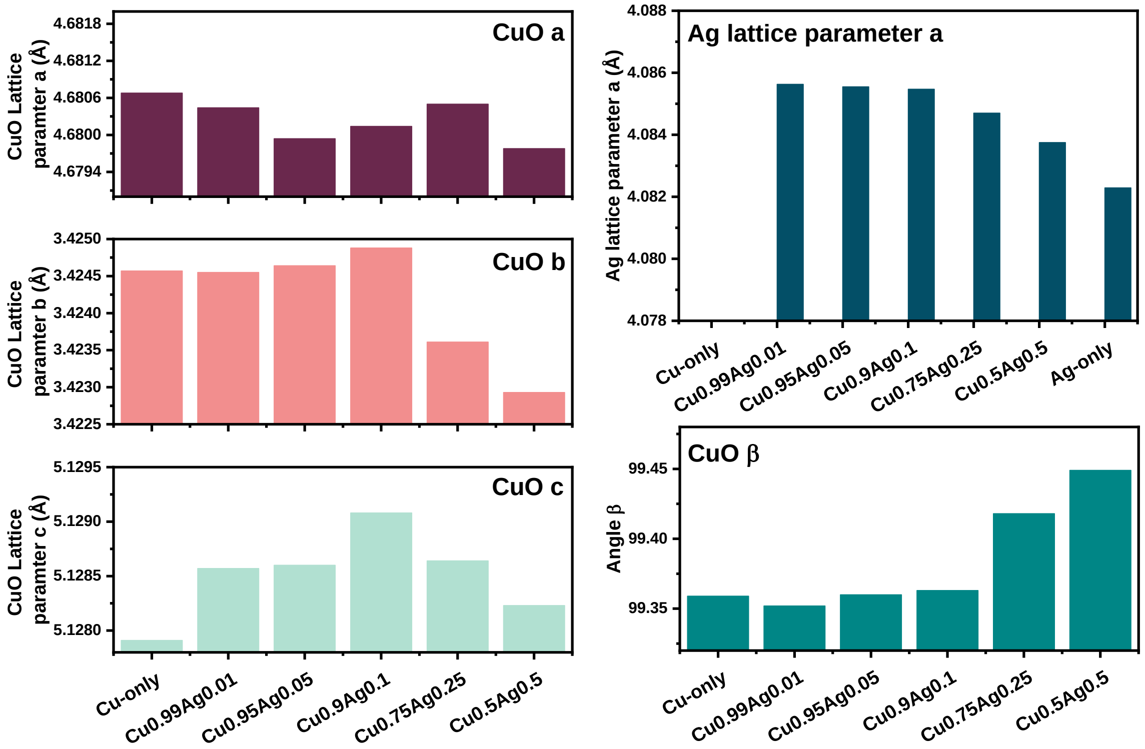

Figure S15. Rietveld refinement of monoclinic CuO and cubic Ag phases of the as-prepared samples measured by ex situ WAXS, refinement done using GSAS-II.

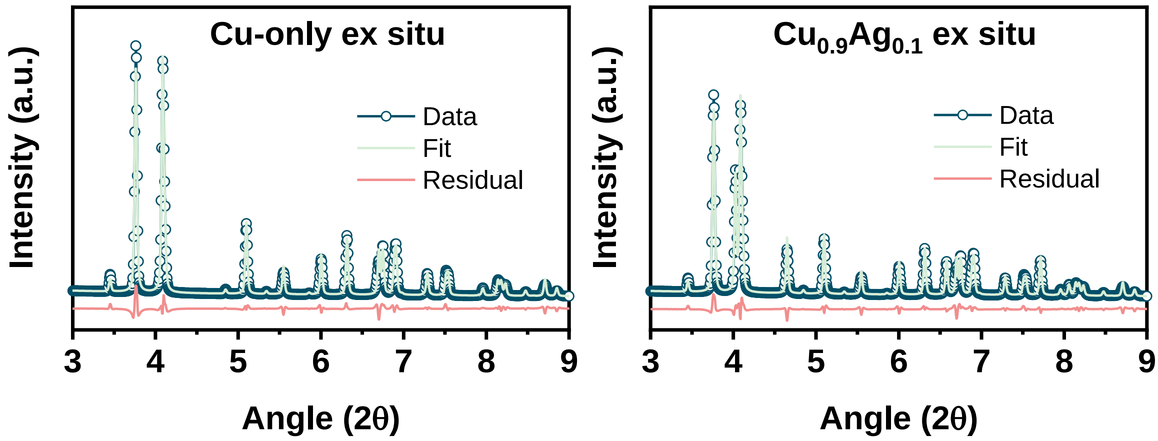

Figure S16. Rietveld refinement of as-prepared Cu and $Cu_{0.9}Ag_{0.1}$ measured by ex situ WAXS – Fit examples.

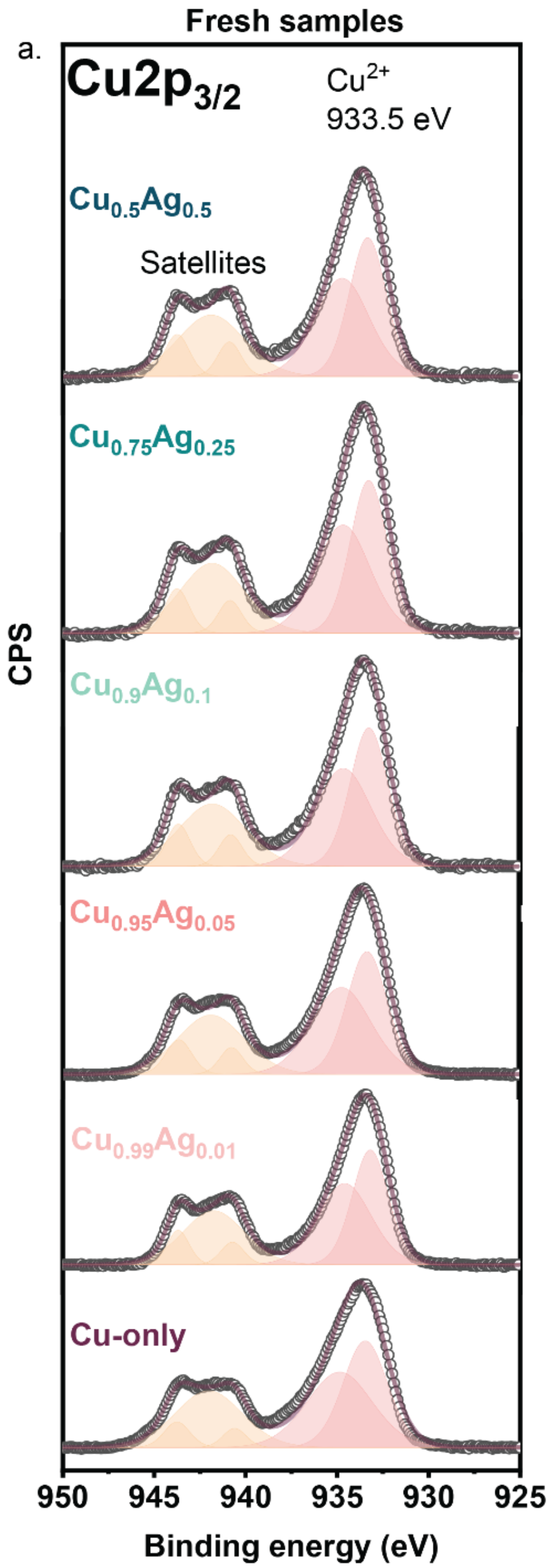

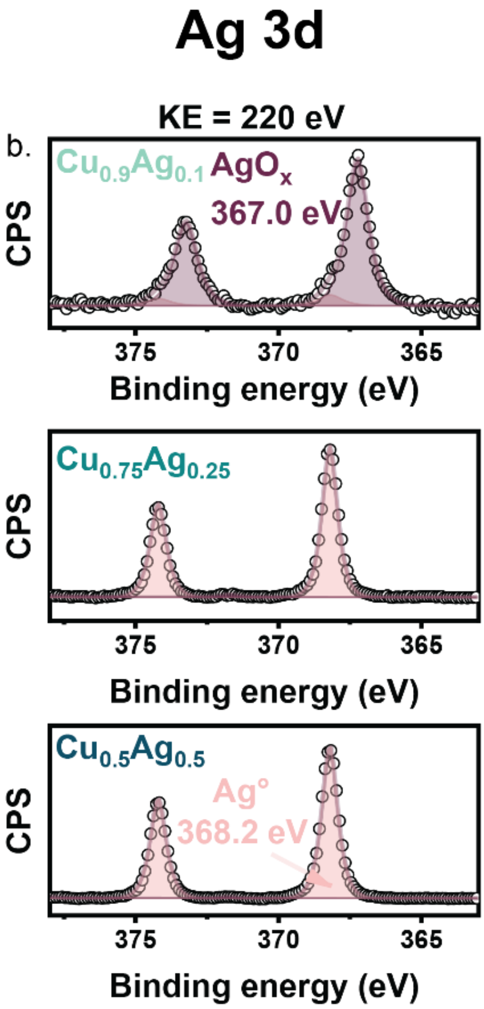

c.

	KE = 220 eV		KE = 570 eV		KE = 870 eV	
Atomic %	Ag	Cu	Ag	Cu	Ag	Cu
$Cu_{0.9}Ag_{0.1}$	6.2	93.8	3.5	96.5	5.1	94.9
$Cu_{0.75}Ag_{0.25}$	23.5	76.5	19.9	80.1	37.2	62.8
$Cu_{0.5}Ag_{0.5}$	43.0	57.0	60.1	39.9	54.8	45.2

Figure S17. a) Cu $2p_{3/2}$ XPS line of the as-prepared samples (Energy Al Kα), b) Ag 3d XPS line of $Cu_{0.9}Ag_{0.1}$, $Cu_{0.75}Ag_{0.25}$ and $Cu_{0.5}Ag_{0.5}$ measured at KE = 220 eV (HIPPIE, MAX IV, Lund, Sweden) and c) Ag and Cu atomic percentages of as-prepared $Cu_{0.9}Ag_{0.1}$, $Cu_{0.75}Ag_{0.25}$ and $Cu_{0.5}Ag_{0.5}$ measured at different KE (HIPPIE, MAX IV, Lund, Sweden).

Table S5. EXAFS fitting parameters of the as-prepared samples. The data was measured in transmission mode, except for $Cu_{0.99}Ag_{0.01}$ measured in fluorescence mode. An S_0^2 value of 0.88 was calculated from the fitting of Cu foil and was used for the fitting of k^3-weighted FT-EXAFS spectra of the samples containing Cu. An S_0^2 value of 0.79 was calculated from the fitting of Ag foil and was used for the fitting of k^3-weighted FT-EXAFS spectra of the samples containing Ag. The same ΔE_0 was used for all the shells. The calculations were carried out using the software Artemis.

Sample	Path	R (Å)	CN	σ^2 (Å^2)	ΔE_0 (eV)	R-factor (%)
**Cu-only**	Cu-O	1.957 ± 0.011	3.2 ± 0.4	0.004 ± 0.002	-1.2 ± 1.4	2.9
	Cu-Cu_1 (oxide)	2.906 ± 0.014	3.0 ± 0.9	0.006 ± 0.003		
	Cu-Cu_2 (oxide)	3.088 ± 0.014	2.7 ± 0.9	0.006 ± 0.003		
**$Cu_{0.99}Ag_{0.01}$ (Fluorescence data)**	Cu-O	1.955 ± 0.010	3.8 ± 0.5	0.004 ± 0.002	-1.4 ± 1.4	2.8
	Cu-Cu_1 (oxide)	2.903 ± 0.016	3.7 ± 1.2	0.007 ± 0.003		
	Cu-Cu_2 (oxide)	3.085 ± 0.016	3.2 ± 1.2	0.007 ± 0.003		
	Ag-Ag	2.868 ± 0.005	8.8 ± 0.6	0.009 ± 0.001	1.0 ± 0.5	0.8
**$Cu_{0.95}Ag_{0.05}$**	Cu-O	1.955 ± 0.011	3.1 ± 0.4	0.004 ± 0.002	-1.3 ± 1.4	3.1
	Cu-Cu_1 (oxide)	2.905 ± 0.015	3.1 ± 1.0	0.006 ± 0.003		
	Cu-Cu_2 (oxide)	3.087 ± 0.015	2.7 ± 0.9	0.006 ± 0.003		
	Ag-Ag	2.867 ± 0.003	11.0 ± 0.4	0.009 ± 0.001	0.0 ± 0.3	0.2
**$Cu_{0.9}Ag_{0.1}$**	Cu-O	1.957 ± 0.011	3.8 ± 0.5	0.005 ± 0.002	-1.4 ± 1.4	2.9
	Cu-Cu_1 (oxide)	2.905 ± 0.014	3.6 ± 1.1	0.006 ± 0.002		
	Cu-Cu_2 (oxide)	3.088 ± 0.014	3.4 ± 1.1	0.006 ± 0.002		
	Ag-Ag	2.868 ± 0.003	11.6 ± 0.4	0.009 ± 0.001	2.0 ± 0.3	0.2
**$Cu_{0.75}Ag_{0.25}$**	Cu-O	1.957 ± 0.011	3.9 ± 0.5	0.005 ± 0.002	-1.4 ± 1.3	2.7
	Cu-Cu_1 (oxide)	2.905 ± 0.014	3.8 ± 1.1	0.006 ± 0.002		
	Cu-Cu_2 (oxide)	3.088 ± 0.014	3.5 ± 1.1	0.006 ± 0.002		
	Ag-Ag	2.868 ± 0.002	11.6 ± 0.4	0.009 ± 0.001	0.7 ± 0.2	0.1
**$Cu_{0.5}Ag_{0.5}$**	Cu-O	1.958 ± 0.011	4.1 ± 0.5	0.005 ± 0.002	-1.1 ± 1.4	3.0
	Cu-Cu_1 (oxide)	2.906 ± 0.014	3.8 ± 1.2	0.006 ± 0.002		
	Cu-Cu_2 (oxide)	3.089 ± 0.014	3.6 ± 1.2	0.006 ± 0.002		
	Ag-Ag	2.867 ± 0.002	11.8 ± 0.4	0.010 ± 0.001	0.9 ± 0.3	0.1
**Ag-only**	Ag-Ag	2.868 ± 0.002	11.9 ± 0.4	0.010 ± 0.001	0.9 ± 0.2	0.1

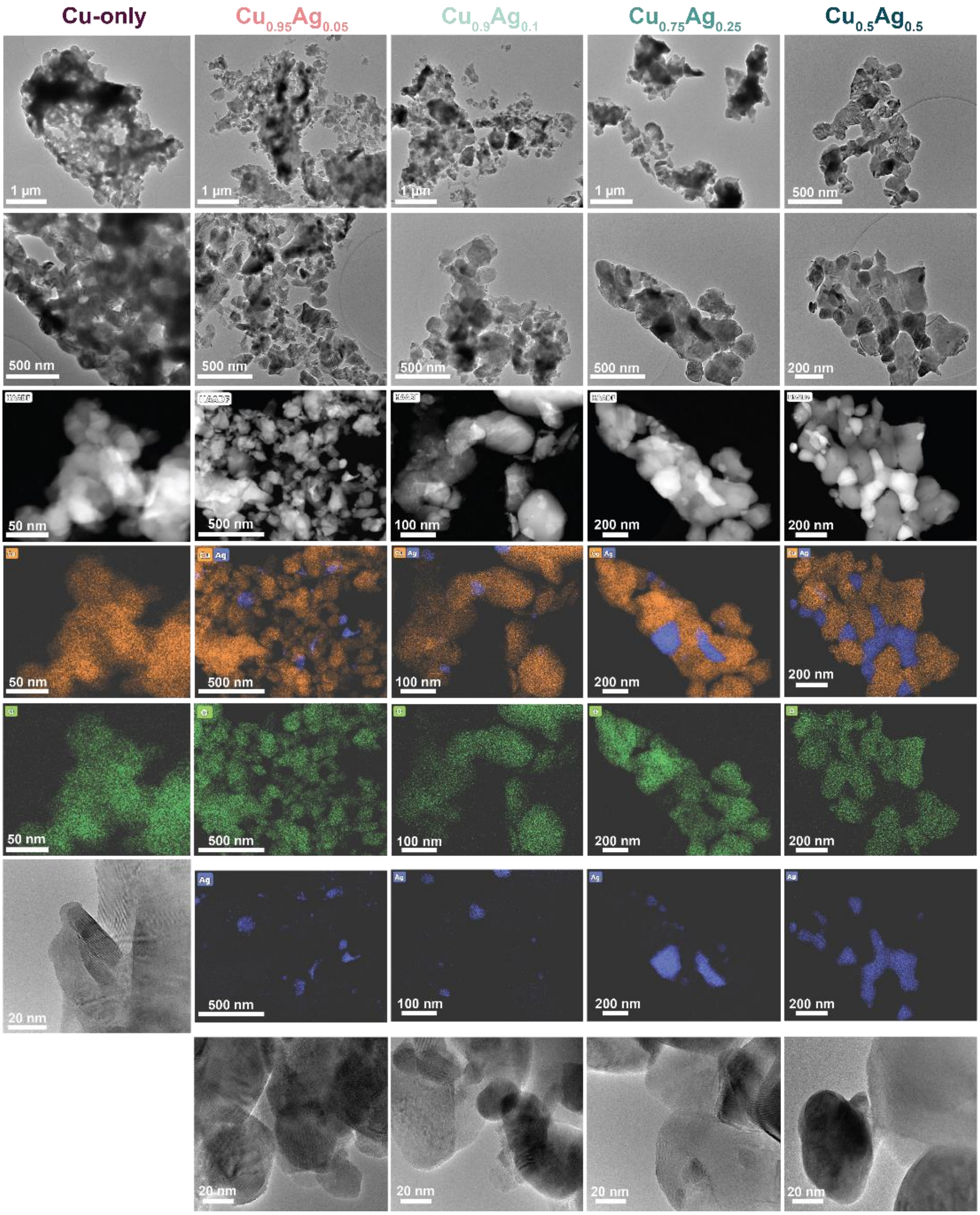

Figure S18. Bright-field TEM and HAADF-STEM images and EDX maps of the as-prepared catalysts.

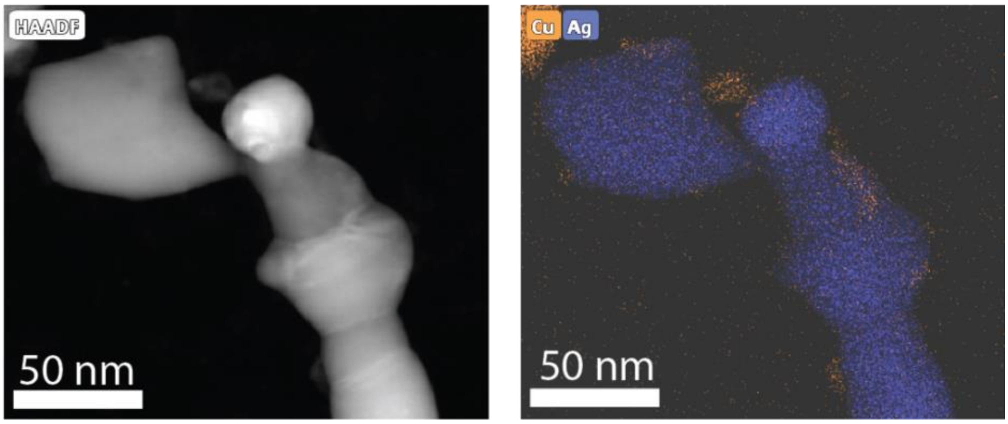

Figure S19. HAADF-STEM image and EDX map of $Cu_{0.95}Ag_{0.05}$ where CuO decorates Ag particles.

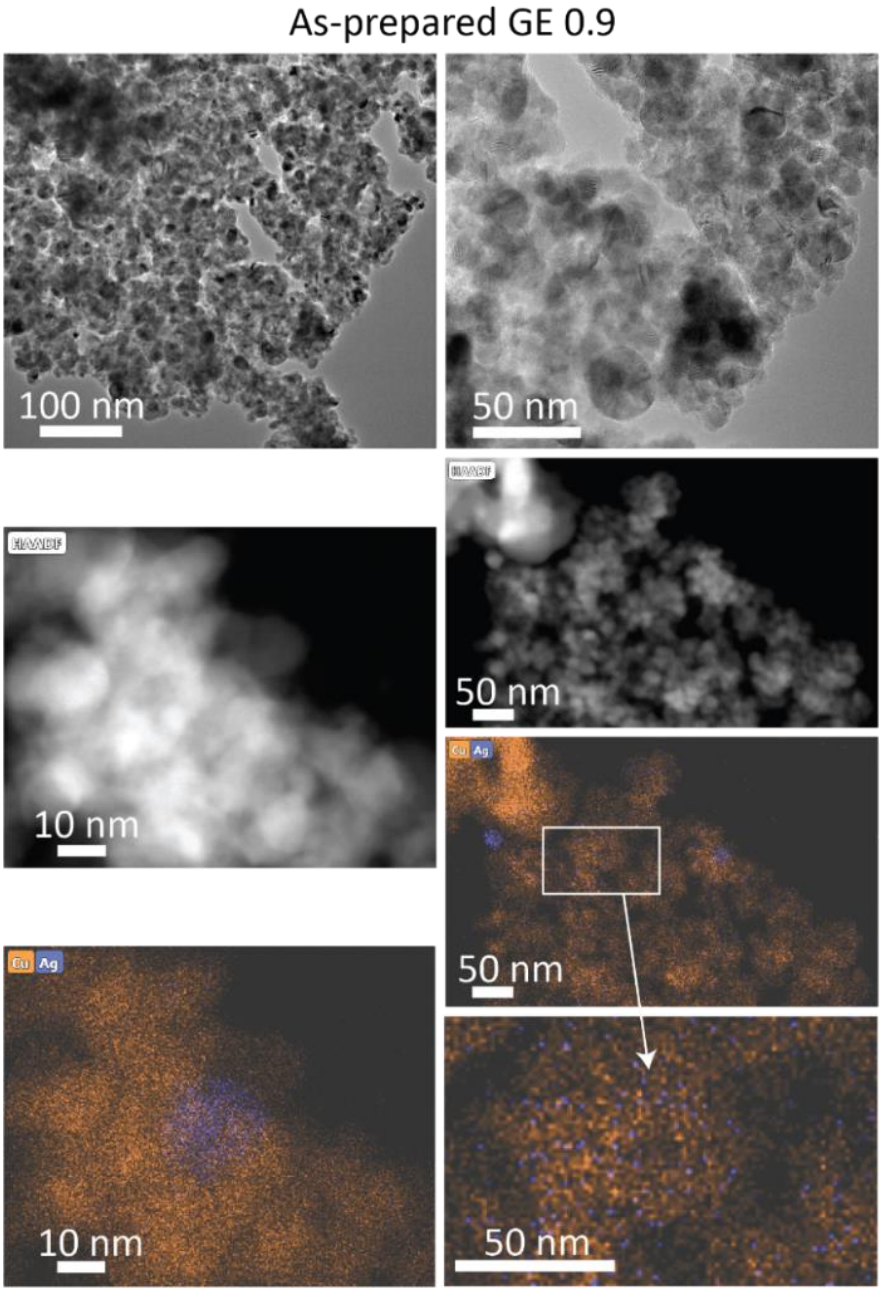

Figure S20. Bright-field TEM and HAADF-STEM images and EDX maps of the as-synthesized GE 0.9 sample prepared by galvanic exchange.

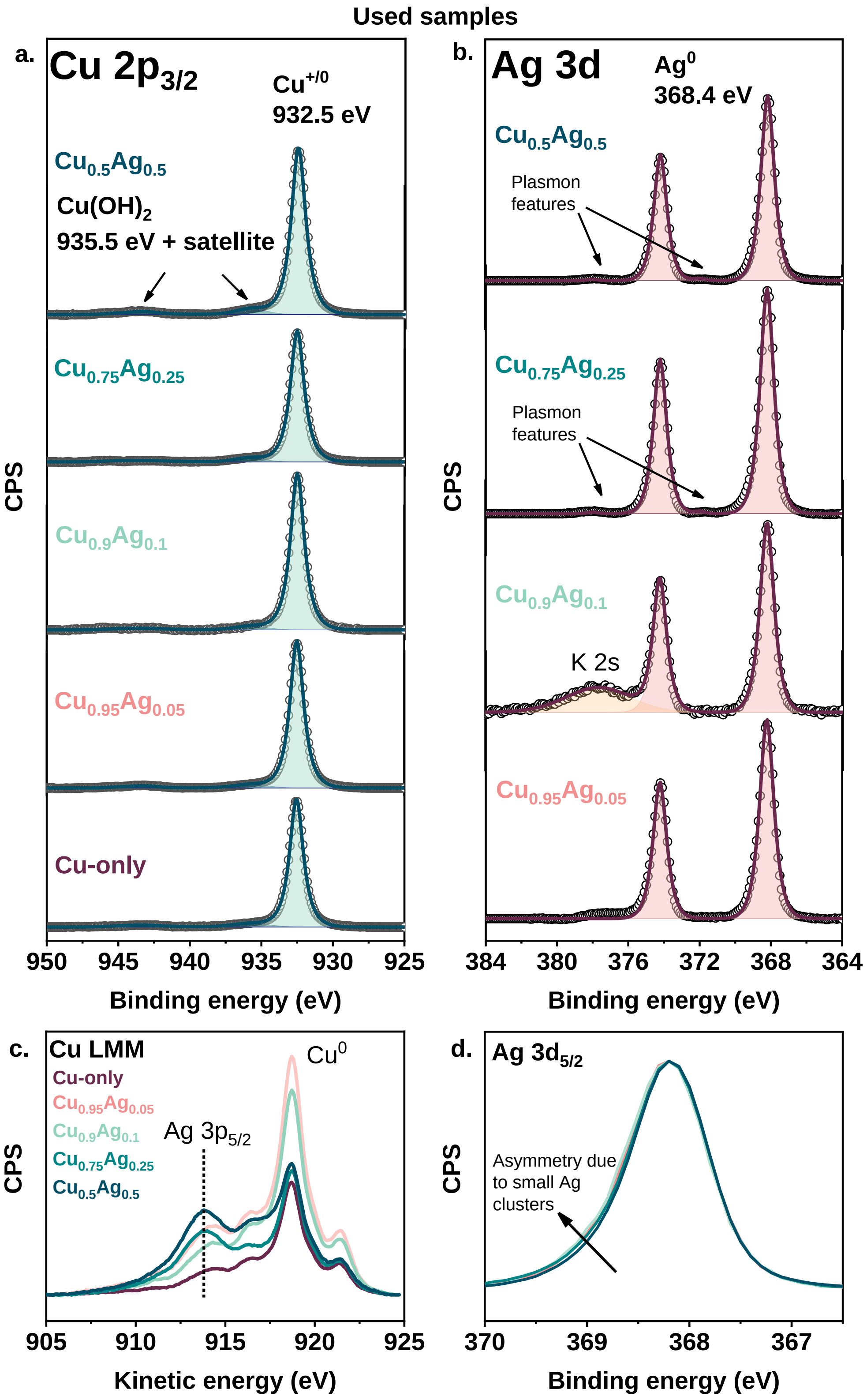

Figure S21. a) Cu $2p_{3/2}$, b) Ag 3d XPS lines, c) Cu LMM Auger lines, and d) Ag $3d_{5/2}$ XPS line asymmetry of the used Cu-Ag samples analyzed by quasi-in situ XPS (Al Kα source).

Table S6. Ag/Cu ratios measured by quasi in situ XPS of the as-prepared and used samples (reduction at -0.4 V vs RHE for 10 min) (energy Al Kα).

Ag/Cu ratios	As-prepared	After reduction at -0.4 V vs RHE
$Cu_{0.95}Ag_{0.05}$	0.04	0.076
$Cu_{0.9}Ag_{0.1}$	0.13	0.046
$Cu_{0.75}Ag_{0.25}$	0.44	0.47
$Cu_{0.5}Ag_{0.5}$	0.88	0.53

Table S7. Ag/Cu ratio of the fresh galvanically exchange sample (GE 0.9) compared to used $Cu_{0.9}Ag_{0.1}$ (after CA) measured by quasi-in situ XPS.

	Ag/Cu ratios
$Cu_{0.9}Ag_{0.1}$ after CA measured by quasi-in situ XPS (avg. of 2 meas. spots)	0.046
GE 0.9 (avg. of 2 meas. spots)	0.048

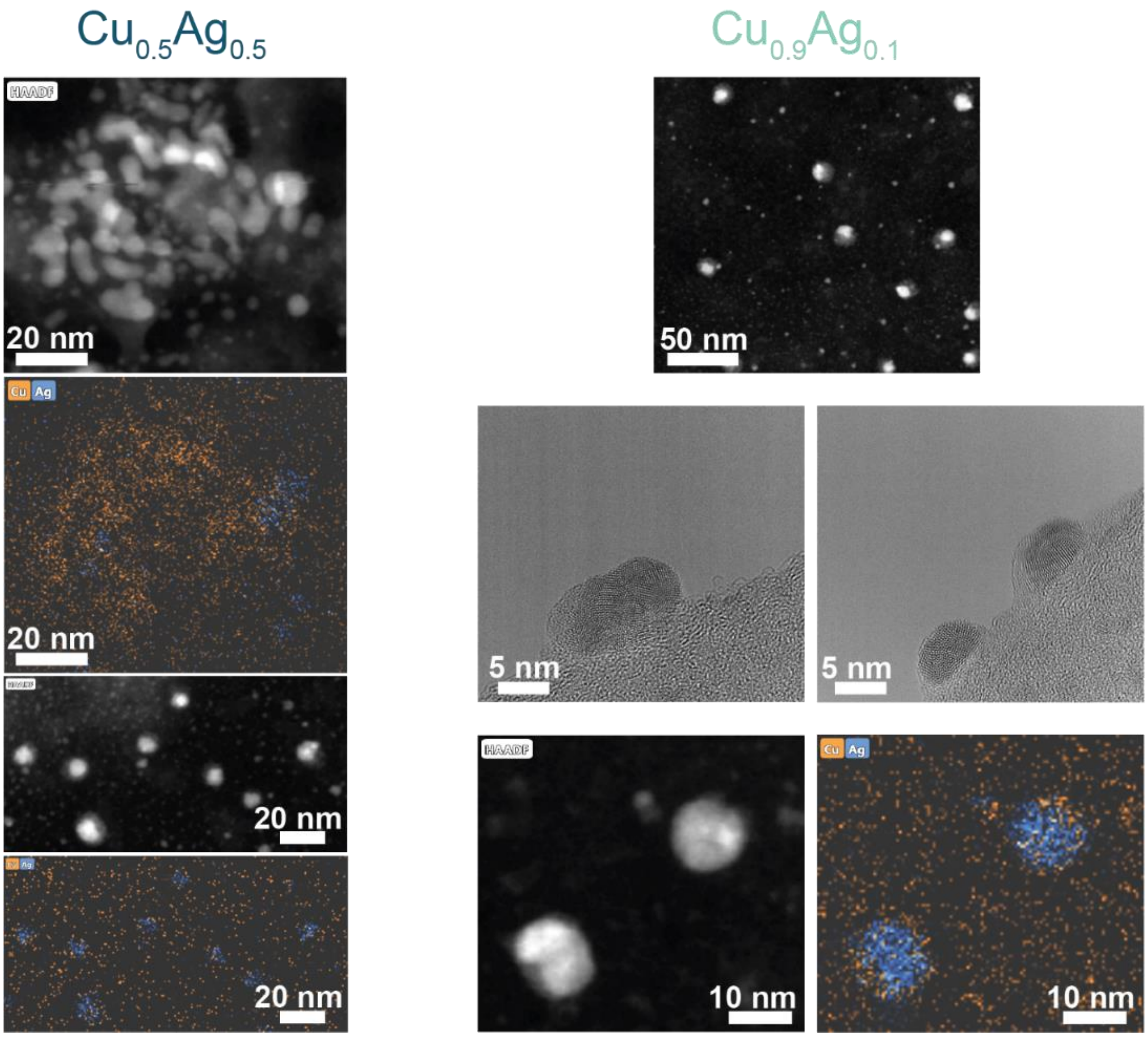

Figure S22. HAADF-STEM images and EDX maps of $Cu_{0.5}Ag_{0.5}$ and $Cu_{0.9}Ag_{0.1}$ after CORR at -0.4 V vs RHE, 1 h, 3 M KOH.

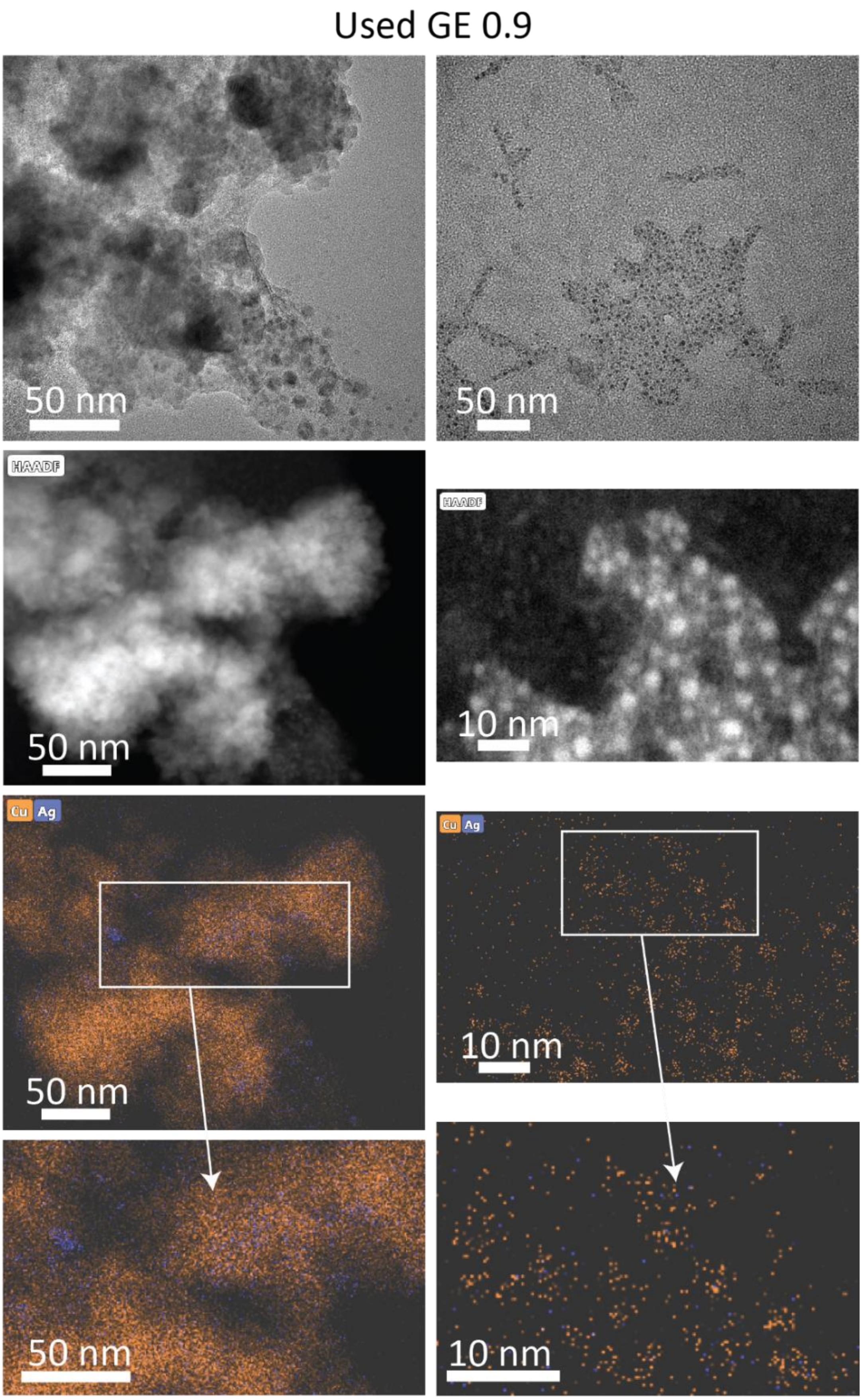

Figure S23. Bright field TEM, HAADF-STEM and EDX mapping images of GE 0.9 after CORR at -0.4 V vs RHE, 1 h, 3 M KOH.

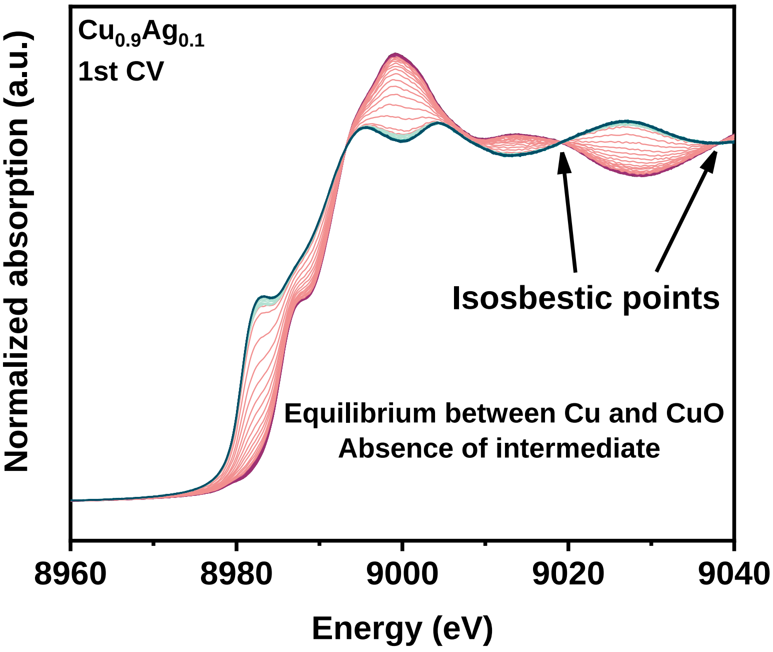

Figure S24. Cu K-edge of $Cu_{0.9}Ag_{0.1}$ during first CV from +0.5 V to -0.6 V vs RHE, 3 M KOH - The presence of isosbestic points demonstrates the direct reduction of CuO to Cu without intermediate.

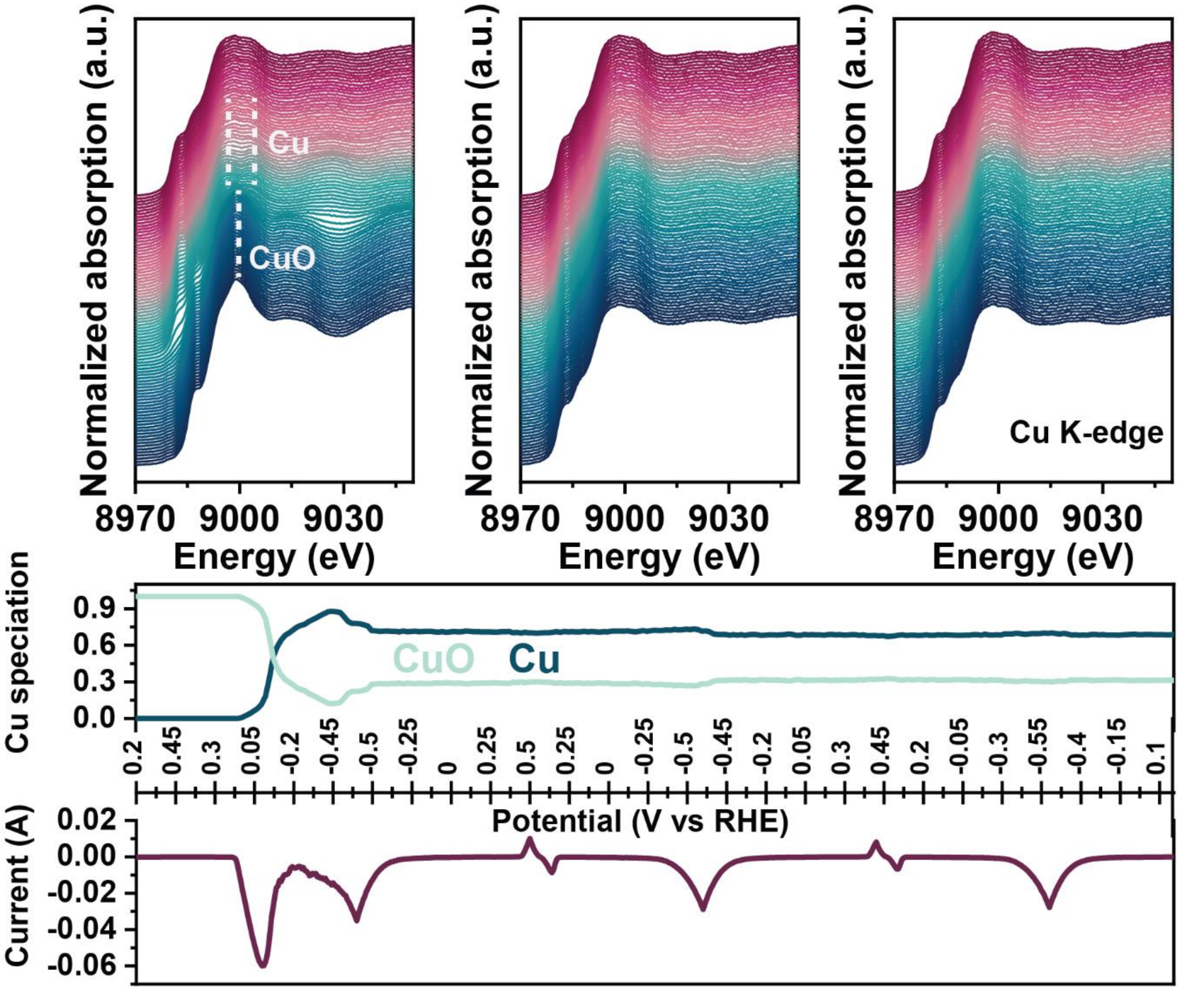

Figure S25. Cu K-edge XANES and k^3-weighted FT-EXAFS spectra of Cu-only during the three CV cycles in nitrogen, 3 M KOH, start +0.2 V vs RHE, from +0.5 V to -0.6 V vs RHE, 5 mV.s^{-1} – Linear combination fitting and CV signal are shown.

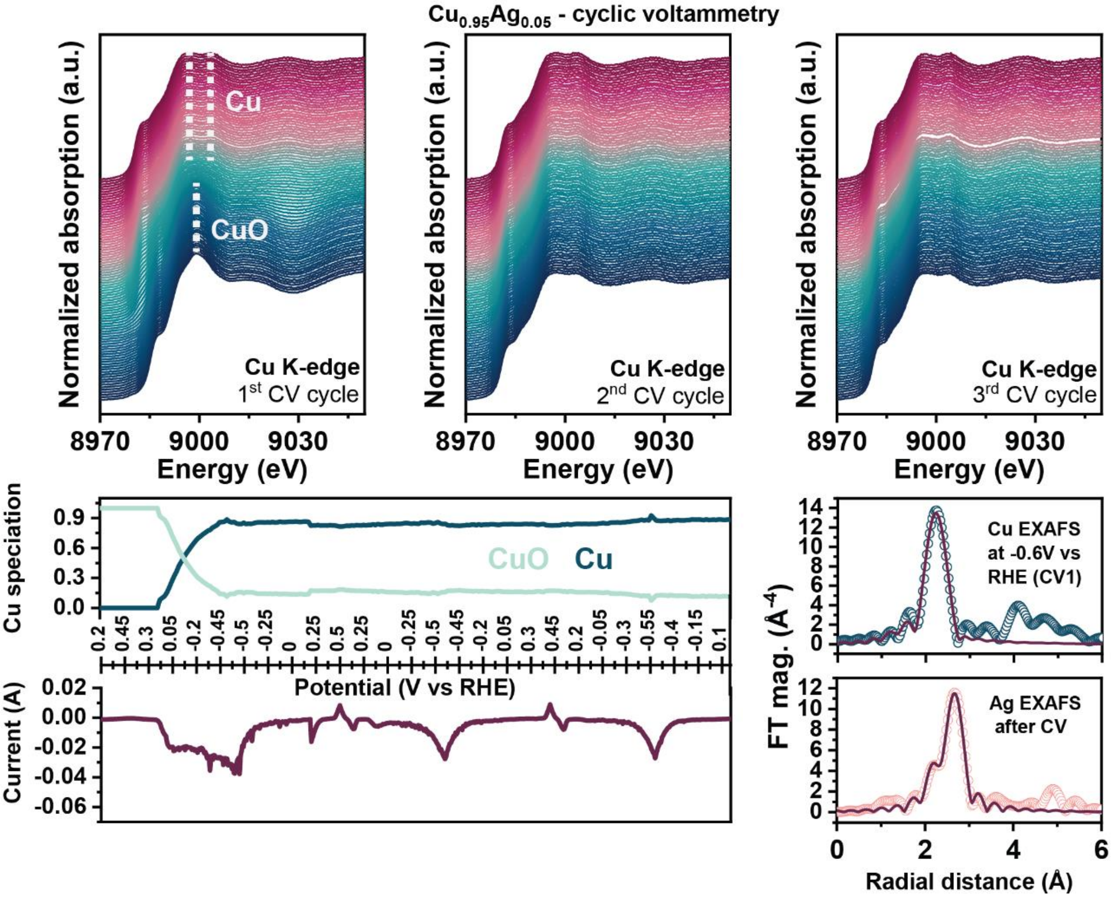

Figure S26. Cu K-edge XANES and k^3-weighted FT-EXAFS spectra of $Cu_{0.95}Ag_{0.05}$ during the three CV cycles in nitrogen, 3 M KOH, start +0.2 V vs RHE, from +0.5 V to -0.6 V vs RHE, 5 mV.s^{-1} – Linear combination fitting and CV signal are shown.

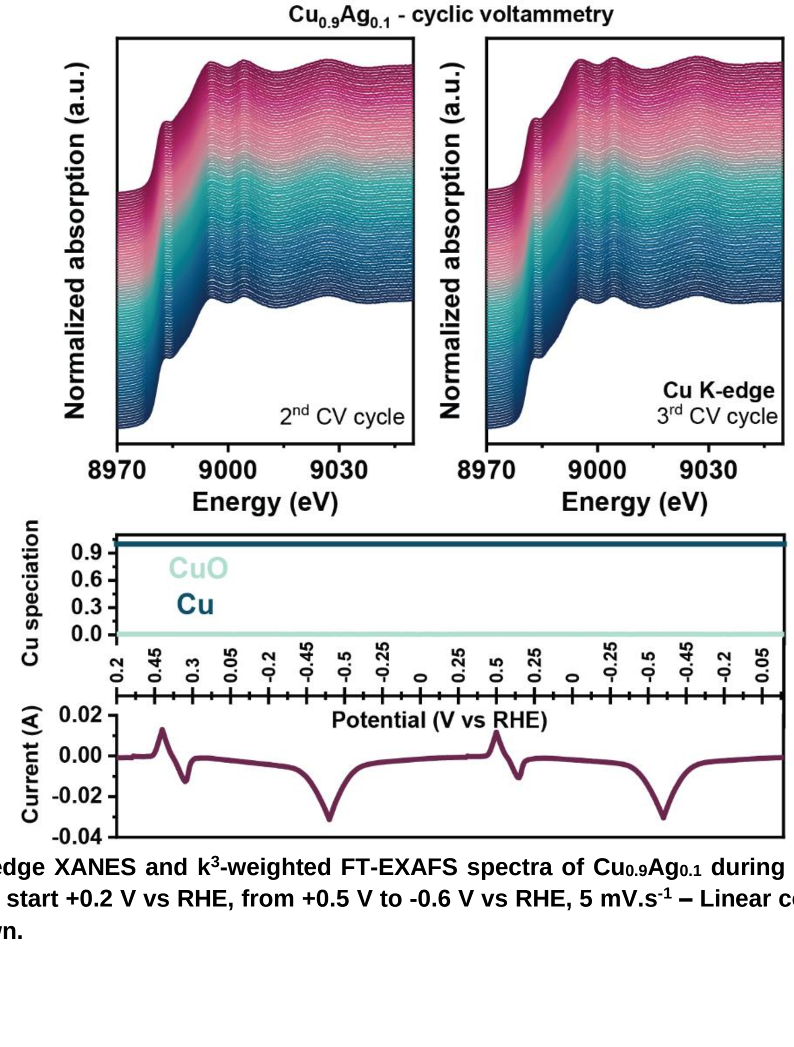

Figure S27. Cu K-edge XANES and k^3-weighted FT-EXAFS spectra of $Cu_{0.9}Ag_{0.1}$ during the two CV cycles in nitrogen, 3 M KOH, start +0.2 V vs RHE, from +0.5 V to -0.6 V vs RHE, 5 mV.s^{-1} – Linear combination fitting and CV signal are shown.

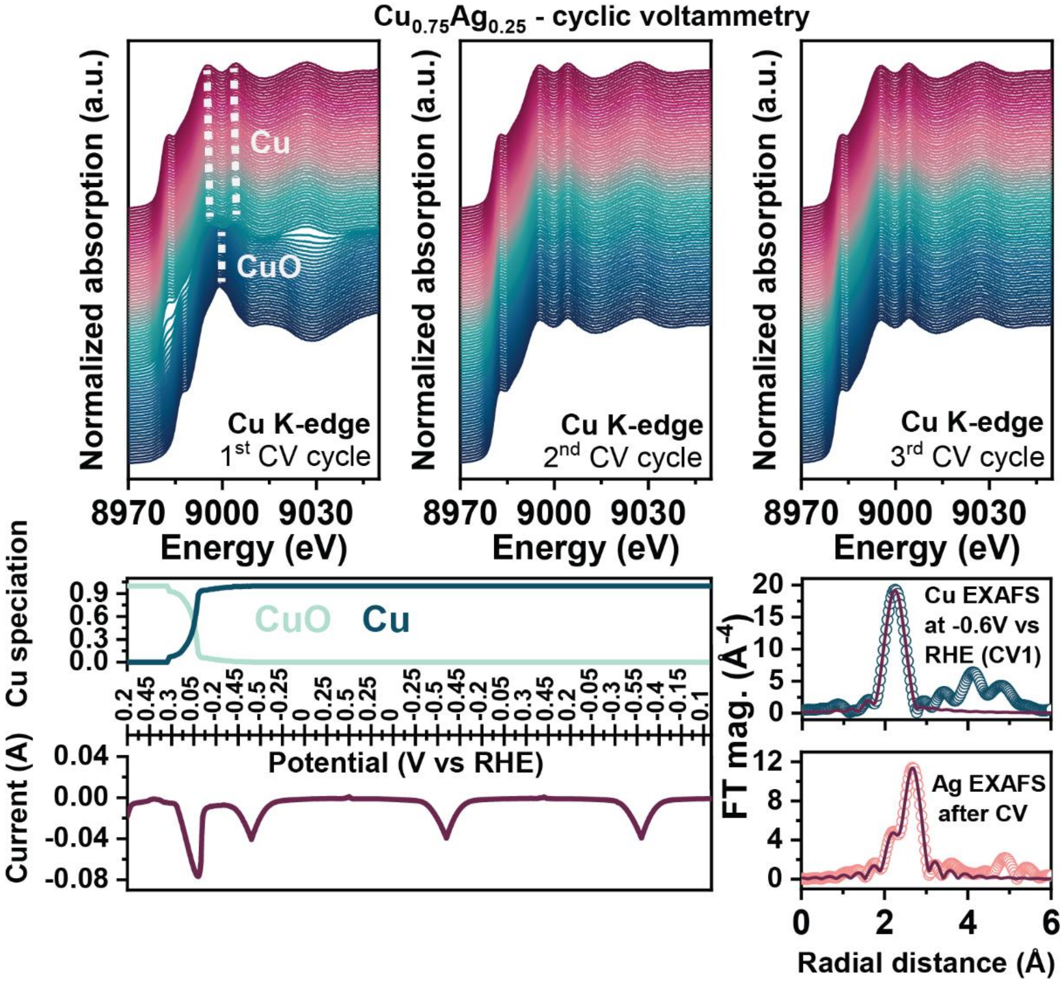

Figure S28. Cu K-edge XANES and k^3-weighted FT-EXAFS spectra of $Cu_{0.75}Ag_{0.25}$ during the three CV cycles in nitrogen, 3 M KOH, start +0.2 V vs RHE, from +0.5 V to -0.6 V vs RHE, 5 $mV.s^{-1}$ – Linear combination fitting and CV signal are shown.

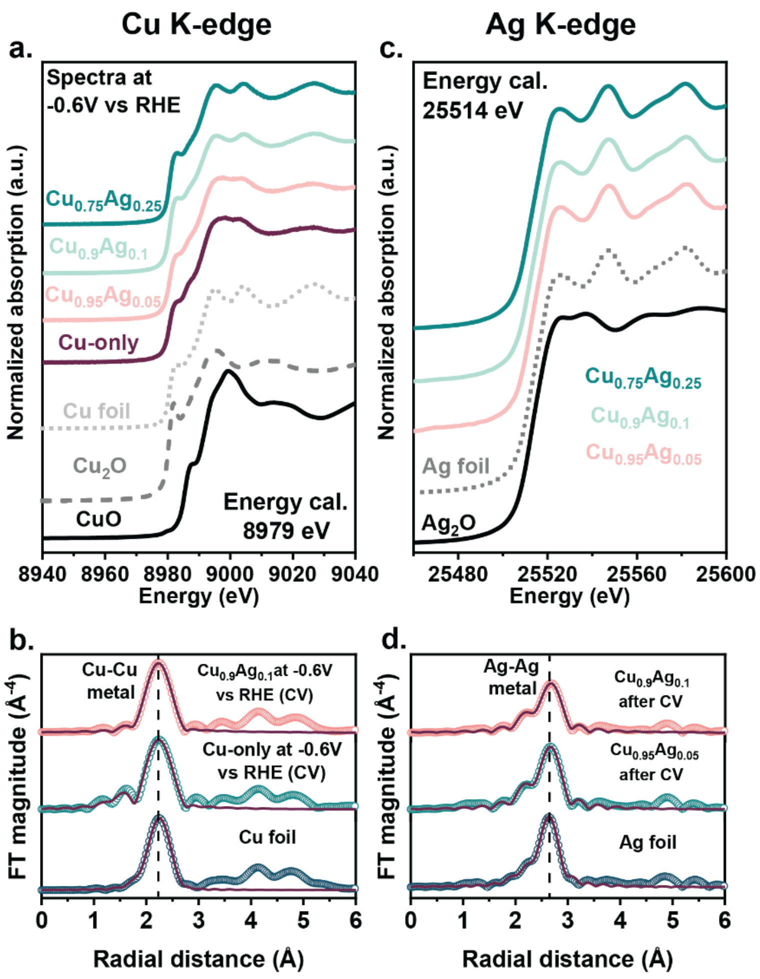

Figure S29. a) Cu K-edge XANES spectra recorded at the most negative potential of the first CV, -0.6 V vs RHE, b) Cu k^3-weighted FT-EXAFS recorded at the most negative potential of the first CV, -0.6 V vs RHE (10 spectra averaged), c) Ag K-edge XANES spectra recorded at the end of the first CV, and d) Ag k^3-weighted FT-EXAFS of the samples recorded at the end of the first CV (average of data measured over 15 min).

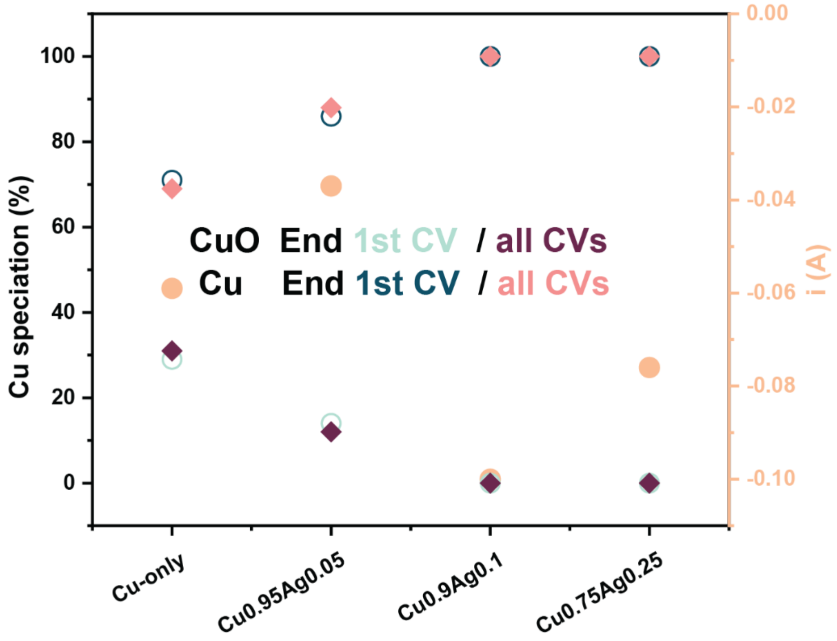

Figure S30. Phase composition of the samples after the first CV and after all CVs, measured by XAS (ROCK, SOLEIL synchrotron), quantified by linear combination fitting using Larch software – Maximum current reached during the reduction of CuO to Cu during the first CV cycle.

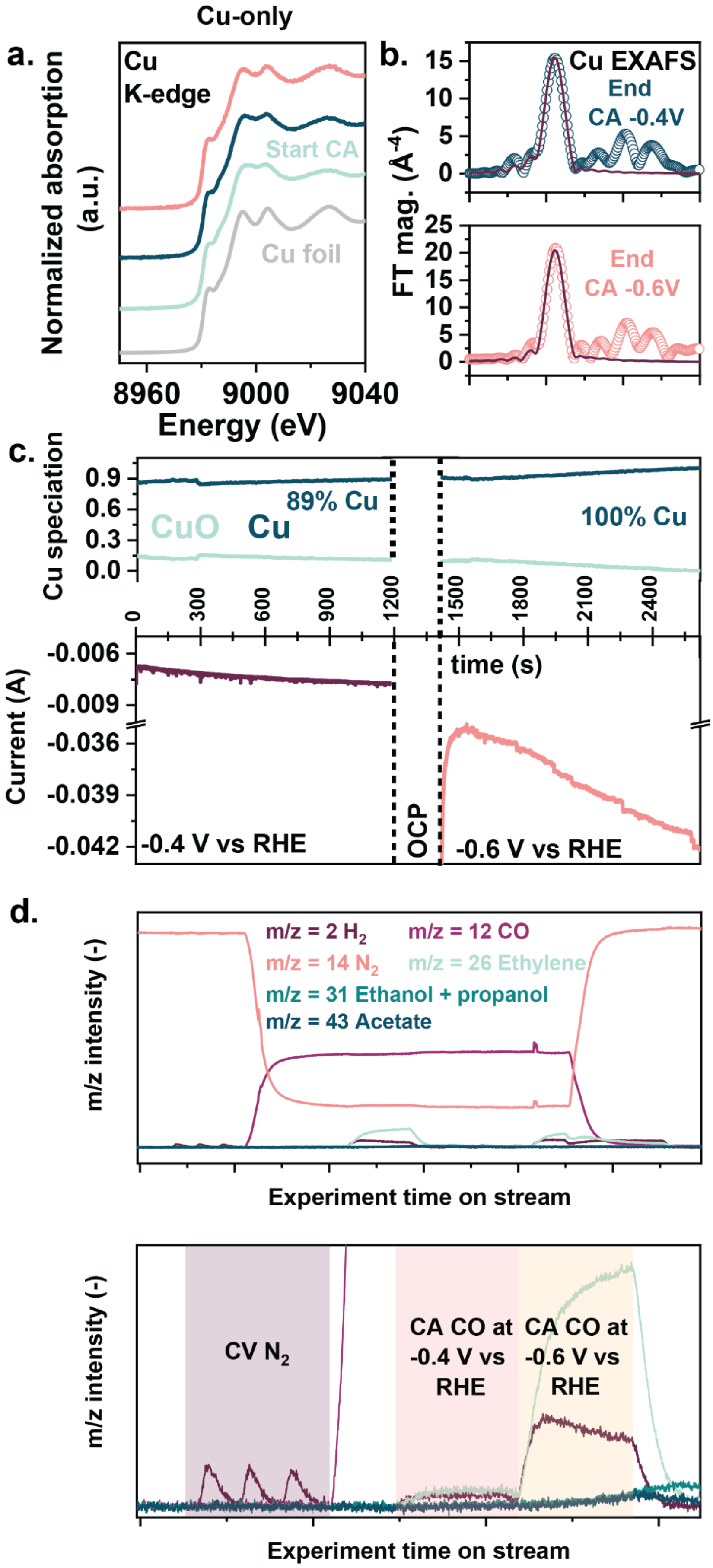

Figure S31. a) Cu K-edge XANES and b) k^3-weighted FT-EXAFS of Cu-only at the end of the chronoamperometry at -0.4 V vs RHE and -0.6 V vs RHE, CO, 3 M KOH, c) Linear combination fitting and CA measurements at -0.4 V and -0.6 V vs RHE and d) m/z intensities of the measured products during the CV in nitrogen (presented earlier) and the CA in CO at -0.4 V and -0.6 V vs RHE.

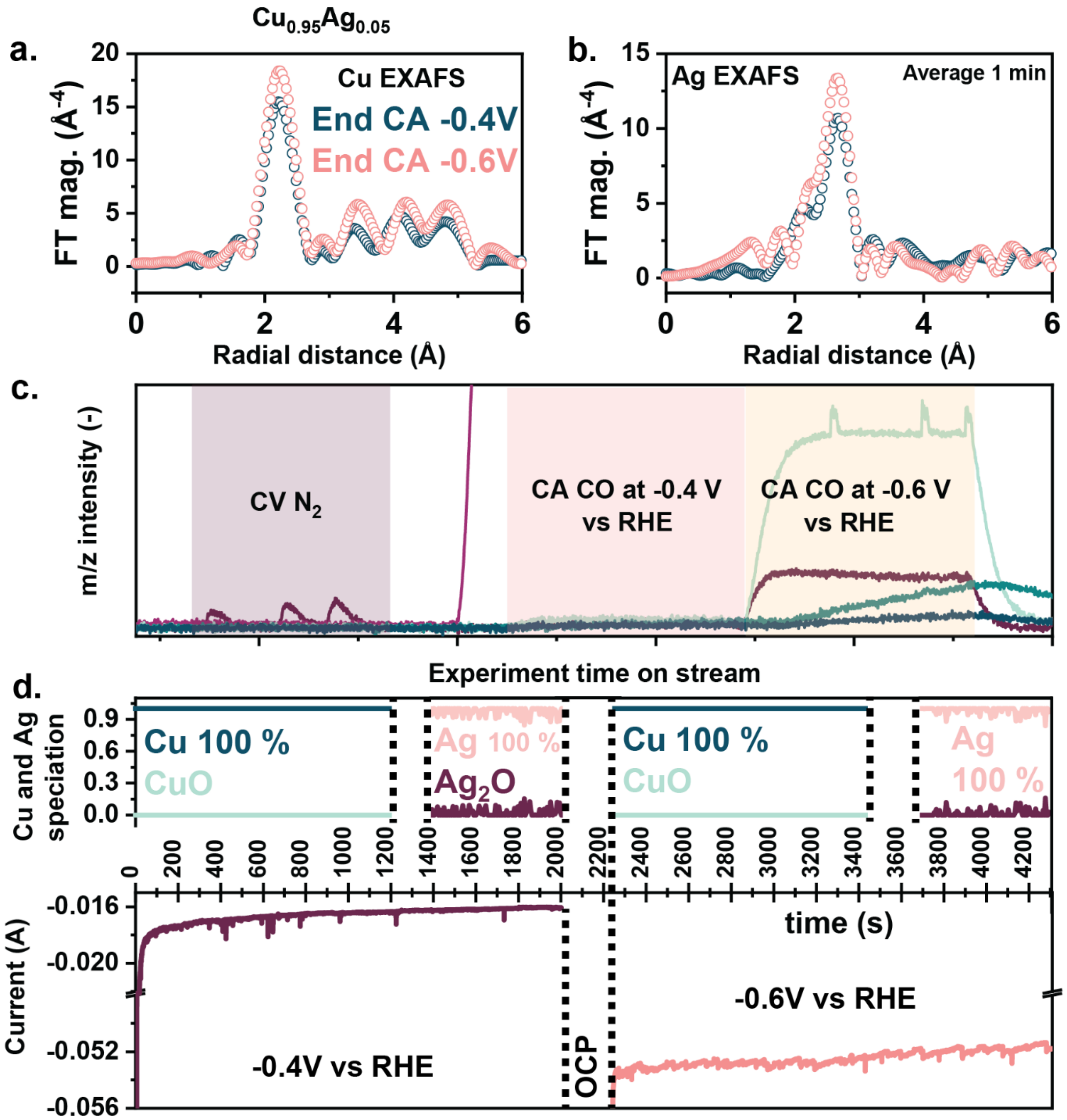

Figure S32. a) Cu k^3-weighted FT-EXAFS and b) Ag k^3-weighted FT-EXAFS of $Cu_{0.95}Ag_{0.05}$ at the end of the chronoamperometry at -0.4 V vs RHE and -0.6 V vs RHE, CO, 3 M KOH, c) m/z intensities of the measured products during the CV in nitrogen (presented earlier) and the CA in CO at -0.4 V and -0.6 V vs RHE and d) Linear combination fitting and CA measurements at -0.4 V and -0.6 V vs RHE.

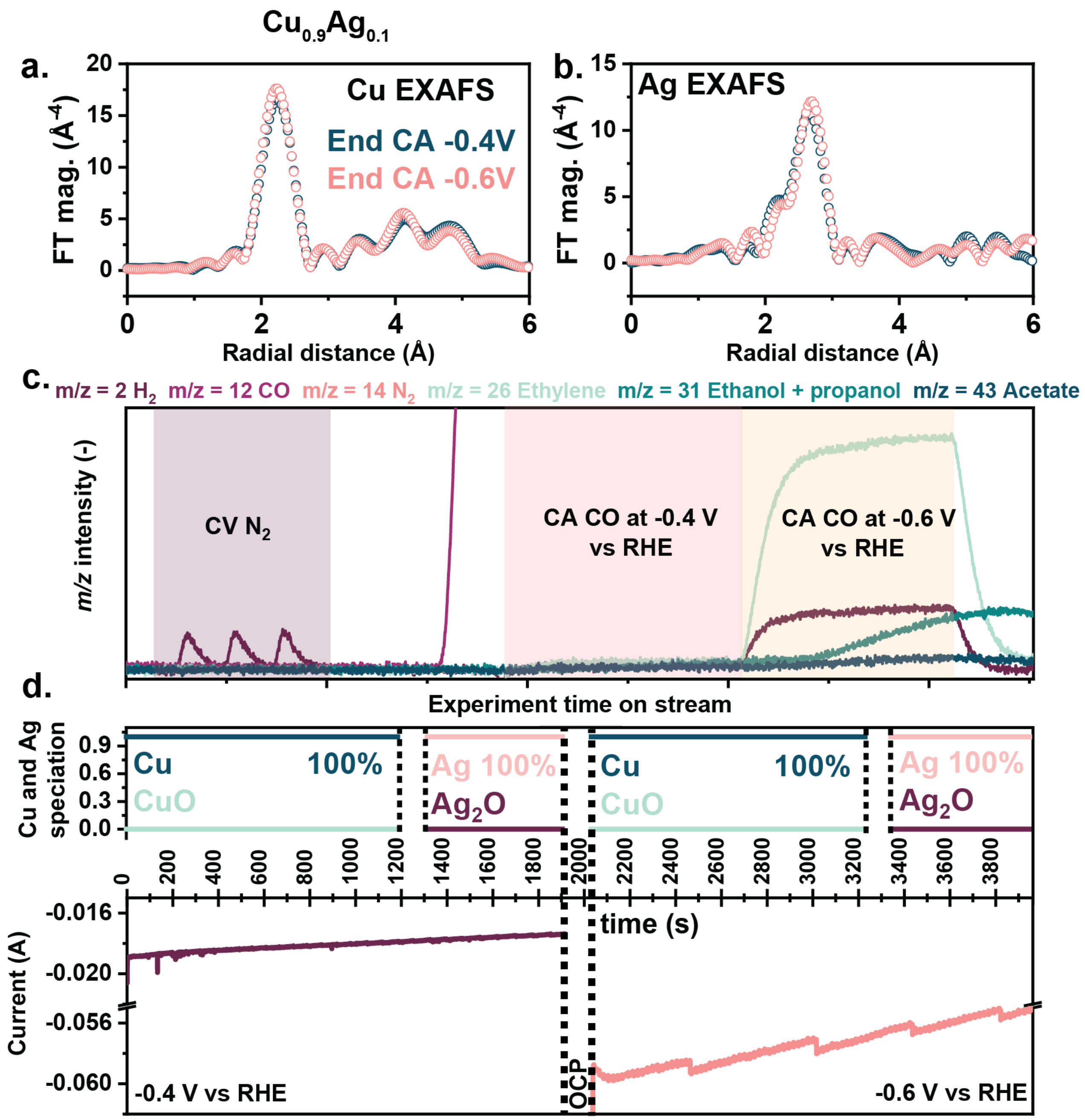

Figure S33. a) Cu k³-weighted FT-EXAFS and b) Ag k³-weighted FT-EXAFS of $Cu_{0.9}Ag_{0.1}$ at the end of the chronoamperometry at -0.4 V vs RHE and -0.6 V vs RHE, CO, 3 M KOH, c) m/z intensities of the measured products during the CV in nitrogen (presented earlier) and the CA in CO at -0.4 V and -0.6 V vs RHE and d) Linear combination fitting and CA measurements at -0.4 V and -0.6 V vs RHE.

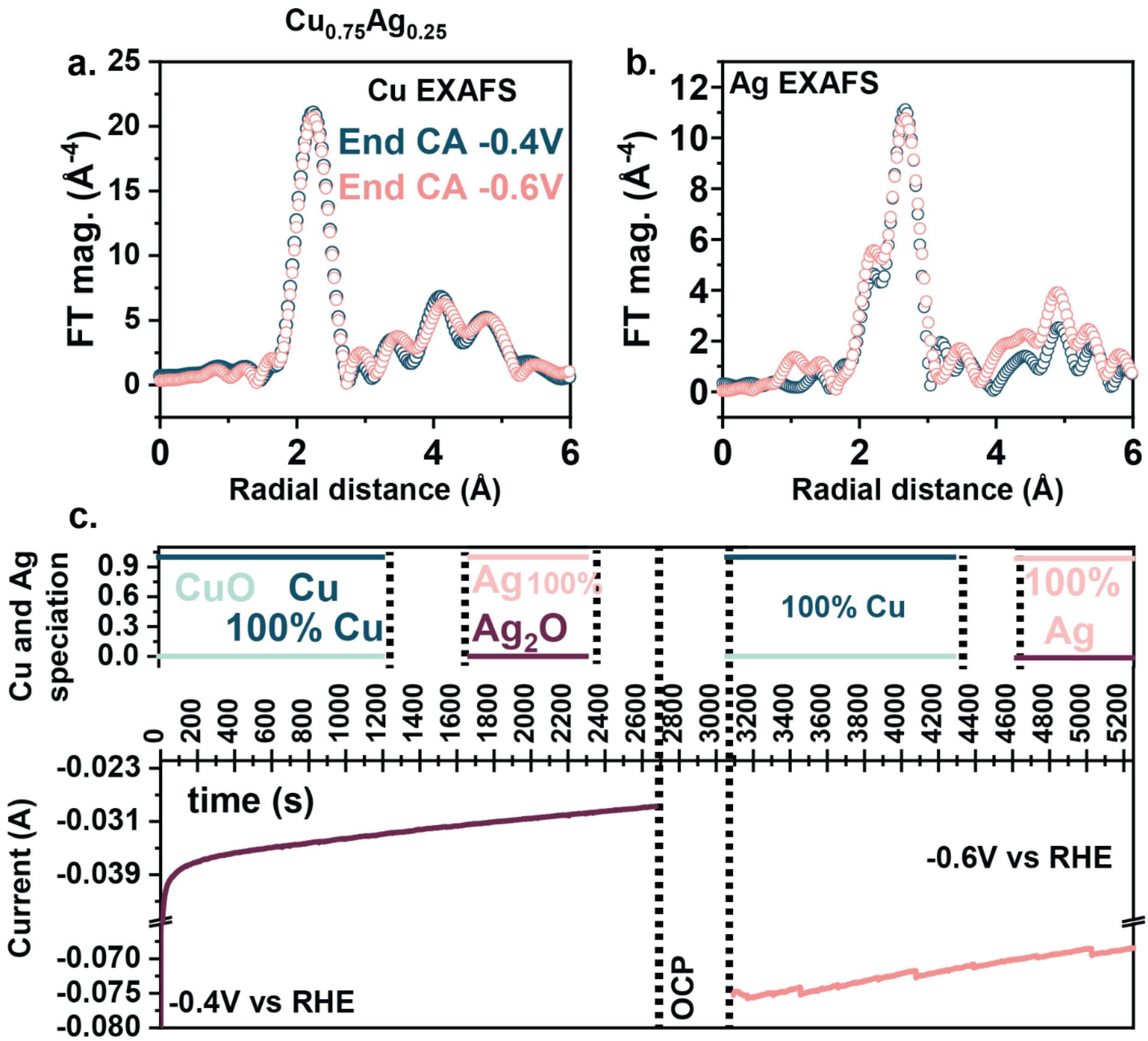

Figure S34. a) Cu k^3-weighted FT-EXAFS and b) Ag k^3-weighted FT-EXAFS of $Cu_{0.75}Ag_{0.25}$ at the end of the chronoamperometry at -0.4 V vs RHE and -0.6 V vs RHE, CO, 3 M KOH, and c) Linear combination fitting and CA measurements at -0.4 V and -0.6 V vs RHE.

Table S8. EXAFS fitting parameters of the in situ measured samples (data measured in fluorescence mode). An S_0^2 value of 0.88 was calculated from the fitting of Cu foil and was used for the fitting of k^3-weighted FT-EXAFS spectra of the samples containing Cu. An S_0^2 value of 0.79 was calculated from the fitting of Ag foil and was used for the fitting of k^3-weighted FT-EXAFS spectra of the samples containing Ag. The ΔE_0 was kept the same for all the shells of each element).

In situ sample	Measurement	Path	R (Å)	CN	σ^2 (Å^2)	ΔE_0 (eV)	R-factor (%)
Cu-only	CV at -0.6V vs RHE	Cu-O (oxide)	1.896 ± 0.033	1.0 ± 0.6	0.006 ± 0.009	4.0 ± 2.2	2.2
		Cu-Cu (metal)	2.541 ± 0.014	4.9 ± 1.0	0.007 ± 0.001		
	CA at -0.4V vs RHE (last spectrum)	Cu-O (oxide)	1.894 ± 0.070	0.9 ± 1.6	0.02 ± 0.04	2.5 ± 1.5	1.1
		Cu-Cu (metal)	2.535 ± 0.010	7.3 ± 1.1	0.009 ± 0.001		
	CA at -0.6V vs RHE (last spectrum)	Cu-Cu (metal)	2.538 ± 0.008	8.0 ± 1.0	0.007 ± 0.001	2.6 ± 1.3	1.2
$Cu_{0.95}Ag_{0.05}$	CV at -0.6V vs RHE	Cu-O (oxide)	1.886 ± 0.042	0.9 ± 0.8	0.01 ± 0.02	3.3 ± 1.8	2.2
		Cu-Cu (metal)	2.537 ± 0.011	5.3 ± 0.9	0.007 ± 0.001		
		Ag-Ag	2.865 ± 0.003	12.1 ± 0.6	0.0100 ± 0.0004	2.0 ± 0.3	0.3
	CA at -0.4V vs RHE (last spectrum)	Cu-Cu (metal)	2.534 ± 0.008	6.1 ± 0.8	0.007 ± 0.001	2.0 ± 1.3	1.1
		Ag-Ag	2.857 ± 0.006	12.0 ± 1.0	0.0100 ± 0.0008	0.6 ± 0.6	1.2
	CA at -0.6V vs RHE (last spectrum)	Cu-Cu (metal)	2.537 ± 0.008	7.8 ± 0.9	0.008 ± 0.001	2.9 ± 1.2	1.1
		Ag-Ag	2.864 ± 0.006	11.0 ± 1.0	0.0080 ± 0.0007	2.2 ± 0.6	1.0
$Cu_{0.9}Ag_{0.1}$	CV at -0.6V vs RHE	Cu-Cu (metal)	2.539 ± 0.005	6.7 ± 0.5	0.008 ± 0.001	3.2 ± 0.8	0.7
		Ag-Ag	2.868 ± 0.003	11.6 ± 0.5	0.009 ± 0.001	2.3 ± 0.3	0.2
	CA at -0.4V vs RHE (last spectrum)	Cu-Cu (metal)	2.542 ± 0.006	7.0 ± 0.6	0.008 ± 0.001	3.7 ± 0.9	0.6
		Ag-Ag	2.867 ± 0.004	11.0 ± 0.7	0.0090 ± 0.0005	0.0 ± 0.4	0.6
	CA at -0.6V vs RHE (last spectrum)	Cu-Cu (metal)	2.537 ± 0.005	6.8 ± 0.6	0.007 ± 0.001	2.7 ± 0.9	0.5
		Ag-Ag	2.877 ± 0.006	11.2 ± 0.9	0.009 ± 0.001	2.5 ± 0.6	1.1
$Cu_{0.75}Ag_{0.25}$	CV at -0.6V vs RHE	Cu-Cu (metal)	2.540 ± 0.005	7.6 ± 0.5	0.007 ± 0.001	3.2 ± 0.7	0.6
		Ag-Ag	2.867 ± 0.003	11.6 ± 0.5	0.009 ± 0.001	2.2 ± 0.3	0.2
	CA at -0.4V vs RHE (last spectrum)	Cu-Cu (metal)	2.540 ± 0.005	8.3 ± 0.6	0.007 ± 0.001	3.0 ± 0.8	0.4
		Ag-Ag	2.865 ± 0.004	11.7 ± 0.7	0.0100 ± 0.0006	1.3 ± 0.4	0.5
	CA at -0.6V vs RHE (last spectrum)	Cu-Cu (metal)	2.540 ± 0.006	8.3 ± 0.7	0.008 ± 0.001	3.5 ± 0.8	0.5
		Ag-Ag	2.871 ± 0.006	11.7 ± 1.0	0.009 ± 0.001	2.5 ± 0.6	1.2

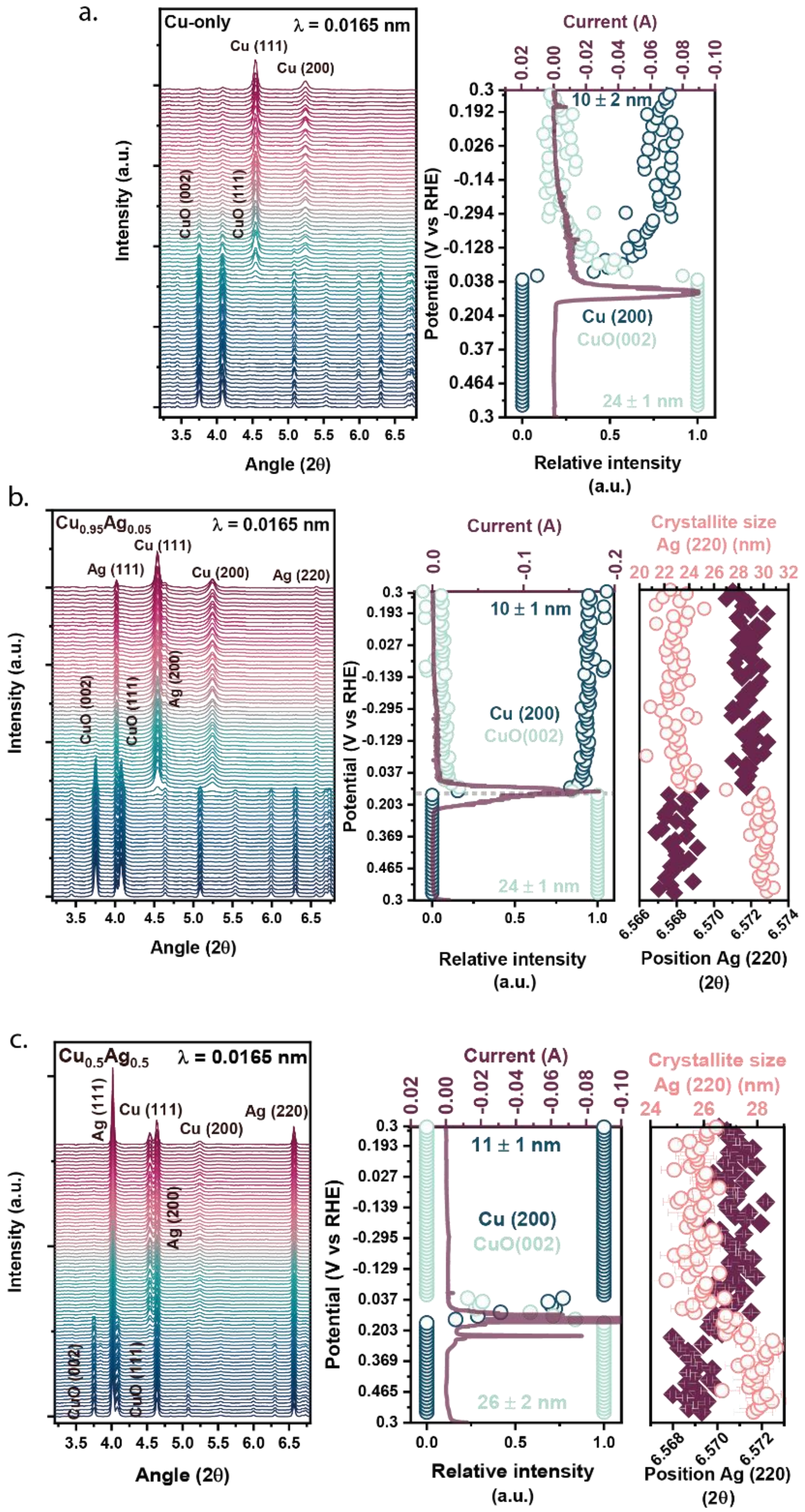

Figure S35. a) WAXS signal, CV and relative intensities of CuO (002) and Cu (200) reflections of Cu-only during the first CV in N_2, 3 M KOH, start +0.3 V vs RHE, from +0.5 V to -0.3 V vs RHE, 2 mV.s^{-1}, b) and c) WAXS signal, CV, relative intensities of CuO (002) and Cu (200) reflections and crystallite size and position of Ag (220) of b) $Cu_{0.95}Ag_{0.05}$ and c) $Cu_{0.5}Ag_{0.5}$ during the first CV in N_2, 3 M KOH, start +0.3 V vs RHE, from +0.5 V to -0.3 V vs RHE, 2 mV.s^{-1}.

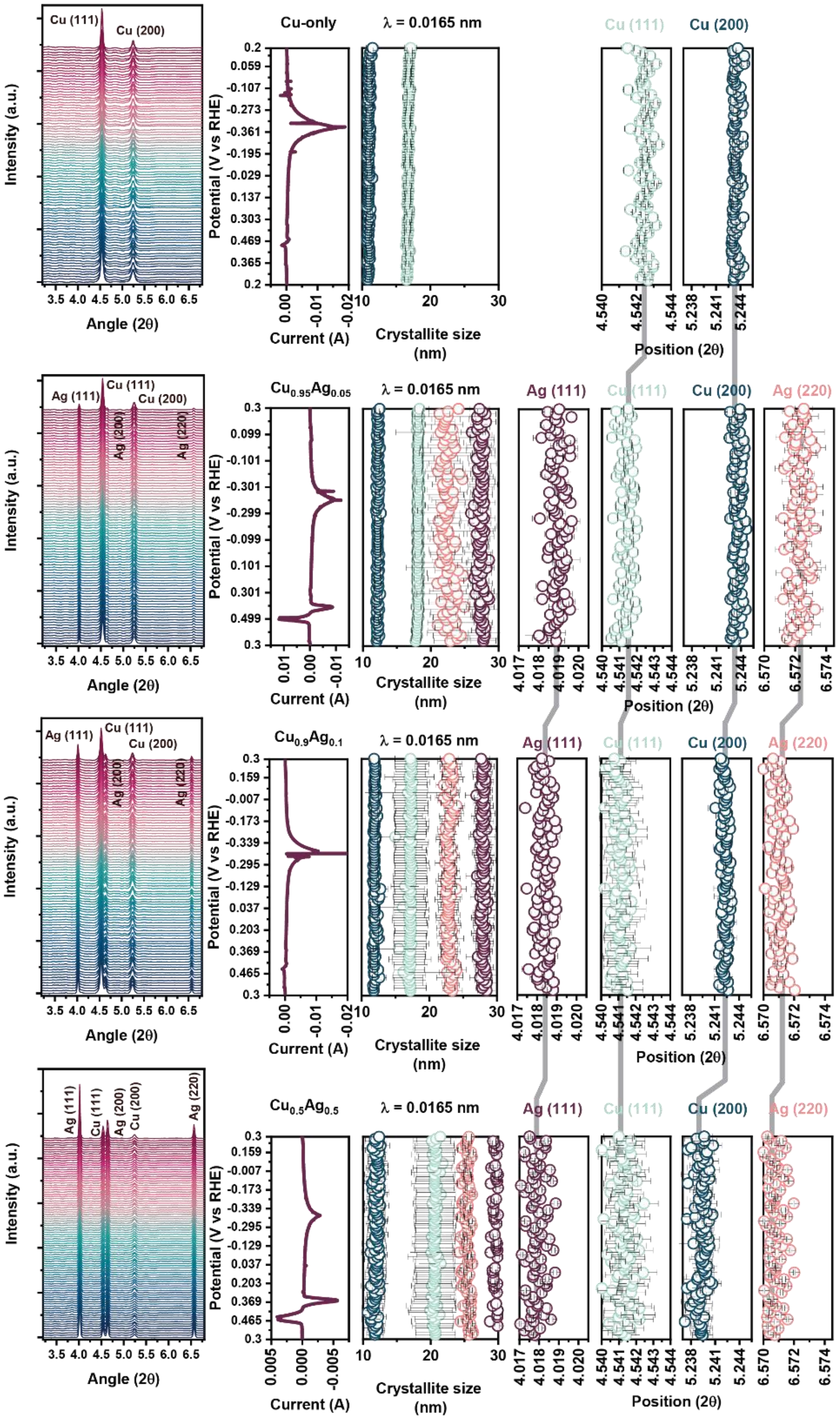

Figure S36. WAXS signal, CV, crystallite sizes and positions of important reflections of Cu-only, $Cu_{0.95}Ag_{0.05}$, $Cu_{0.9}Ag_{0.1}$ and $Cu_{0.5}Ag_{0.5}$ during the last CV cycle in nitrogen, 3 M KOH, start +0.3 V vs RHE, from +0.5 V to -0.4 V vs RHE, 2 mV.s^{-1}.

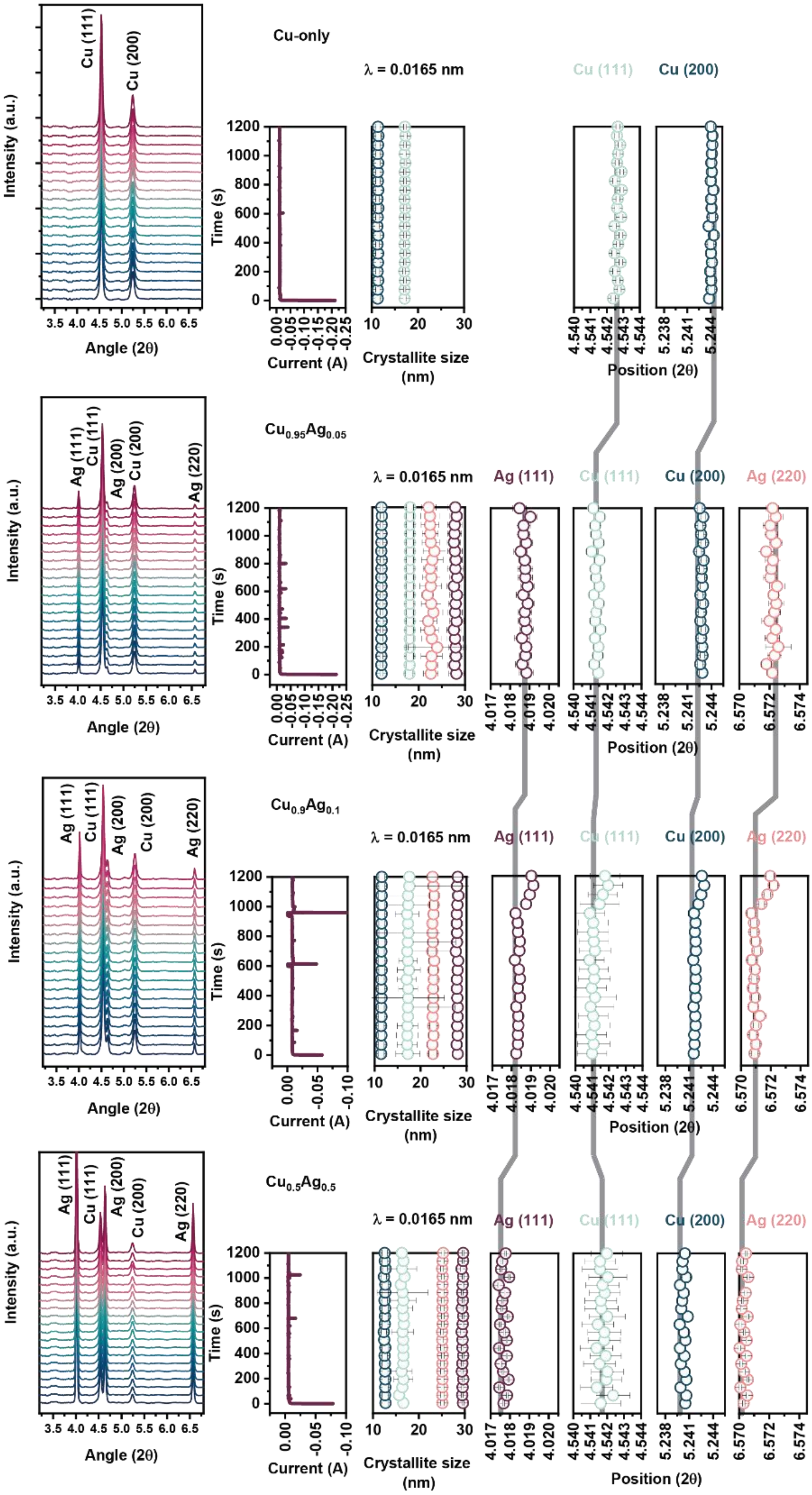

Figure S37. WAXS signal, CA, crystallite sizes and positions of important reflections of Cu-only, $Cu_{0.95}Ag_{0.05}$, $Cu_{0.9}Ag_{0.1}$ and $Cu_{0.5}Ag_{0.5}$ during the CA in CO at -0.4 V vs RHE, 3 M KOH.

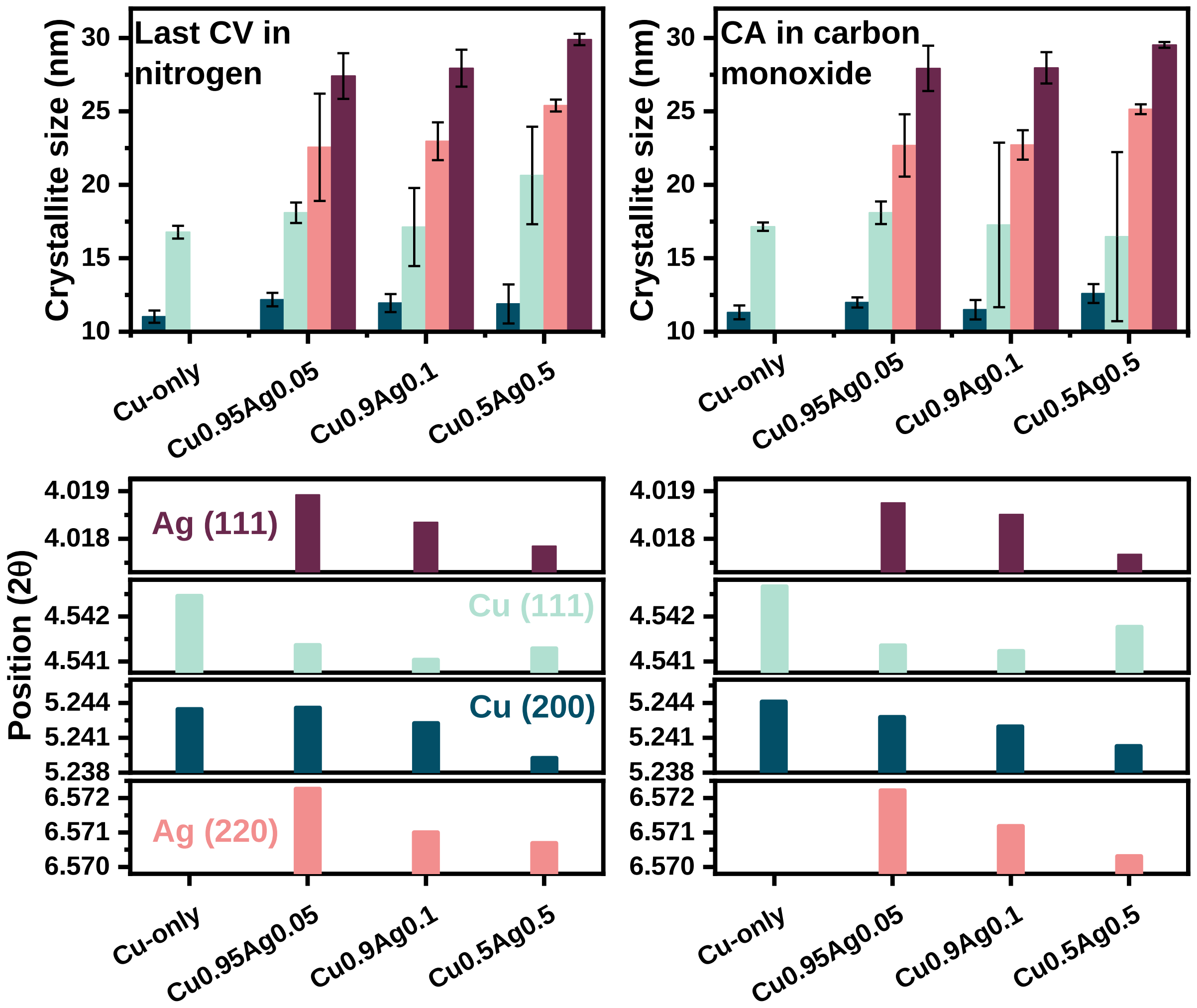

Figure S38. Summary of crystallite sizes and peak positions for important reflections: Ag (111) and Ag (220), Cu(111) and Cu(200) measured by in situ WAXS during the last CV in nitrogen (3 M KOH, start +0.3 V vs RHE, from +0.5 V to -0.4 V vs RHE, 2 $mV.s^{-1}$) and the CA in carbon monoxide at -0.4 V vs RHE.

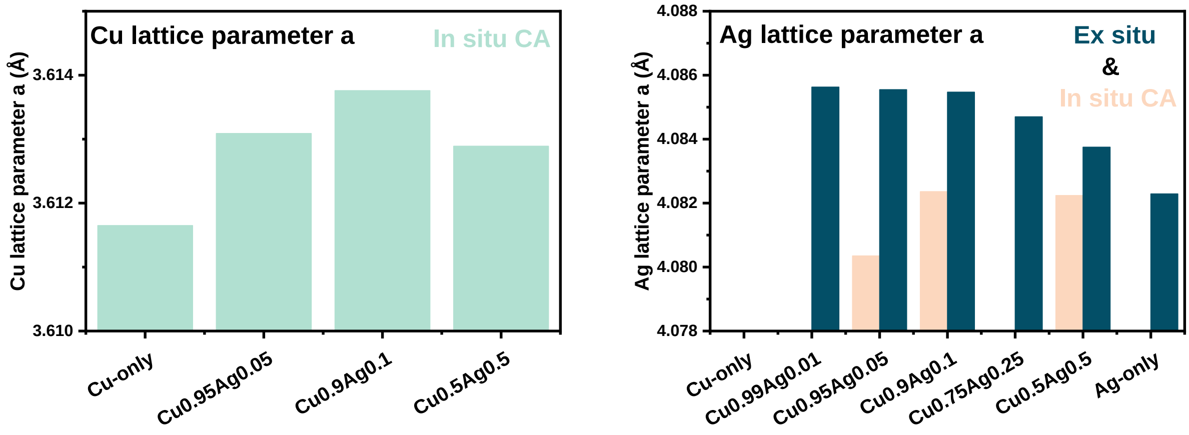

Figure S39. Rietveld refinement of FCC Cu and FCC Ag of samples during in situ WAXS measurements and chronoamperometry (CA) at -0.4 V vs RHE, CO, 3 M KOH – The analyzed diffractogram was chosen at half time of the total CA duration.

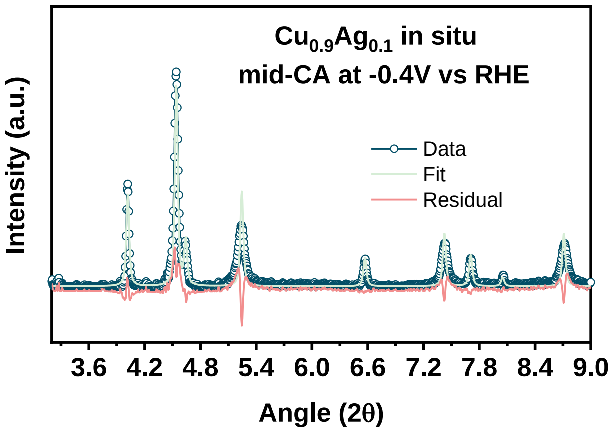

Figure S40. Rietveld refinement of $Cu_{0.9}Ag_{0.1}$ during CORR at -0.4 V vs RHE measured by in situ WAXS – Fit example.

Table S9. Rietveld refinement fitting parameters for as-synthesized samples (measured ex situ) and during CORR at -0.4 V vs RHE (measured in situ).

**Instrument parameters**	
λ (Å)	0.165312
Zero	0
Polarization	0.97
U	70.681
V	-6.339
W	0.926
X	1.167
Y	1.417
Z	0

**Ex situ**	**Cu-only**	**$Cu_{0.99}Ag_{0.01}$**	**$Cu_{0.95}Ag_{0.05}$**	**$Cu_{0.9}Ag_{0.1}$**	**$Cu_{0.75}Ag_{0.25}$**	**$Cu_{0.5}Ag_{0.5}$**	**Ag-only**
Background (number of coeff.)	Chebyschev (6)	Chebyschev (8)	Chebyschev (8)	Chebyschev (8)	Chebyschev (8)	Chebyschev (8)	Chebyschev (8)
CuO a	4.68068	4.68044	4.67994	4.68014	4.6805	4.67978	N/A
CuO b	3.42457	3.42455	3.42464	3.42488	3.42361	3.42293	N/A
CuO c	5.12791	5.12857	5.1286	5.12908	5.12864	5.12823	N/A
CuO β	99.359	99.352	99.36	99.363	99.418	99.449	N/A
Ag a	N/A	4.08563	4.08555	4.08547	4.0847	4.08375	4.08229
Phase fraction CuO	1	0.986	0.932	0.0862	0.68	0.434	0
Phase fraction Ag		0.014	0.068	0.138	0.32	0.566	1

**In situ**	**Cu-only**	**$Cu_{0.95}Ag_{0.05}$**	**$Cu_{0.9}Ag_{0.1}$**	**$Cu_{0.5}Ag_{0.5}$**
Background (number of coeff.)	Chebyschev (3)	Chebyschev (3)	Chebyschev (3)	Chebyschev (3)
Cu a	3.61587	3.61667	3.61818	3.61316
Ag a	N/A	4.08441	4.0873	4.08271
Phase fraction Cu	1	0.88	0.76	0.241
Phase fraction Ag	0	0.12	0.24	0.759

SI – References

(1) McCrory, C. C. L.; Jung, S.; Peters, J. C.; Jaramillo, T. F. Benchmarking Heterogeneous Electrocatalysts for the Oxygen Evolution Reaction. *J Am Chem Soc* **2013**, *135* (45), 16977–16987. https://doi.org/10.1021/JA407115P/SUPPL_FILE/JA407115P_SI_001.PDF.

(2) Hegde, C.; Sun, X.; Dinh, K. N.; Huang, A.; Ren, H.; Li, B.; Dangol, R.; Liu, C.; Wang, Z.; Yan, Q.; Li, H. Cu-And Fe-Codoped Ni Porous Networks as an Active Electrocatalyst for Hydrogen Evolution in Alkaline Medium. *ACS Appl Mater Interfaces* **2020**, *12* (2), 2380–2389. https://doi.org/10.1021/ACSAMI.9B17273/ASSET/IMAGES/LARGE/AM9B17273_0003.JPEG.

(3) Biesinger, M. C. Advanced Analysis of Copper X-Ray Photoelectron Spectra. *Surface and Interface Analysis* **2017**, *49* (13), 1325–1334. https://doi.org/10.1002/SIA.6239.

(4) Waterhouse, G. I. N.; Bowmaker, G. A.; Metson, J. B. Oxidation of a Polycrystalline Silver Foil by Reaction with Ozone. *Appl Surf Sci* **2001**, *183* (3–4), 191–204. https://doi.org/10.1016/S0169-4332(01)00561-X.

(5) Kibis, L. S.; Stadnichenko, A. I.; Pajetnov, E. M.; Koscheev, S. V.; Zaykovskii, V. I.; Boronin, A. I. The Investigation of Oxidized Silver Nanoparticles Prepared by Thermal Evaporation and Radio-Frequency Sputtering of Metallic Silver under Oxygen. *Appl Surf Sci* **2010**, *257* (2), 404–413. https://doi.org/10.1016/J.APSUSC.2010.07.002.

(6) Svintsitskiy, D. A.; Yu. Kardash, T.; Boronin, A. I. Surface Dynamics of Mixed Silver-Copper Oxide AgCuO2 during X-Ray Photoelectron Spectroscopy Study. *Appl Surf Sci* **2019**, *463*, 300–309. https://doi.org/10.1016/J.APSUSC.2018.08.234.

(7) La Fontaine, C.; Belin, S.; Barthe, L.; Roudenko, O.; Briois, V. ROCK: A Beamline Tailored for Catalysis and Energy-Related Materials from Ms Time Resolution to Mm Spatial Resolution. *Synchrotron Radiat News* **2020**, *33* (1), 20–25. https://doi.org/10.1080/08940886.2020.1701372.

(8) Briois, V.; La Fontaine, C.; Belin, S.; Barthe, L.; Moreno, T.; Pinty, V.; Carcy, A.; Girardot, R.; Fonda, E. ROCK: The New Quick-EXAFS Beamline at SOLEIL. *J Phys Conf Ser* **2016**, *712* (1), 012149. https://doi.org/10.1088/1742-6596/712/1/012149.

(9) *SAMBA French national synchrotron facility, Fastoch software*. https://www.synchrotron-soleil.fr/en/beamlines/samba (accessed 2024-05-01).

(10) *Matthew Newville, The University of Chicago, Software LARCH, 2022*. https://xraypy.github.io/xraylarch/ (accessed 2024-05-01).

(11) Vavra, J.; Shen, T. H.; Stoian, D.; Tileli, V.; Buonsanti, R. Real-Time Monitoring Reveals Dissolution/Redeposition Mechanism in Copper Nanocatalysts during the Initial Stages of the CO2 Reduction Reaction. *Angewandte Chemie International Edition* **2021**, *60* (3), 1347–1354. https://doi.org/10.1002/ANIE.202011137.
